# Supplementary material for: Selective Synthesis of Benzimidazoles from o-Phenylenediamine and Aldehydes Promoted by Supported Gold Nanoparticles
Source: Nanomaterials (Basel). 2020 Dec 1;10(12):2405. doi: 10.3390/nano10122405 (PMC7760220; doi:10.3390/nano10122405)

nanomaterials

Supplementary Materials

Selective Synthesis of Benzimidazoles from *o*-Phenylenediamine and Aldehydes Promoted by Supported Gold Nanoparticles

Marina A. Tzani [1], Catherine Gabriel [2] and Ioannis N. Lykakis [1,*]

[1] Department of Chemistry, Aristotle University of Thessaloniki, University Campus, 54124 Thessaloniki, Greece; marina_tzani@hotmail.com
[2] HERACLES Research Center, KEDEK, Laboratory of Environmental Engineering (EnvE-Lab), Department of Chemical Engineering, AUTH, 54124 Thessaloniki, Greece; katerinagabriel79@gmail.com
* Correspondence: lykakis@chem.auth.gr; Tel./Fax: +30-2310-997871

Table of Contents.

Pages	Supplementary Materials
**S1**	Table of Contents
**S2**	Scheme S1. Synthesis of benzimidazoles using metal oxides as heterogeneous catalysts
**S3**	Scheme S2. Synthesis of benzimidazoles under photocatalytic reactions.
**S4**	Table S1. Homogeneous catalyst screening for the reaction between **1** and **2**.
**S5**	Table S2. Heterogeneous surfaces screening for the reaction between **1** and **2**.
**S6**	Table S3. Solvent screening for the reaction between **1** and **2** catalyzed by Au/Al_2O_3
**S7-S17**	^{1}H and ^{13}C NMR data of 2-substituted-1*H*-1,3- benzodiazoles
**S18**	References
**S19-S54**	Copies of ^{1}H and ^{13}C NMR spectra of 2-substituted-1*H*-1,3- benzodiazoles

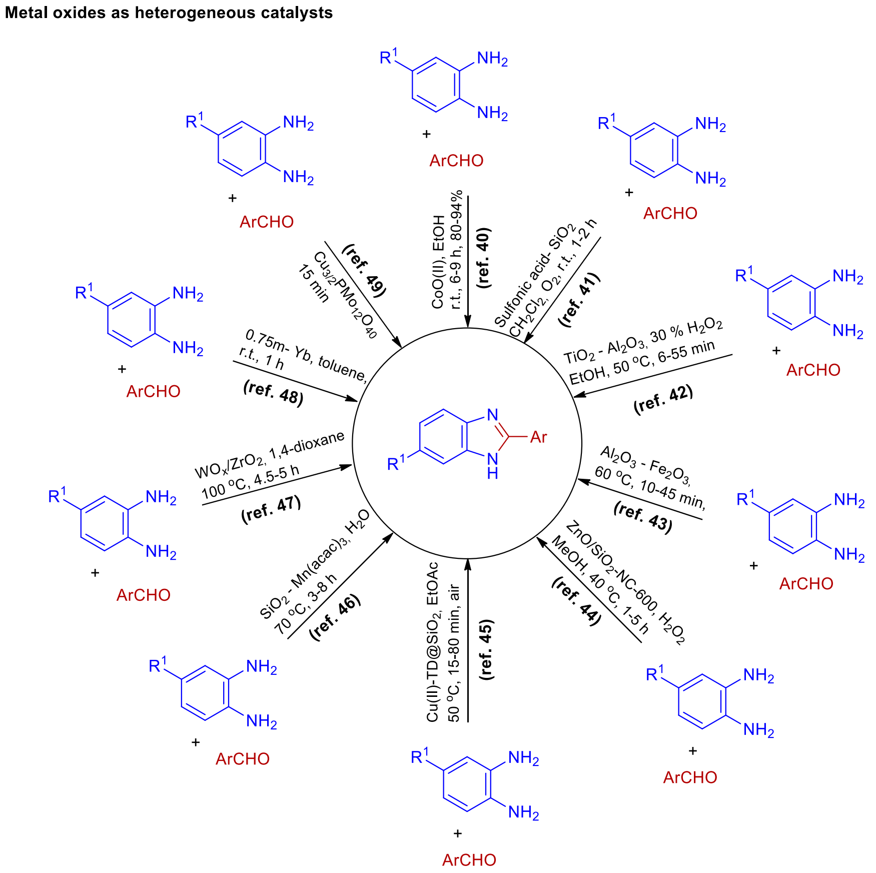

Scheme S1. Synthesis of benzimidazole derivatives using metal oxides as heterogeneous catalysts.

Visible-Light promoted synthesis

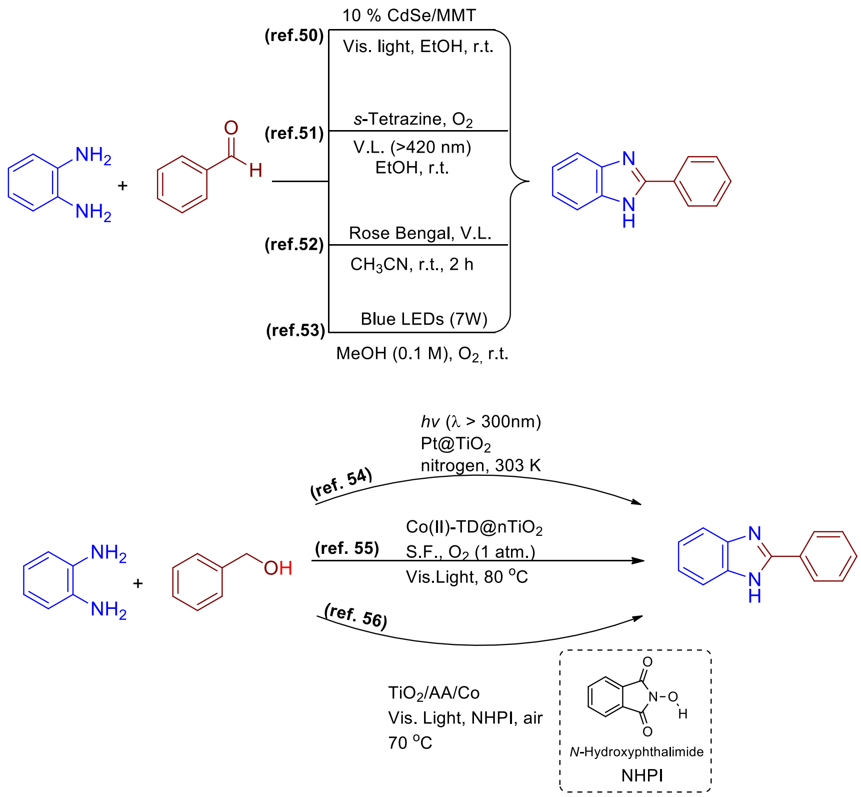

Scheme S2. Synthesis of benzimidazole derivatives under photocatalytic reactions.

Table S1. Homogeneous catalyst screening for the reaction between **1** and **2**.

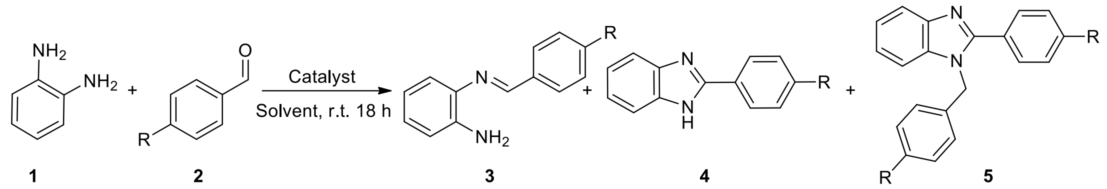

Entry	Catalyst[a]	Solvent	Conversion (%)[b]	3 (%)[c]	4 (%)[c]	5 (%)[c]
**1**	$HAuCl_4$	CH_3CN	100	-	32	68
**2**	$HAuCl_4$	CH_3OH	68	-	14	54
**3**	$HAuCl_4$	EtOH	100	-	66	34
**4**	$HAuCl_4$	1,2-DCE	85	51	29	5
**5**	$HAuCl_4$	EtOAc	71	-	57	14
**6**	$HAuCl_4$	Toluene	95	32	45	18
**7**	AuCl	CH_3CN	82	-	58	24
**8**	$AgNO_3$	MeOH	100	-	63	37
**9**	$Cu(ClO_4)_2 \bullet 6H_2O$	CH_3CN	100	-	40	60

[a] Reaction conditions: *o*-phenylenediamine **1** (0.1 mmol), 4-methylbenzaldehyde **2** (0.1 mmol), catalyst (20 % mol), solvent (1 ml), at room temperature for 18 h. [b] Based on the consumption of **2** determined from the crude ^1H NMR mixture of the reaction. [c] Yields of **3**, **4**, **5** were determined by ^1H NMR from the crude reaction mixture.

Table 2. Heterogeneous M_xO_y screening for the reaction between **1** and **2**.

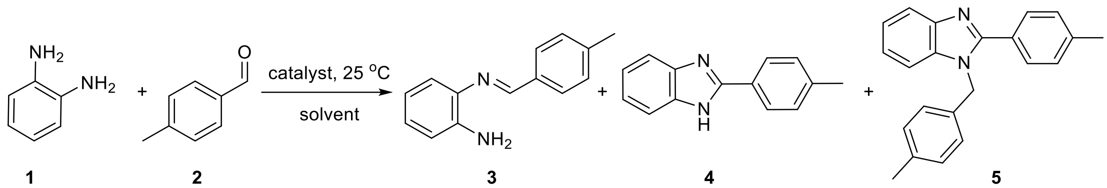

Entry	Catalyst	Solvent	Time (h)	2 (%)[a]	3 (%)[a]	4 (%)[a]	5 (%)[a]
1	SiO_2	CH_3CN	2	0	49	33	13
2	Al_2O_3	CH_3CN	2	0	44	38	18
3	Al_2O_3	CH_3CN	48	0	18	46	36
4	Al_2O_3	MeOH	48	0	-	40	60
5	TiO_2 (Degussa)	MeOH	2	0	-	41	59
6	TiO_2 (Degussa)	CH_3CN	2	0	-	46	54
7	TiO_2 (Hombikat)	CH_3CN	2	0	-	61	39
8[b]	hv/TiO_2 (Hombikat)	MeOH	1	0	-	65	35

[a] Reaction conditions: 0.1 mmol of **1**, 0.1 mmol of **2**, catalyst (20 mg), 1mL of solvent, at 25 °C. Yields of **2**, **3**, **4** and **5** were determined by ^{1}H NMR from the crude reaction mixture. [b] Reaction conditions: **1** (0.1 mmol), **2** (0.2 mmol), TiO_2 (20 mg), solvent (1 ml) at Xenon Lamp for 1 h.

Table S3. Solvent screening for the reaction between **1** and **2** catalyzed by Au/Al_2O_3.

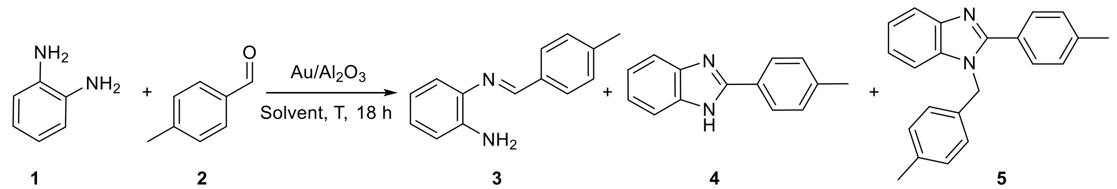

1 **2** **3** **4** **5**

Entry	Solvent[a]	Temperature (°C)	2 (%)[b]	3 (%)[b]	4 (%)[b]	5 (%)[b]
1	MeOH	r.t.	-	-	100	-
2	MeOH (repeat of 1)	r.t.	-	5	90	5
3	EtOAc	r.t.	10	33	30	27
4	CH_3CN	r.t.	-	16	50	34
5	Toluene	r.t.	-	100	-	-
6	Toluene	130	-	-	87	13

[a] Reaction conditions: *o*-phenylenediamine **1** (0.1 mmol), 4-methylbenzaldehyde **2** (0.1 mmol), catalyst (20 mg, 1 mol % Au loading), solvent (1 ml) for 18 h. [b] Yields of **2**, **3**, **4**, **5** were determined by ^1H NMR from the crude reaction mixture.

^{1}H and ^{13}C NMR data of 2-substituted-1*H*-1,3-benzodiazoles

2-(4-Methylphenyl)-1*H*-1,3-benzodiazole (4)[1],[2]: 62 mg, 99% yield, brown solid, m.p. 214-216 °C; ^{1}H NMR (500 MHz, CD_3OD) δ 7.96 (d, J = 8.0 Hz, 2H), 7.70 (dd, J_1 = 6.0 Hz, J_2 = 3.0 Hz, 2H), 7.45 (dd, J_1 = 6.0 Hz, J_2 = 3.0 Hz, 2H), 7.41 (d, J = 8.0 Hz, 2H), 2.40 (s, 3H); ^{13}C NMR (125 MHz, CD_3OD) δ 151.4, 145.2, 134.7, 131.4, 128.5, 126.6, 122.8, 115.0, 21.6; HRMS (ESI) $[M + H]^+$ calcd for $C_{14}H_{13}N_2$: 209.1073, found : 209.1075.

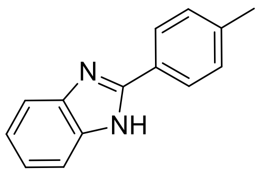

4

2-Phenyl-1*H*-1,3-benzodiazole (6)[1],[3]: 57 mg, 98% yield, brown solid, m.p. 242-244 °C; ^{1}H NMR (500 MHz, CD_3OD) δ 8.11 (dd, J_1 = 7.6 Hz, J_2 =1.5 Hz, 2H), 7.73 (dd, J_1 = 6.5 Hz, J_2 = 3.5 Hz, 2H), 7.65 (m, 3H), 7.47 (dd, J_1 = 8.0 Hz, J_2 = 3.0 Hz, 2H); ^{13}C NMR (125 MHz, CD_3OD) δ 151.5, 135.3, 133.8, 130.8, 128.6, 126.5, 126.2, 115.2.; HRMS (ESI) $[M + H]^+$ calcd for $C_{13}H_{11}N_2$: 195.0916, found : 195.0913.

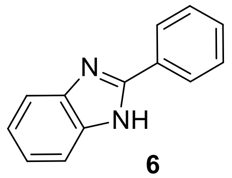

6

2-(2,6-Dimethylphenyl)-1*H*-1,3-benzodiazole (7)[5]: 43 mg, 65% yield, light yellow solid, m.p. 225-227 °C; ^{1}H NMR (500 MHz, CD_3OD) δ 7.60 (m, 2H), 7.28 (m, 3H), 7.17 (d, J = 7.5 Hz, 2H), 2.14 (s, 6H); ^{13}C NMR (125 MHz, CD_3OD) δ 153.2, 139.1, 132.3, 130.7, 128.5, 124.3, 123.6, 114.0 20.0; HRMS (ESI) $[M + H]^+$ calcd for $C_{15}H_{15}N_2$: 223.1230, found : 223.1239.

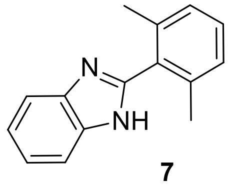

7

2-(2,4,6-Trimethylphenyl)-1*H*-1,3-benzodiazole (8)[6],[7]: 36 mg, 51% yield, yellow solid, m.p. 258-260 °C; ^{1}H NMR (500 MHz, $CDCl_3$) δ 7.32 (dd, J = 5.0, 3.0 Hz, 2H), 7.18 (dd, J = 5.5, 3.0 Hz, 2H), 6.79 (s, 2H), 2.31 (s, 3H), 1.89 (s, 6H); ^{13}C NMR (125 MHz, $CDCl_3$) δ 151.7, 139.5, 137.9, 137.6, 128.3, 128.0, 122.6, 114.9, 21.3, 19.8; HRMS (ESI) $[M + H]^+$ calcd for $C_{16}H_{17}N_2$: 237.1386, found : 237.1397.

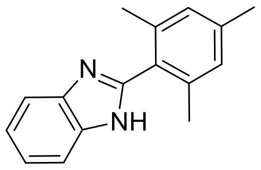

8

2-(2-Methoxyphenyl)-1*H*-1,3-benzodiazole (9)[8]: 66 mg, 98% yield, brown solid, m.p. 176-178 °C; ^{1}H NMR (500 MHz, $CDCl_3$) δ 8.59 (d, J = 8.0 Hz, 1H), 7.66 (br s, 2H), 7.42 (dd shown as t, J = 7.0 Hz, 1H), 7.26 (dd, J_1 = 6.0 Hz, J_2 = 3.5 Hz, 2H), 7.14 (dd shown as t, J = 7.5 Hz, 1H), 7.06 (d, J = 8.0 Hz, 1H), 4.07 (s, 3H); ^{13}C NMR (125 MHz, $CDCl_3$) δ 156.9, 150.0, 131.4, 130.3, 122.7, 121.9, 118.0, 111.6, 56.1; HRMS (ESI) [M + H]$^+$ calcd for $C_{14}H_{13}N_2O$: 225.1022, found : 225.1030.

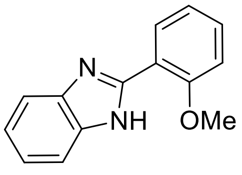

9

2-(3-Methoxyphenyl)-1*H*-1,3-benzodiazole (10)[9]: 67 mg, 99% yield, brown solid, m.p. 169-171 °C; ^{1}H NMR (500 MHz, $CDCl_3$) δ 9.18 (br s, 1H), 7.88 (s, 1H), 7.84 (d, J = 7.5 Hz, 1H), 7.70 (dd, J_1 = 6.5 Hz, J_2 = 3.5 Hz, 2H), 7.26 (dd, J_1 = 6.0 Hz, J_2 = 4.0 Hz, 2H), 7.11 (dd shown as t, J = 8.0 Hz, 1H), 6.83 (dd, J_1 = 8.5 Hz, J_2 = 2.0 Hz, 1H); ^{13}C NMR (125 MHz, $CDCl_3$) δ 160.2, 149.9, 134.2, 130.4, 126.0, 125.1, 120.1, 119.0, 114.5, 112.5, 55.7; HRMS (ESI) [M + H]$^+$ calcd for $C_{14}H_{13}N_2O$: 225.1022, found : 225.1034.

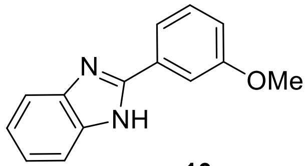

10

2-(4-Methoxyphenyl)-1*H*-1,3-benzodiazole (11)[1],[3]: 67 mg, 99% yield, brown solid, m.p. 223-225 °C; ^{1}H NMR (500 MHz, CD_3OD) δ 8.06 (d, J = 8.0 Hz, 2H), 7.72 (dd, J_1 = 6.5 Hz, J_2 = 3.5 Hz, 2H), 7.49 (dd, J_1 = 6.0 Hz, J_2 = 3.0 Hz, 2H), 7.17 (d, J = 9.0 Hz, 2H), 3.89 (s, 3H); ^{13}C NMR (125 MHz, CD_3OD) δ 165.1, 151.0, 133.9, 130.6, 126.8, 116.7, 116.3, 114.7, 56.3; HRMS (ESI) [M + H]$^+$ calcd for $C_{14}H_{13}N_2O$: 225.1022, found : 225.1024.

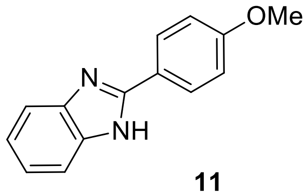

11

2-(3,4-Dimethoxyphenyl)-1*H*-1,3-benzodiazole (12)[8]: 75 mg, 98% yield, brown solid, m.p. 211-213 °C; ^{1}H NMR (500 MHz, CD_3OD) δ 7.68 (s, 1H), 7.66 (dd, J_1 = 8.5 Hz, J_2 = 1.5 Hz, 1H), 7.60 (dd, J_1 = 6.0 Hz, J_2 = 3.0, 2H), 7.31 (dd, J_1 = 6.0 Hz, J_2 = 3.0, 2H), 7.10 (d, J = 8.5 Hz, 1H), 3.94 (s, 3H), 3.90 (s, 3H); ^{13}C NMR (125 MHz, CD_3OD) δ 153.3, 152.8, 151.0, 138.0,

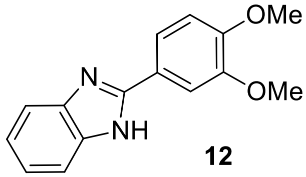

12

124.8, 121.5, 121.3, 115.3, 112.9, 111.2, 56.6, 56.5; HRMS (ESI) [M + H]$^+$ calcd for $C_{15}H_{15}N_2O_2$: 255.1128, found : 255.1126.

4-(1*H*-1,3-Benzodiazol-2-yl)phenol (13)[3]: 62 mg, 98% yield, brown solid, m.p. 259-261 °C; ^{1}H NMR (500 MHz, CD_3OD) δ 7.97 (d, J = 8.5 Hz, 2H), 7.67 (dd, J_1 = 6.0 Hz, J_2 = 3.0 Hz, 2H), 7.43 (dd, J_1 = 6.0 Hz, J_2 = 3.0 Hz, 2H), 7.02 (d, J = 8.5 Hz, 2H); ^{13}C NMR (125 MHz, CD_3OD) δ 163.5, 151.8, 134.6, 130.6, 126.3, 117.6, 116.2, 114.7. HRMS (ESI) [M + H]$^+$ calcd for $C_{13}H_{11}N_2O$: 211.0866, found : 211.0874.

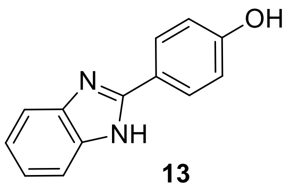

13

4-(1*H*-1,3-Benzodiazol-2-yl)-1,2-benzenediol (14)[10]: 66 mg, 98% yield, brown solid, m.p. 181-183 °C; ^{1}H NMR (500 MHz, CD_3OD) δ 7.65 (dd, J_1 = 6.0 Hz, J_2 = 3.0 Hz, 2H), 7.54 (d, J = 1.5 Hz, 1H), 7.48 (dd, J_1 = 8.5 Hz, J_2 = 2.0 Hz, 1H), 7.40 (dd, J_1 = 6.0 Hz, J_2 = 3.0 Hz, 2H), 6.98 (d, J = 8.0 Hz, 1H); ^{13}C NMR (125 MHz, CD_3OD) δ 152.4, 151.4, 147.6, 135.6, 125.8, 121.2, 117.6, 117.1, 115.3, 114.8; HRMS (ESI) [M + H]$^+$ calcd for $C_{13}H_{11}N_2O_2$: 227.0815, found : 227.0824.

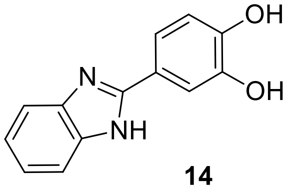

14

4-(1*H*-1,3-Benzodiazol-2-yl)-phenyl acetate (15): 69 mg, 91% yield, yellow solid, m.p. 245-247 °C; ^{1}H NMR (500 MHz, CD_3OD) δ 8.10 (d, J = 8.5 Hz, 2H), 7.60 (dd, J_1 = 6.0 Hz, J_2 = 3.0 Hz, 2H), 7.28 (d, J = 8.5 Hz, 2H), 7.25 (dd, J_1 = 6.0 Hz, J_2 = 3.0 Hz, 2H), 2.30 (s, 3H); ^{13}C NMR (125 MHz, CD_3OD) δ 170.8, 153.8, 152.6, 129.0, 128.6, 124.0, 123.6, 116.9, 116.7, 20.9; HRMS (ESI) [M + H]$^+$ calcd for $C_{15}H_{13}N_2O_2$: 253.0972, found : 253.0975.

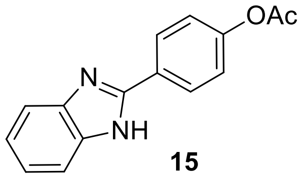

15

3-(1*H*-1,3-Benzodiazol-2-yl)aniline (16): 43 mg, 68% yield, brown solid, m.p. 182-184 °C; ^{1}H NMR (500 MHz, $CDCl_3$) δ 7.59 (dd, J_1 = 6.0 Hz, J_2 = 3.5 Hz, 2H), 7.46 (s, 1H), 7.39 (d, J = 7.5 Hz, 1H), 7.22 (dd, J_1 = 6.0 Hz, J_2 = 3.5 Hz, 2H), 7.17 (t, J = 7.5 Hz, 1H), 6.70 (dd, J_1 = 8.0 Hz, J_2 = 2.0 Hz, 1H); ^{13}C NMR

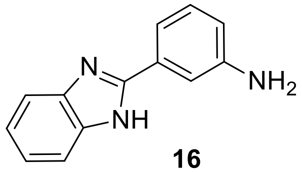

16

(125 MHz, $CDCl_3$) δ 152.2, 147.2, 130.8, 130.0, 122.9, 117.0, 116.7, 113.4; HRMS (ESI) $[M + H]^+$ calcd for $C_{13}H_{12}N_3$: 210.1026, found : 210.1024.

2-([1,1'-Biphenyl]-4-yl)-1*H*-1,3-benzodiazole (17)[2]: 65 mg, 80% yield, brown solid, m.p. 288-290 °C; ^{1}H NMR (500 MHz, CD_3OD) δ 8.17 (d, J = 8.5 Hz, 2H), 7.89 (d, J = 8.5 Hz, 2H), 7.75 (dd, J_1 = 6.0 Hz, J_2 = 3.0 Hz, 2H), 7.70 (d, J = 8.0 Hz, 2H), 7.51 – 7.46 (m, 4H), 7.40 (t, J = 7.5 Hz, 1H); ^{13}C NMR (125 MHz, CD_3OD) δ 151.3, 146.7, 140.4, 135.2, 130.2, 129.6, 129.1, 128.1, 126.6, 124.7, 115.2; HRMS (ESI) $[M + H]^+$ calcd for $C_{19}H_{15}N_2$: 271.1230, found : 271.1230.

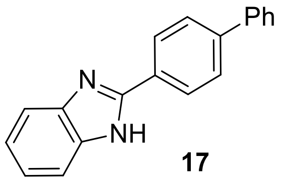

17

2-(4-Chlorophenyl)-1*H*-1,3-benzodiazole (18)[1],[3],[2]: 67 mg, 98% yield, light yellow solid, m.p. 288-289 °C; ^{1}H NMR (500 MHz, CD_3OD) δ 8.11 (d, J = 9.0 Hz, 1H), 7.77 (dd, J_1 = 6.0 Hz, J_2 = 3.0 Hz, 1H), 7.70 (d, J = 8.5 Hz, 1H), 7.53 (dd, J_1 = 6.0 Hz, J_2 = 3.0 Hz, 1H); ^{13}C NMR (125 MHz, CD_3OD) δ 152.2, 137.3, 132.1, 130.3, 129.7, 129.3, 129.1, 124.1; HRMS (ESI) $[M + H]^+$ calcd for $C_{13}H_{10}ClN_2$: 229.0527, found : 229.0528.

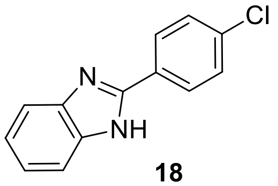

18

2-(3-Chlorophenyl)-1*H*-1,3-benzodiazole (19): 68 mg, 98% yield, orange semisolid; ^{1}H NMR (500 MHz, CD_3OD) δ 8.12 (d, J = 2.0 Hz, 1H), 8.02 (dt, J_1 = 7.0 Hz, J_2 = 1.5 Hz, 1H), 7.71 (dd, J_1 = 6.0 Hz, J_2 = 3.0 Hz, 2H), 7.62 (m, 2H), 7.45 (dd, J_1 = 6.0 Hz, J_2 = 3.0 Hz, 2H); ^{13}C NMR (125 MHz, CD_3OD) δ 150.2, 136.6, 135.8, 133.3, 132.3, 128.6, 128.4, 126.8, 126.5, 115.4; HRMS (ESI) $[M + H]^+$ calcd for $C_{13}H_{10}ClN_2$: 229.0527, found : 229.0528.

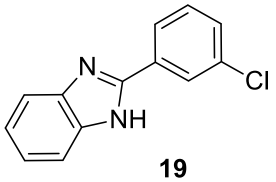

19

2-(2,6-Dichlorophenyl)-1*H*-1,3-benzodiazole (20): 77 mg, 96% yield, yellow solid, m.p. 247-249 °C; ^{1}H NMR (500 MHz, CD_3OD) δ 7.66 (dd, J_1 = 6.5 Hz, J_2 = 3.5 Hz, 2H), 7.57 – 7.51 (m, 3H), 7.34 (dd, J_1 = 6.0 Hz, J_2 = 3.5 Hz, 2H); ^{13}C NMR (125 MHz, CD_3OD) δ 148.2, 138.8, 137.2, 133.6, 130.6, 129.4,

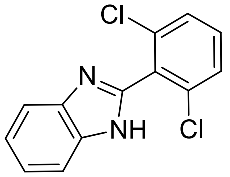

20

124.5, 116.2; HRMS (ESI) [M + H]$^+$ calcd for $C_{13}H_9Cl_2N_2$: 263.0137, found : 263.0143.

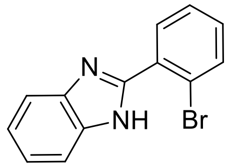

21

2-(2-Bromophenyl)-1*H*-1,3-benzodiazole (21): 73 mg, 89% yield, light yellow solid, m.p. 243-244 °C; ^{1}H NMR (600 MHz, CD_3OD) δ 7.79 (d, J = 7.8 Hz, 1H), 7.72 (d, J = 7.8 Hz, 1H), 7.64 (br s, 2H), 7.52 (t, J = 7.2 Hz, 1H), 7.44 (t, J = 7.8 Hz, 1H), 7.30 (dd, J_1 = 6.0 Hz, J_2 = 3.0 Hz, 2H); ^{13}C NMR (150 MHz, CD_3OD) δ 152.3, 134.7, 133.5, 133.2, 132.6, 128.8, 124.0, 123.2; HRMS (ESI) [M + H]$^+$ calcd for $C_{13}H_{10}BrN_2$: 273.0022/275.0002, found : 273.0027/275.0007.

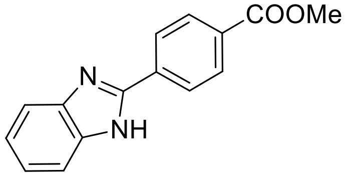

22

Methyl 4-(1*H*-1,3-benzodiazol-2-yl)benzoate (22): 67 mg, 88% yield, light yellow solid, m.p. 196-198 °C; ^{1}H NMR (500 MHz, CD_3OD) δ 8.19 – 8.14 (m, 4H), 7.70 (dd, J_1 = 6.0 Hz, J_2 = 3.0 Hz, 2H), 7.42 (dd, J_1 = 6.5 Hz, J_2 = 3.5 Hz, 2H), 3.93 (s, 3H); ^{13}C NMR (125 MHz, CD_3OD) δ 167.3, 150.8, 137.1, 133.9, 131.9, 131.4, 128.3, 126.0, 115.7, 53.0; HRMS (ESI) [M + H]$^+$ calcd for $C_{15}H_{13}N_2O_2$: 253.0972, found : 253.0973.

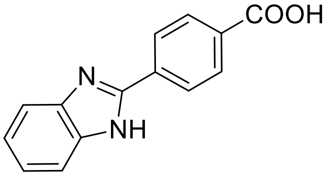

23

4-(1*H*-1,3-Benzodiazol-2-yl)benzoic acid (23): 70 mg, 98% yield, light yellow solid, m.p. 295-297 °C; ^{1}H NMR (500 MHz, DMSO) δ 8.29 (d, J = 7.5 Hz, 2H), 8.11 (d, J = 8.0 Hz, 2H), 7.63 (dd shown as s, 2H), 7.25 – 7.20 (dd shown as m, 2H). ^{13}C NMR (125 MHz, DMSO) δ 166.7, 148.7, 135.1, 133.4, 130.2, 129.9, 127.7, 124.8, 114.8; HRMS (ESI) [M + H]$^+$ calcd for $C_{14}H_{11}N_2O_2$: 239.0815, found : 239.0813.

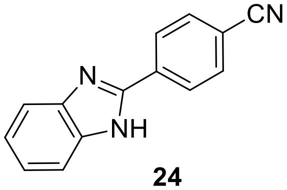

24

4-(1*H*-1,3-Benzodiazol-2-yl)benzonitrile (24)[2]: 65 mg, 98% yield, orange solid, m.p. 264-266 °C; ^{1}H NMR (500 MHz, CD_3OD) δ 8.26 (d, J = 8.5 Hz, 2H), 7.99 (d, J = 8.5 Hz, 2H), 7.75 (dd, J_1 = 6.0 Hz, J_2 = 3.0 Hz, 2H), 7.47 (dd, J_1 = 6.0 Hz, J_2 = 3.0 Hz, 2H); ^{13}C NMR (125 MHz, CD_3OD) δ 150.1, 137.0,

134.4, 131.9, 129.0, 126.4, 118.9, 116.1, 115.8; HRMS (ESI) $[M + H]^+$ calcd for $C_{14}H_{10}N_3$: 220.0869, found : 220.0869.

2-(3-Nitrophenyl)-1*H*-1,3-benzodiazole (25)[3]: 70 mg, 97% yield, brown solid, m.p. 180-181 °C; ^{1}H NMR (500 MHz, $CDCl_3$) δ 8.86 (s, 1H), 8.51 (d, J = 8.0 Hz, 1H), 8.19 (dd, J_1 = 8.0 Hz, J_2 = 1.0 Hz, 1H), 7.69 (dd, J_1 = 6.0 Hz, J_2 = 3.0 Hz, 2H), 7.58 (dd shown as t, J = 8.0 Hz, 1H), 7.30 (dd, J_1 = 6.0 Hz, J_2 = 3.0 Hz, 2H); ^{13}C NMR (125 MHz, $CDCl_3$) δ 149.4, 148.7, 132.8, 131.6, 130.3, 124.6, 123.9, 121.4, 115.7; HRMS (ESI) $[M + H]^+$ calcd for $C_{13}H_{10}N_3O_2$: 240.0767, found : 240.0768.

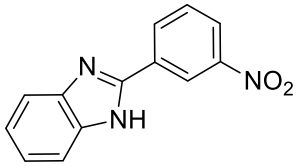

25

2-(Naphthalen-1-yl)-1*H*-1,3-benzodiazole (26)[3]: 63 mg, 86% yield, white solid, m.p. 298-300 °C; ^{1}H NMR (500 MHz, CD_3OD) δ 8.46 (m, 1H), 8.05 (d, J = 8.5 Hz, 1H), 7.99 (m, 1H), 7.87 (dd, J_1 = 7.0 Hz, J_2 = 1.0 Hz, 1H), 7.68 (br, 2H), 7.63 (dd, J_1 = 6.5 Hz, J_2 = 1.0 Hz, 1H), 7.55 –7.59 (m, 2H), 7.32 (m, 2H); ^{13}C NMR (125 MHz, CD_3OD) δ 153.3, 135.4, 132.6, 131.6, 129.6, 129.29, 129.28, 128.2, 127.5, 126.7, 126.1, 123.9, 116.3; ^{1}H NMR (500 MHz, DMSO-d_6) δ 9.14 (d, J = 8.5 Hz, 1H), 8.09 (d, J = 8.5 Hz, 1H), 8.03 (t, J = 8.5 Hz, 2H), 7.68 (m, 3H), 7.65-7.60 (m, 2H); ^{13}C NMR (125 MHz, DMSO-d_6) δ 151.6, 133.6, 130.5, 130.1, 128.4, 127. 9, 127.7, 127.0, 126.4, 126.3, 125.3, 122.0, 115.2; HRMS (ESI) $[M + H]^+$ calcd for $C_{17}H_{13}N_2$: 245.1073, found : 245.1079.

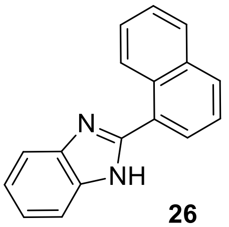

26

2-(Furan-2-yl)-1H-1,3-benzodiazole (27)[1],[3]: 44 mg, 80% yield, dark brown solid, m.p. 221-223 °C; ^{1}H NMR (500 MHz, CD_3OD) δ 7.88 (dd shown as s, 1H), 7.63 (dd, J_1 = 5.5 Hz, J_2 = 3.0 Hz, 2H), 7.38 (dd, J_1 = 6.0 Hz, J_2 = 3.0 Hz, 3H), 6.75 (dd shown as d, J = 1.5 Hz, 1H); ^{13}C NMR (125 MHz, CD_3OD) δ 147.7, 143.6, 143.3, 136.3, 125.8, 115.3, 115.1, 113.9; HRMS (ESI) $[M + H]^+$ calcd for $C_{11}H_9N_2O$: 185.0709, found : 185.0708.

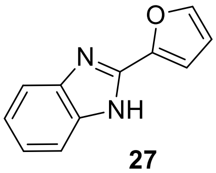

27

[5-(1*H*-1,3-Benzodiazol-2-yl)furan-2-yl]methanol (28)[11]: 61 mg, 95% yield, dark brown solid, m.p. 203-205 °C; ^{1}H NMR (500 MHz, CD_3OD) δ 7.60 (dd, J_1 = 6.0 Hz, J_2 = 3.5 Hz, 2H), 7.34 (dd, J_1 = 6.5 Hz, J_2 = 3.5 Hz, 2H), 7.27 (d, J = 3.5 Hz, 1H), 6.60 (d, J = 3.5 Hz, 1H), 4.66 (s, 2H); ^{13}C NMR (125 MHz, CD_3OD) δ 159.9, 144.0, 143.5, 137.2, 125.3, 115.4, 115.1, 111.2, 57.4; HRMS (ESI) $[M + H]^+$ calcd for $C_{12}H_{11}N_2O_2$: 215.0815, found : 215.0818.

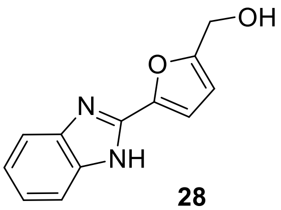

28

2-(5-Bromofuran-2-yl)-1*H*-1,3-benzodiazole (29): 74 mg, 95% yield, brown oil,; ^{1}H NMR (600 MHz, CD_3OD) δ 7.56 (dd, J_1 = 4.8 Hz, J_2 = 3.0 Hz, 2H), 7.25 (dd, J_1 = 6.0 Hz, J_2 = 3.0 Hz, 2H), 7.15 (d, J = 3.6 Hz, 1H), 6.56 (d, J = 3.6 Hz, 1H); ^{13}C NMR (150 MHz, CD_3OD) δ 148.4, 144.1, 125.5, 124.3, 116.0, 115.3, 114.1; HRMS (ESI) $[M + H]^+$ calcd for $C_{11}H_8BrN_2O$: 262.9815/264.9794, found : 262.9814/264.9793.

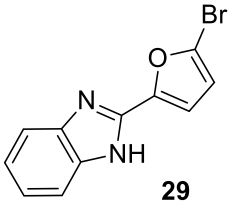

29

2-(thiophen-2-yl)-1*H*-1,3-benzodiazole (30)[1],[3]: 56 mg, 95% yield, brown solid, m.p. 215-217 °C; ^{1}H NMR (500 MHz, CD_3OD) δ 8.04 (d, J = 3.5 Hz, 1H), 7.93 (d, J = 5.0 Hz, 1H), 7.68 (dd, J_1 = 6.0 Hz, J_2 = 3.0 Hz, 2H), 7.46 (dd, J_1 = 6.0 Hz, J_2 = 3.0 Hz, 2H), 7.34 (dd shown as t, J = 4 Hz , 1H); ^{13}C NMR (125 MHz, CD_3OD) δ 146.7, 134.9, 133.7, 132.61, 132.6, 130.2, 127.6, 126.6, 114.9; HRMS (ESI) $[M + H]^+$ calcd for $C_{11}H_9N_2S$: 201.0480, found : 201.0478.

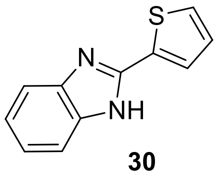

30

3-(1*H*-1,3-Benzodiazol-2-yl)-6-isopropyl-4*H*-chromen-4-one (31): 88 mg, 96% yield, yellow solid, m.p. 225-227 °C; ^{1}H NMR (500 MHz, $CDCl_3$) δ 11.88 (br s, 1H), 9.35 (s, 1H), 8.18 (d, J = 2.0 Hz, 1H), 7.64 (dd, J_1 = 8.5 Hz, J_2 = 2.0 Hz, 3H), 7.52 (d, J = 8.5 Hz, 1H), 7.28 (dd, J_1 = 6.0 Hz, J_2 = 3.0 Hz, 2H), 3.03 – 305 (m, 1H), 1.34 (s, 3H), 1.33 (s, 3H); ^{13}C NMR (125 MHz, $CDCl_3$) δ 176.7, 157.5, 154.7, 147.4, 146.4, 133.7, 123.8, 123.0, 122.8,

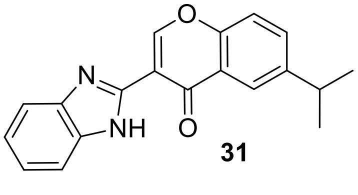

31

118.6, 113.7, 34.0, 24.0 (2C); HRMS (ESI) $[M + H]^+$ calcd for $C_{19}H_{17}N_2O_2$: 305.1284, found : 305.1281.

(*E*)-2-(2-Phenylethenyl)-1*H*-1,3-benzodiazole (32)[3]: 63 mg, 96% yield, light yellow solid, m.p. 164-166 °C; ^{1}H NMR (500 MHz, CD_3OD) δ 7.60 – 7.56 (m, 3H), 7.53 (dd, J_1 = 6.0 Hz, J_2 = 3.5 Hz, 2H), 7.36 (t, J = 7.5 Hz, 2H), 7.31 (d, J = 7.0 Hz, 1H), 7.22 (dd, J_1 = 6.0 Hz, J_2 = 3.0 Hz, 2H), 7.13 (d, J = 16.5 Hz, 1H); ^{13}C NMR (125 MHz, CD_3OD) δ 152.5, 137.1, 136.9, 130.2, 130.1, 129.9, 128.1, 124.0, 117.2, 115.6; HRMS (ESI) $[M + H]^+$ calcd for $C_{15}H_{13}N_2$: 221.1063, found : 221.1054.

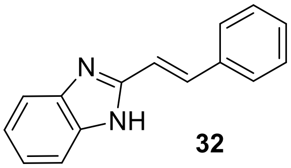

32

2-(1-Phenylethyl)-1*H*-1,3-benzodiazole (33): 65 mg, 98% yield, white solid, m.p. 208-210 °C; ^{1}H NMR (600 MHz, $CDCl_3$) δ 7.49 (dd shown as brs, 2H), 7.30 (d, J = 3.6 Hz, 4H), 7.21 – 7.20 (m, 1H), 7.18 (dd, J_1 = 6.0 Hz, J_2 = 3.6 Hz, 2H), 4.41 (dd, J_1 = 14.4 Hz, J_2 = 7.2 Hz, 1H), 1.78 (d, J = 7.2 Hz, 3H); ^{13}C NMR (150 MHz, $CDCl_3$) δ 159.2, 144.4, 129.8, 128.3, 128.0, 123.3, 41.3, 20.6; HRMS (ESI) $[M + H]^+$ calcd for $C_{15}H_{15}N_2$: 223.1230, found : 223.1230.

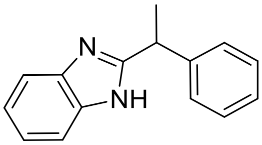

33

2-(2-Phenylcyclopropyl)-1*H*-1,3-benzodiazole (34): 62 mg, 88% yield, orange semisolid; ^{1}H NMR (600 MHz, $CDCl_3$) δ 7.52 (dd shown as br s, 2H), 7.24 (t, J = 7.2 Hz, 2H), 7.20 – 7.17 (m, 3H), 7.03 (d, J = 7.2 Hz, 2H), 2.68 – 2.65 (m, 1H), 2.28 (m, 1H), 2.00 (m, 1H), 1.51 (m, 1H); ^{13}C NMR (150 MHz, $CDCl_3$) δ 155.7, 140.7, 128.6, 126.4, 126.0, 122.3, 114.6 27.6, 21.5, 17.7; HRMS (ESI) $[M + H]^+$ calcd for $C_{16}H_{15}N_2$: 235.1230, found : 235.1233.

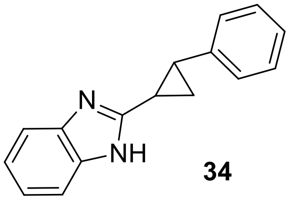

34

2-(Isopropyl)-1*H*-1,3-benzodiazole (35)[1],[12]: 42 mg, 87% yield, yellow solid, m.p. 160-162 °C; ^{1}H NMR (600 MHz, $CDCl_3$) δ 7.55 (br s, 2H), 7.20 (dd, J_1 = 5.8 Hz, J_2 = 3.0 Hz, 2H), 3.29 (m, 1H), 1.47 (d, J = 6.6 Hz, 6H); ^{13}C NMR (150 MHz,

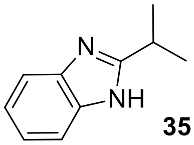

35

$CDCl_3$) δ 160.2, 122.2, 114.7, 29.2, 21.7; HRMS (ESI) $[M + H]^+$ calcd for $C_{10}H_{13}N_2$: 161.1073, found : 161.1074.

2-(*sec*-Butyl)-1*H*-1,3-benzodiazole (36)[12]: 42 mg, 80% yield, white solid, m.p. 206-208 °C; ^{1}H NMR (600 MHz, $CDCl_3$) δ 10.2 (br s, 1H), 7.55 (dd, J_1 = 5.4 Hz, J_2 = 3.0 Hz, 2H), 7.20 (dd, J_1 = 6.0 Hz, J_2 = 3.0 Hz, 2H), 3.13 – 3.07 (m, 1H), 1.97 – 1.93 (m, 1H), 1.79 – 1.74 (m, 1H), 1.45 (d, J = 6.6 Hz, 3H), 0.92 (t, J = 7.8 Hz, 3H); ^{13}C NMR (150 MHz, $CDCl_3$) δ 159.8, 138.4, 122.2, 114.8, 36.6, 29.5, 19.6, 12.1; HRMS (ESI) $[M + H]^+$ calcd for $C_{11}H_{14}N_2$: 175.1230, found : 175.1231.

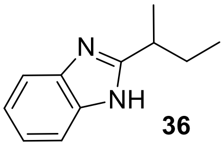
36

2-Butyl-1*H*-1,3-benzodiazole (37)[12]: 49 mg, 95% yield, light brown solid, m.p. 148-151 °C; ^{1}H NMR (500 MHz, $CDCl_3$) δ 10.44 (br s, 1H), 7.54 (dd, J_1 = 5.5 Hz, J_2 = 3.0 Hz, 2H), 7.21 (dd, J_1 = 5.5 Hz, J_2 = 3.0 Hz, 2H), 2.94 (t, J = 8.0 Hz, 2H), 1.82 (dd, J_1 = 15.0 Hz, J_2 = 7.5 Hz, 2H), 1.36 (dd, J_1 = 15.0 Hz, J_2 = 7.5 Hz, 2H), 0.86 (t, J = 7.0 Hz, 3H); ^{13}C NMR (125 MHz, $CDCl_3$) δ 155.6, 138.1, 122.4, 114.6, 30.5, 28.9, 22.5, 13.8; HRMS (ESI) $[M + H]^+$ calcd for $C_{11}H_{15}N_2$: 175.1230, found : 175.1248.

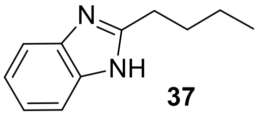
37

2-Methyl-1*H*-1,3-benzodiazole (38)[13]: 39 mg, 98% yield, yellow oil; ^{1}H NMR (500 MHz, CD_3OD) δ 7.49 (dd, J_1 = 6 Hz, J_2 = 3 Hz, 2H), 7.12 (dd, J_1 = 6 Hz, J_2 = 3 Hz, 2H), 2.53 (s, 3H); ^{13}C NMR (125 MHz, CD_3OD) δ 151.3, 121.1, 14.6; HRMS (ESI) $[M + H]^+$ calcd for $C_8H_9N_2$: 133.0760, found : 133.0761.

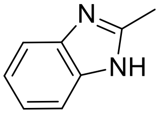
38

2-Nonyl-1*H*-1,3-benzodiazole (39): 68 mg, 93% yield, colorless oil; ^{1}H NMR (600 MHz, $CDCl_3$) δ 10.20 (s, 1H), 7.54 (dd, J = 5.8, 3.1 Hz, 2H), 7.20 (dd, J = 5.9, 3.0 Hz, 2H), 2.90 (t, J = 7.7 Hz, 2H), 1.81 (dt, J = 15.3, 7.7 Hz, 2H), 1.30 (d, J = 7.4 Hz, 2H), 1.25 (d, J = 6.2 Hz, 4H), 1.18 (s, 4H), 0.85 (t, J = 7.2 Hz, 3H); ^{13}C NMR (150 MHz, $CDCl_3$) δ 155.47, 137.55, 122.62,

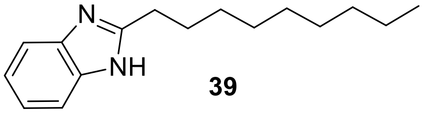
39

114.55, 77.37, 77.16, 76.95, 31.96, 29.57, 29.46, 29.44, 29.39, 28.97, 28.42, 22.76, 14.21; HRMS (ESI) [M + H]$^+$ calcd for $C_{16}H_{25}N_2$: 245.2012, found : 245.2017.

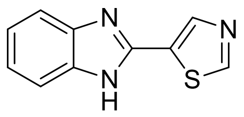

44

2-(1,3-Thiazol-5-yl)-1*H*-1,3-benzodiazole (44): 55 mg, 92% yield, yellow semi-solid; ^{1}H NMR (600 MHz, CD_3OD) δ 9.10 (s, 1H), 8.46 (s, 1H), 7.56 (dd, J_1 = 6.0 Hz, J_2 = 3.0 Hz, 2H), 7.25 (dd, J_1 = 6.0 Hz, J_2 = 3.0 Hz, 2H); ^{13}C NMR (150 MHz, CD_3OD) δ 157.3, 145.7, 143.1, 130.4, 124.5, 124.3, 116.0; HRMS (ESI) [M + H]$^+$ calcd for $C_{10}H_7N_3S$: 202.0433, found : 202.0440

References

S1. Kim, Y.; Kumar, M.R.; Park, N.; Heo, Y.; Lee, S. Copper-Catalyzed, One-Pot, Three-Component Synthesis of Benzimidazoles by Condensation and C–N Bond Formation. *J. Org. Chem.* **2011**, *76*, 9577–9583, doi:10.1021/jo2019416.

S2. Elumalai, V.; Hansen, J.H. A Green, Scalable, One-Minute Synthesis of Benzimidazoles. *Synlett* **2020**, *31*, 547–552, doi:10.1055/s-0039-1690797.

S3. Wang, Z.; Song, T.; Yang, Y. Additive- and Oxidant-Free Expedient Synthesis of Benzimidazoles Catalyzed by Cobalt Nanocomposites on N-Doped Carbon. *Synlett* **2019**, *30*, 319–324, doi:10.1055/s-0037-1610353.

S4. Shiraishi, Y.; Sugano, Y.; Tanaka, S.; Hirai, T. One-Pot Synthesis of Benzimidazoles by Simultaneous Photocatalytic and Catalytic Reactions on Pt@TiO2 Nanoparticles. *Angew. Chem. Int. Ed.* **2010**, *122*, 1700–1704, doi:10.1002/ange.200906573.

S5. Reddy, P.L.; Arundhathi, R.; Tripathi, M.; Rawat, D.S. CuI nanoparticles mediated expeditious synthesis of 2-substituted benzimidazoles using molecular oxygen as the oxidant. *RSC Adv.* **2016**, *6*, 53596–53601, doi:10.1039/C6RA11678G.

S6. Khoshyan, A.; Pourtahmasb, M.; Feizpour, F.; Jafarpour, M.; Rezaeifard, A. Aerobic {$Mo_{72}V_{30}$} nanocluster-catalysed heterogeneous one-pot tandem synthesis of benzimidazoles. *Appl. Organometal. Chem.* **2018**, e4638, doi:10.1002/aoc.4638.

S7. Wright, A.G.; Weissbach, T.; Holdcroft, S. Poly(phenylene) and *m* -Terphenyl as Powerful Protecting Groups for the Preparation of Stable Organic Hydroxides. *Angew. Chem. Int. Ed.* **2016**, *55*, 4818–4821, doi:10.1002/anie.201511184.

S8. Maphupha, M.; Juma, W.P.; de Koning, C.B.; Brady, D. A modern and practical laccase-catalysed route suitable for the synthesis of 2-arylbenzimidazoles and 2-arylbenzothiazoles. *RSC Adv.* **2018**, *8*, 39496–39510, doi:10.1039/C8RA07377E.

S9. Nguyen, T.B.; Le Bescont, J.; Ermolenko, L.; Al-Mourabit, A. Cobalt- and Iron-Catalyzed Redox Condensation of *o* -Substituted Nitrobenzenes with Alkylamines: A Step- and Redox-Economical Synthesis of Diazaheterocycles. *Org. Lett.* **2013**, *15*, 6218–6221, doi:10.1021/ol403064z.

S10. Han, F.; Zhang, X.; Zhang, J.; Wei, Y.; Zhang, X.; Huang, W.; Xu, H. 3D-Encapsulated iridium-complexed nanophosphors for highly efficient host-free organic light-emitting diodes. *Chem. Commun.* **2016**, *52*, 5183–5186, doi:10.1039/C6CC01414C.

S11. Gensberger, S.; Knabner, C.; Waibel, R.; Huppert, J.; Pischetsrieder, M. Qualitative Profiling of Polyglucose Degradation Products in Peritoneal Dialysis Fluids. *Anal. Chem.* **2015**, *87*, 6103–6111, doi:10.1021/acs.analchem.5b00665.

S12. Zhang, C.; Zhang, L.; Jiao, N. Catalyst free approach to benzimidazoles using air as the oxidant at room temperature. *Green Chem.* **2012**, *14*, 3273, doi:10.1039/c2gc36416f.

S13. Wade, A.R.; Pawar, H.R.; Biware, M.V.; Chikate, R.C. Synergism in semiconducting nanocomposites: visible light photocatalysis towards the formation of C–S and C–N bonds. *Green Chem.* **2015**, *17*, 3879–3888, doi:10.1039/C5GC00748H.

Copies of ^{1}H and ^{13}C NMR spectra of 2-substituted-1H-1,3- benzodiazoles

CD$_3$OD, 500MHz

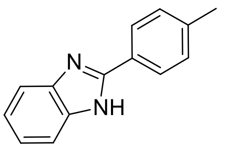

4

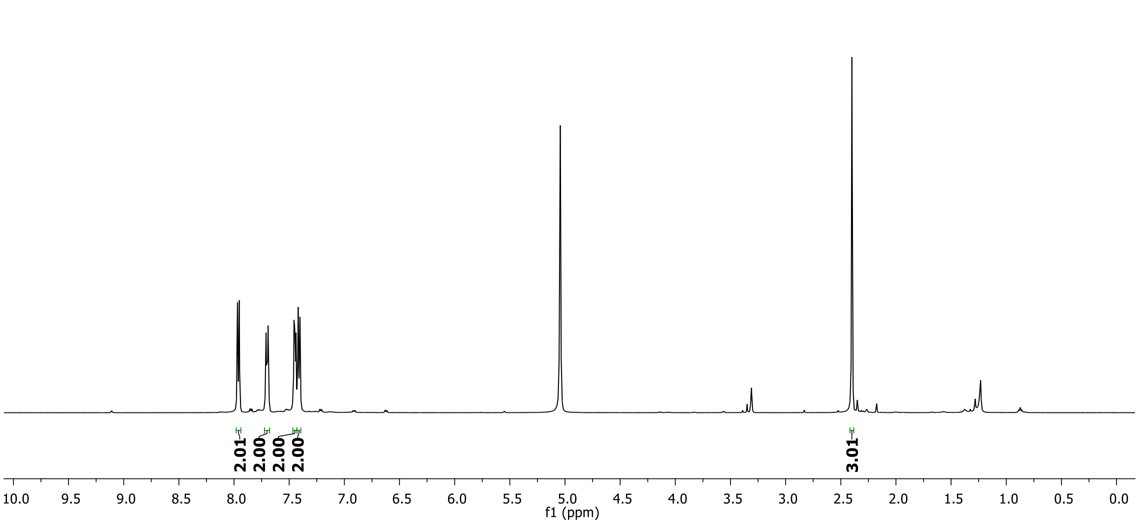

CD$_3$OD, 125MHz

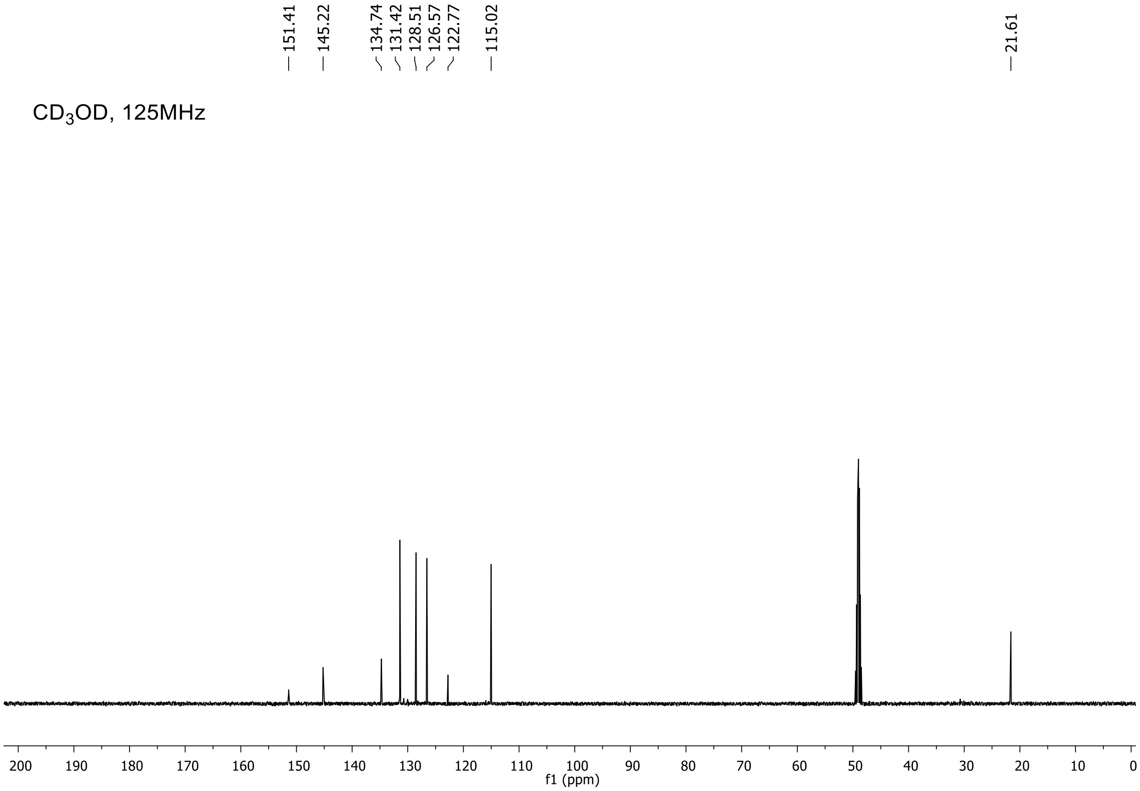

CD$_3$OD, 500MHz

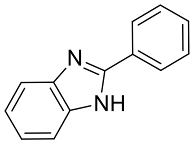

6

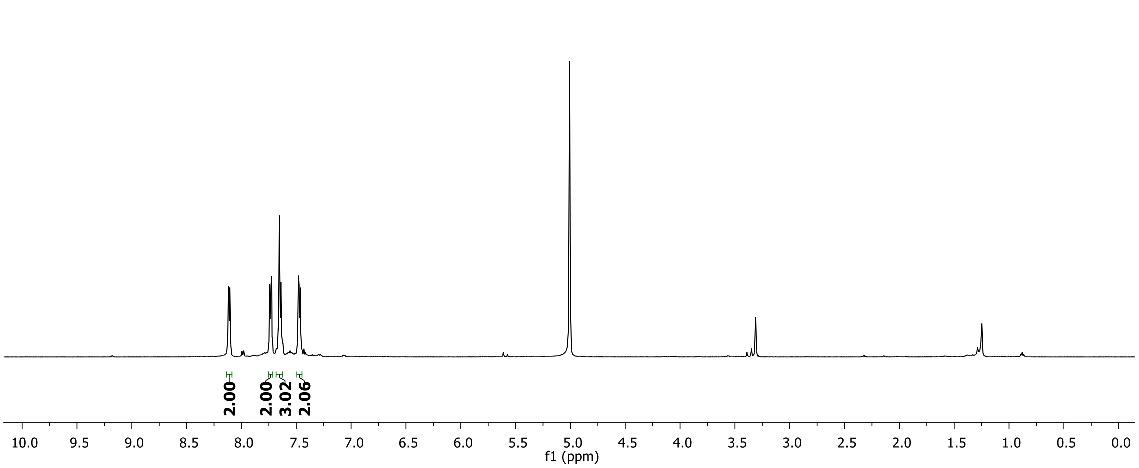

CD$_3$OD, 125MHz

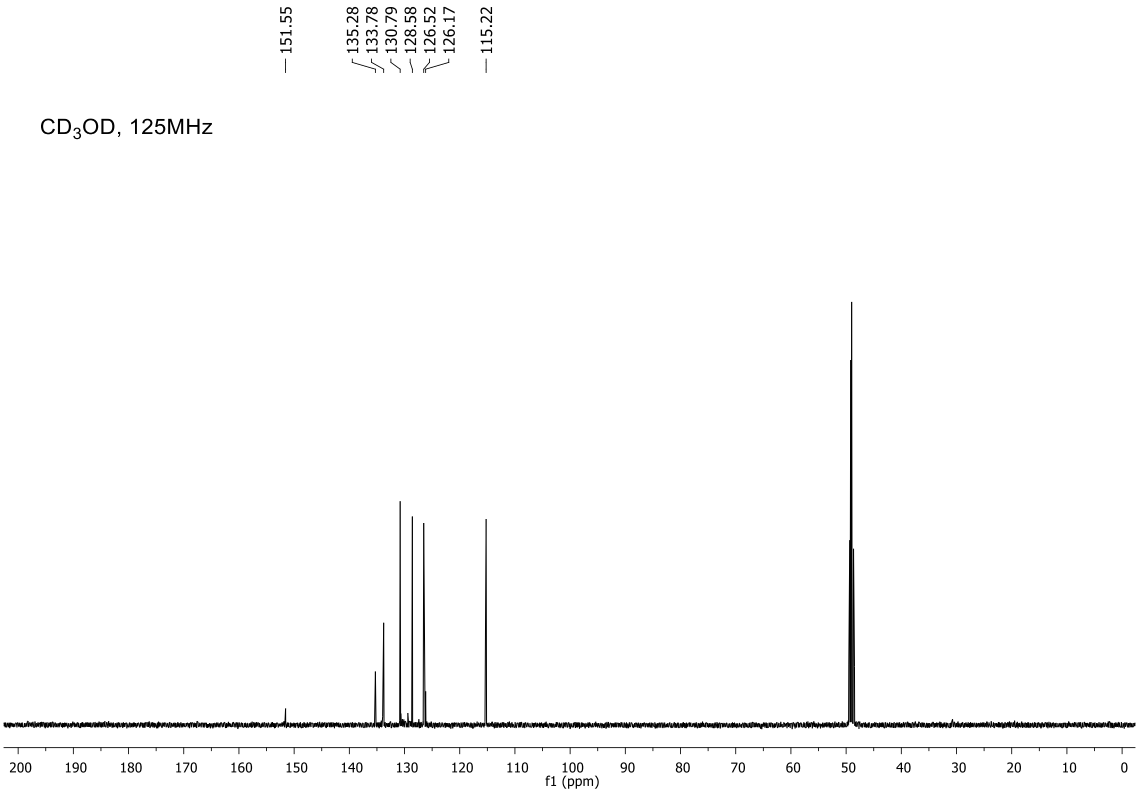

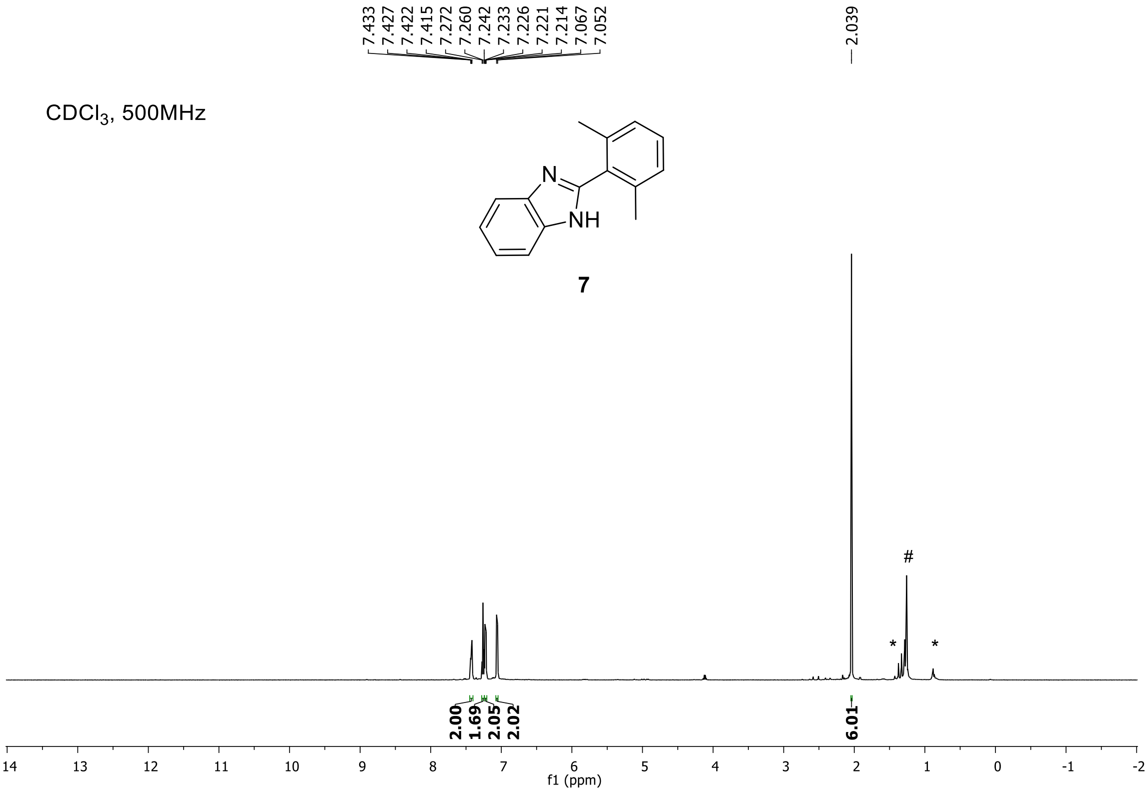

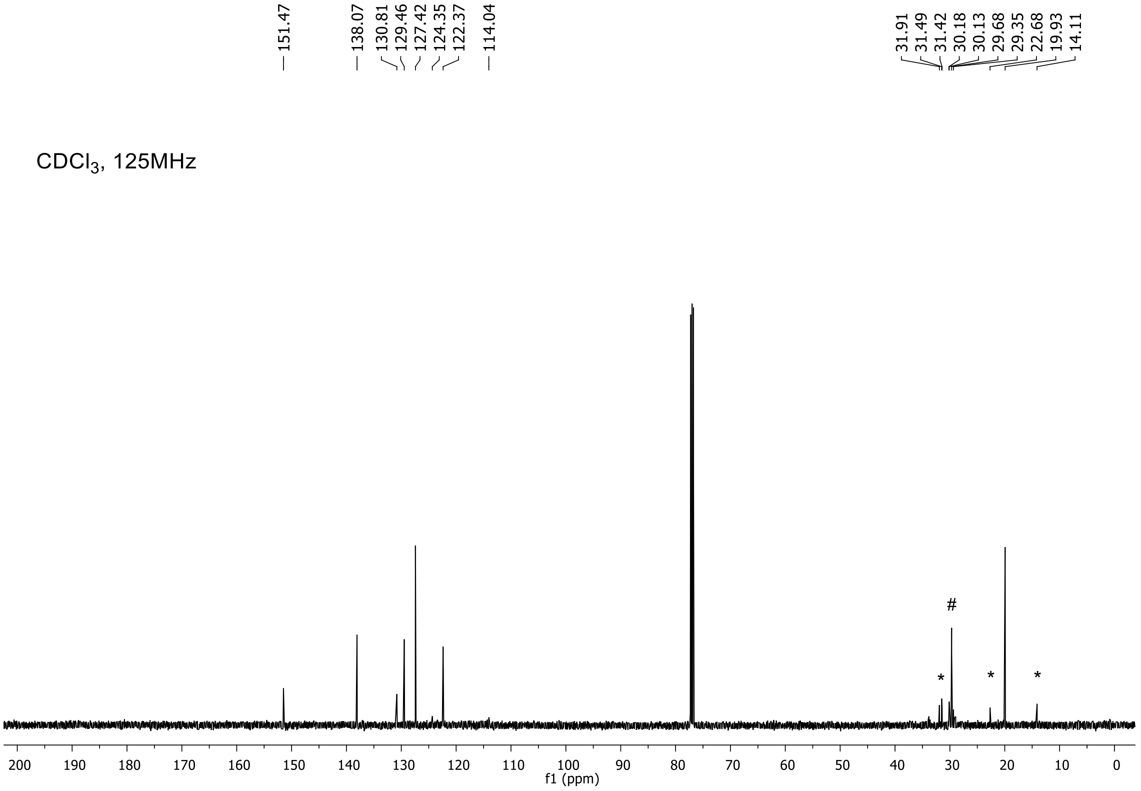

# = grease, * = Hexane

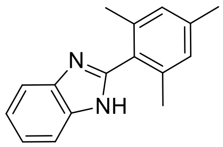

8

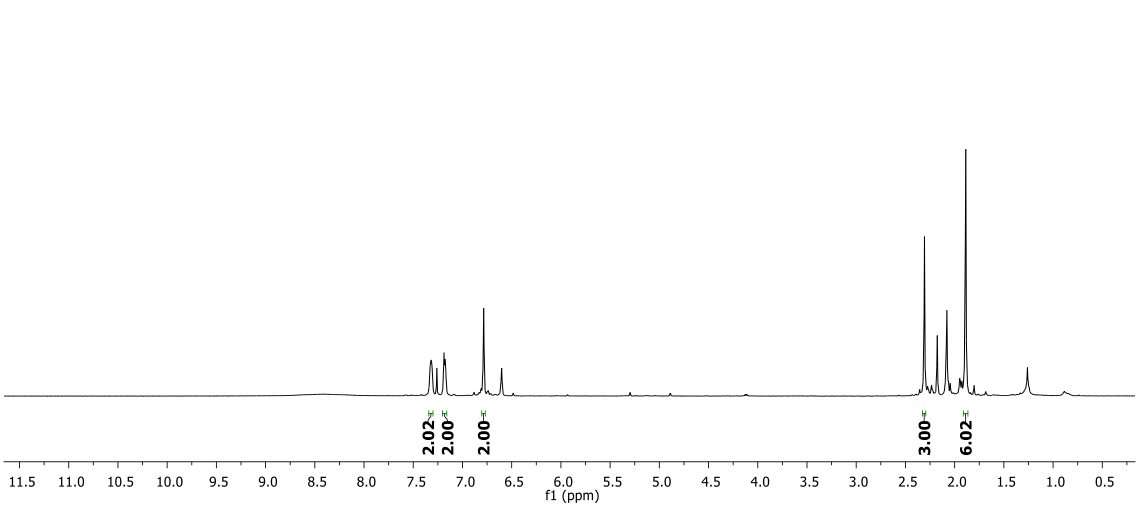
7.325
7.319
7.315
7.310
7.187
7.181
7.176
7.169
6.784
2.306
1.887
CDCl$_3$, 500MHz
2.02
2.00
2.00
3.00
6.02
11.5 11.0 10.5 10.0 9.5 9.0 8.5 8.0 7.5 7.0 6.5 6.0 5.5 5.0 4.5 4.0 3.5 3.0 2.5 2.0 1.5 1.0 0.5
f1 (ppm)

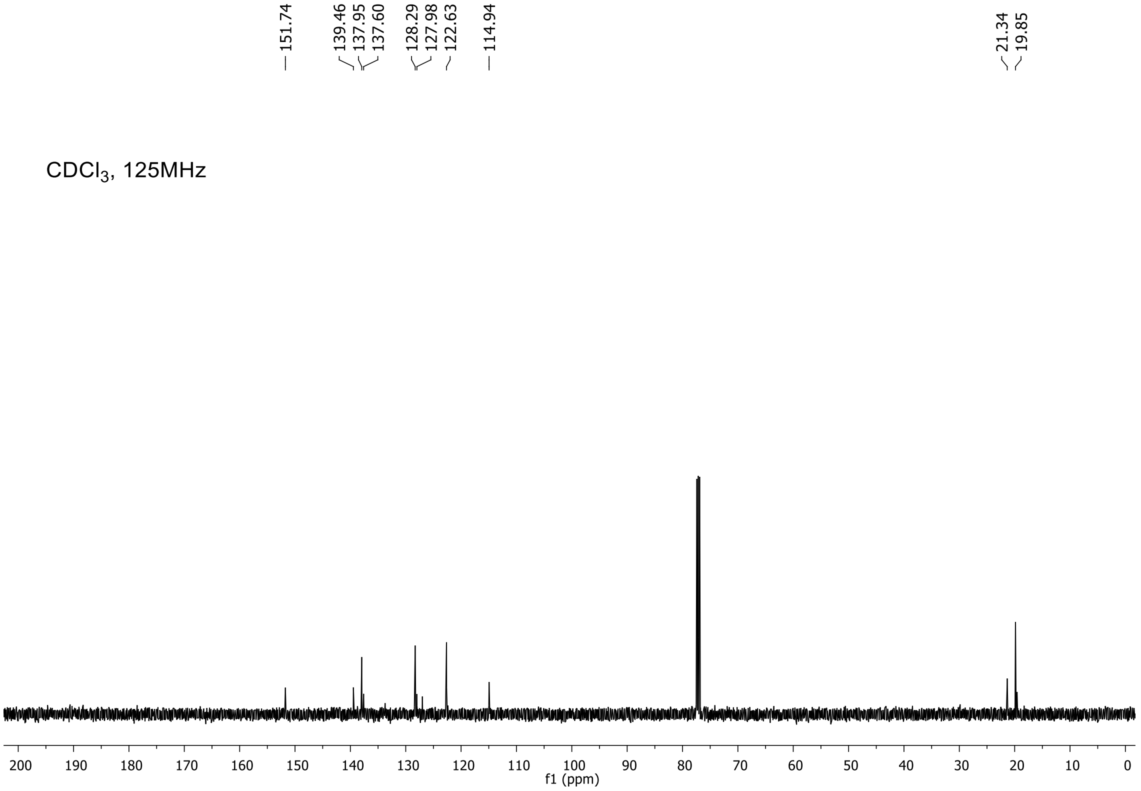
151.74
139.46
137.95
137.60
128.29
127.98
122.63
114.94
21.34
19.85
CDCl$_3$, 125MHz
200 190 180 170 160 150 140 130 120 110 100 90 80 70 60 50 40 30 20 10 0
f1 (ppm)

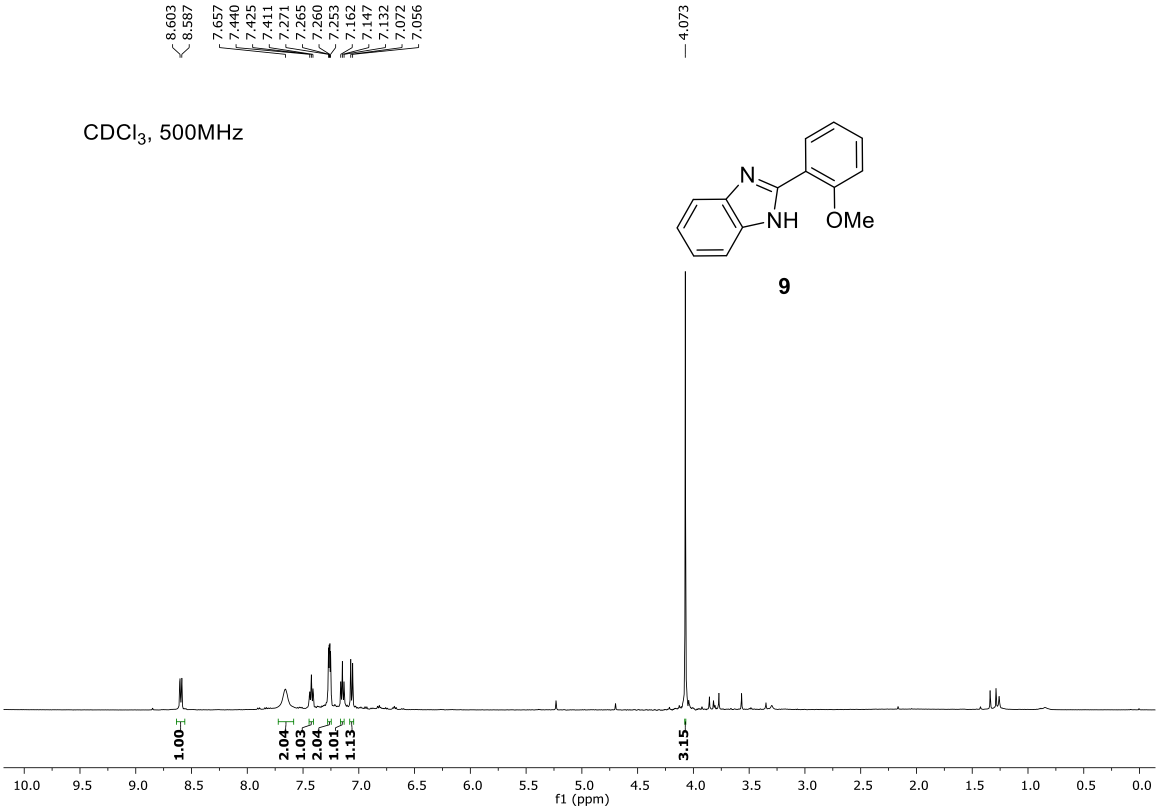
8.603
8.587
7.657
7.440
7.425
7.411
7.271
7.265
7.260
7.253
7.162
7.147
7.132
7.072
7.056
4.073
CDCl$_3$, 500MHz
N
NH
OMe
1.00
2.04
1.03
2.04
1.01
1.13
3.15
10.0 9.5 9.0 8.5 8.0 7.5 7.0 6.5 6.0 5.5 5.0 4.5 4.0 3.5 3.0 2.5 2.0 1.5 1.0 0.5 0.0
f1 (ppm)

9

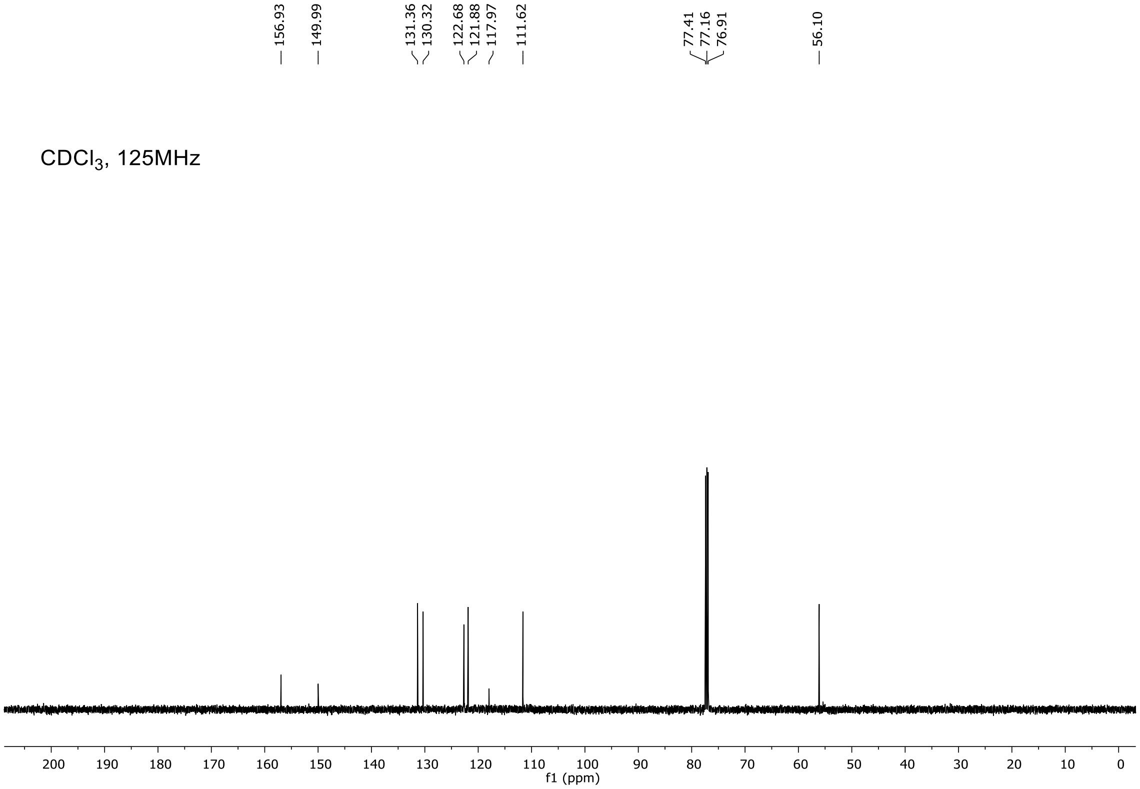
156.93
149.99
131.36
130.32
122.68
121.88
117.97
111.62
77.41
77.16
76.91
56.10
CDCl$_3$, 125MHz
200 190 180 170 160 150 140 130 120 110 100 90 80 70 60 50 40 30 20 10 0
f1 (ppm)

CDCl$_3$, 500MHz

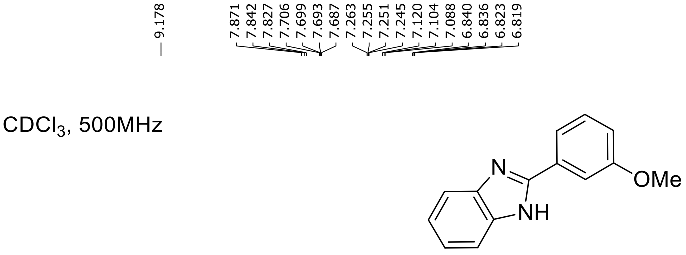

10

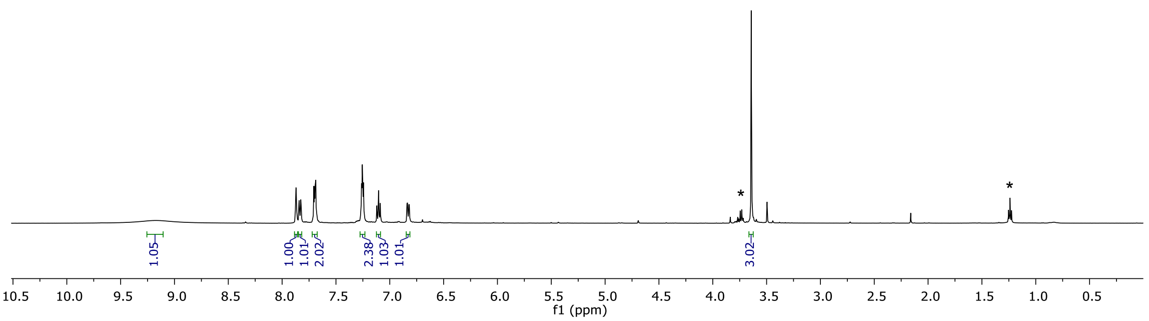

CDCl$_3$, 125MHz

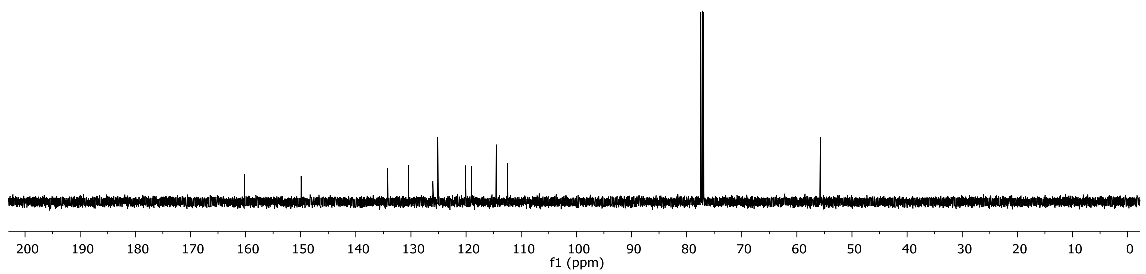

* = Ethanol

CD$_3$OD, 500MHz

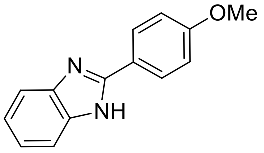

11

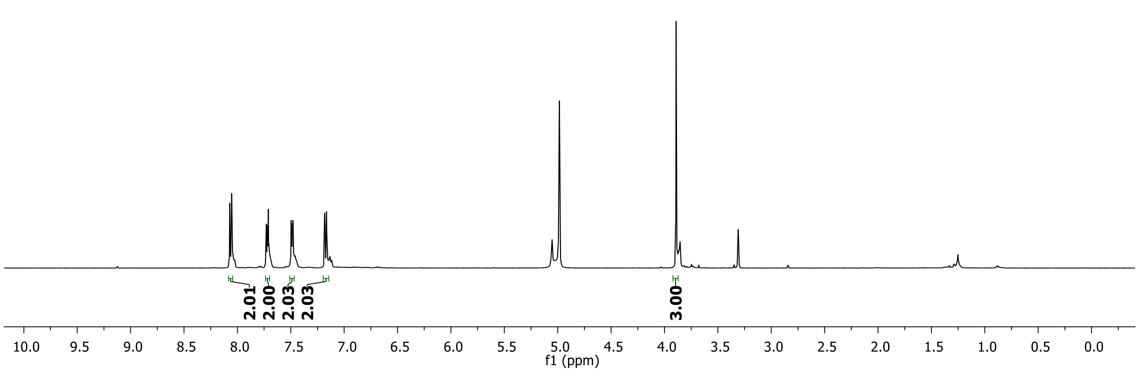

CD$_3$OD, 125MHz

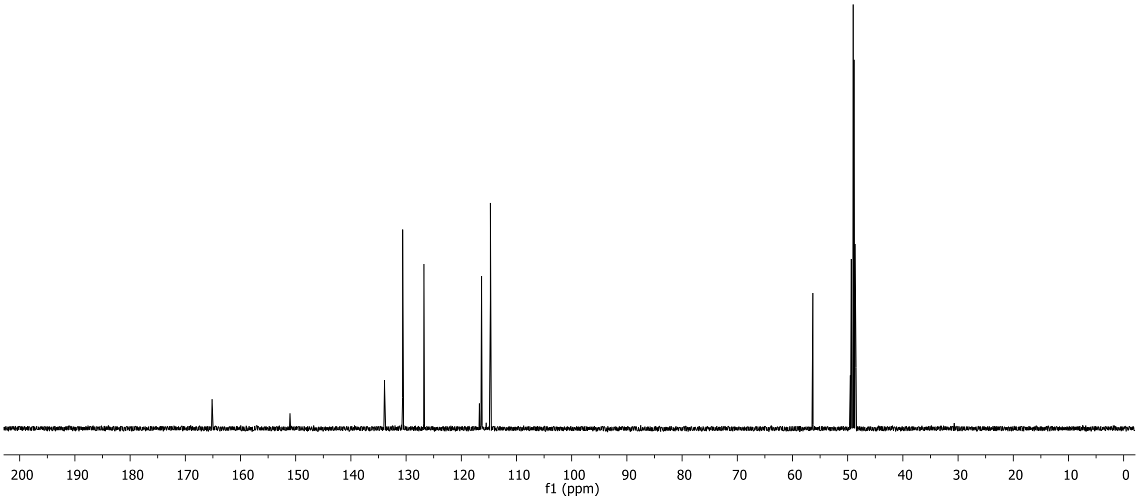

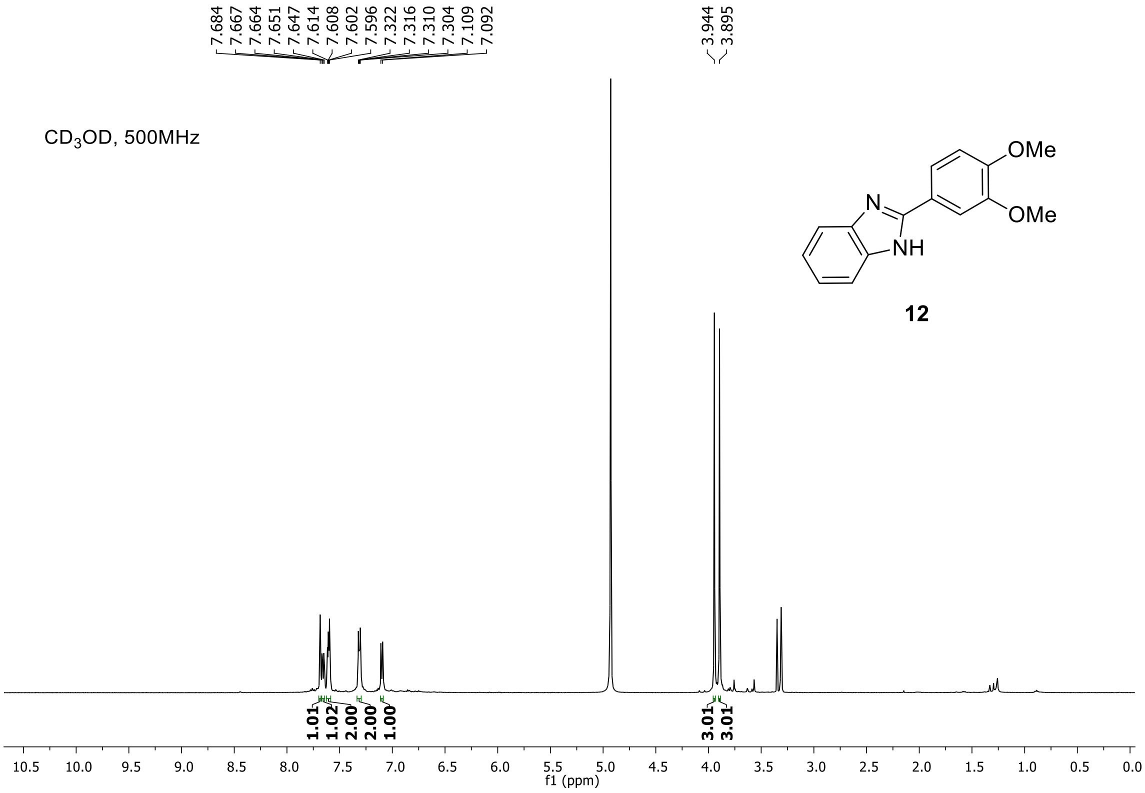
7.684
7.667
7.664
7.651
7.647
7.614
7.608
7.602
7.596
7.322
7.316
7.310
7.304
7.109
7.092
3.944
3.895
CD$_3$OD, 500MHz
OMe
OMe
N
NH
12
1.01
1.02
2.00
2.00
1.00
3.01
3.01
10.5 10.0 9.5 9.0 8.5 8.0 7.5 7.0 6.5 6.0 5.5 5.0 4.5 4.0 3.5 3.0 2.5 2.0 1.5 1.0 0.5 0.0
f1 (ppm)

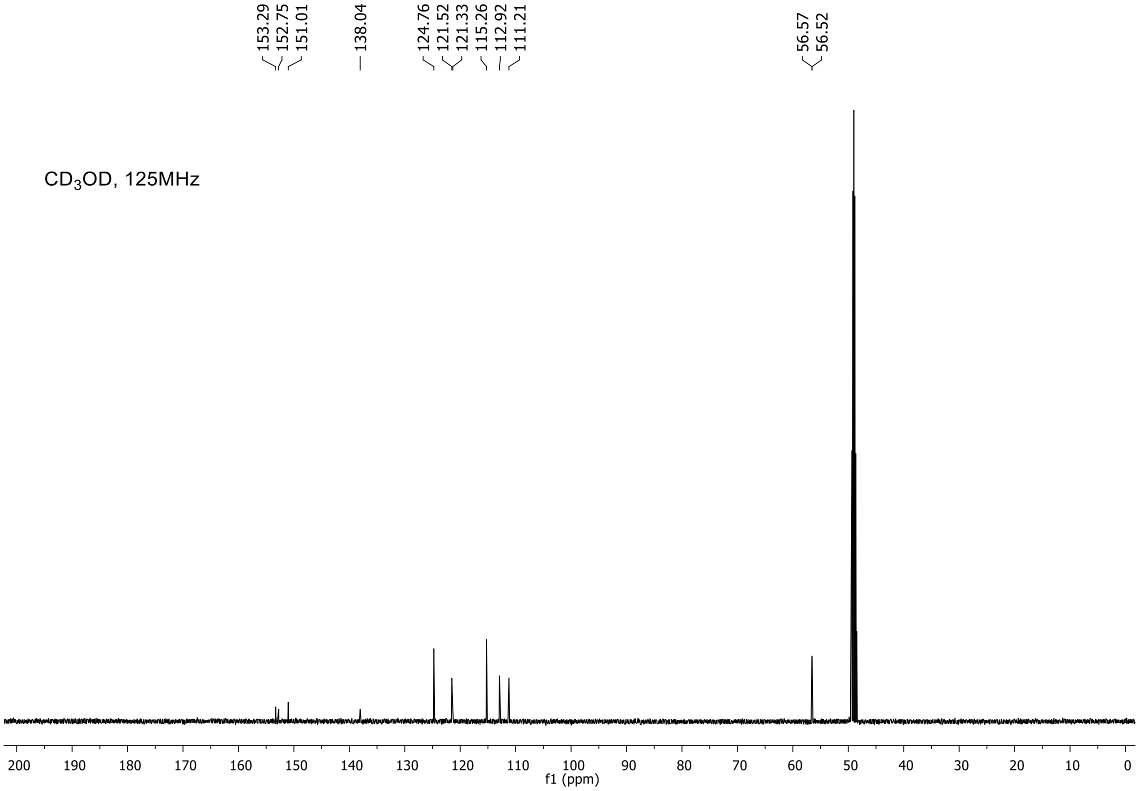
153.29
152.75
151.01
138.04
124.76
121.52
121.33
115.26
112.92
111.21
56.57
56.52
CD$_3$OD, 125MHz
200 190 180 170 160 150 140 130 120 110 100 90 80 70 60 50 40 30 20 10 0
f1 (ppm)

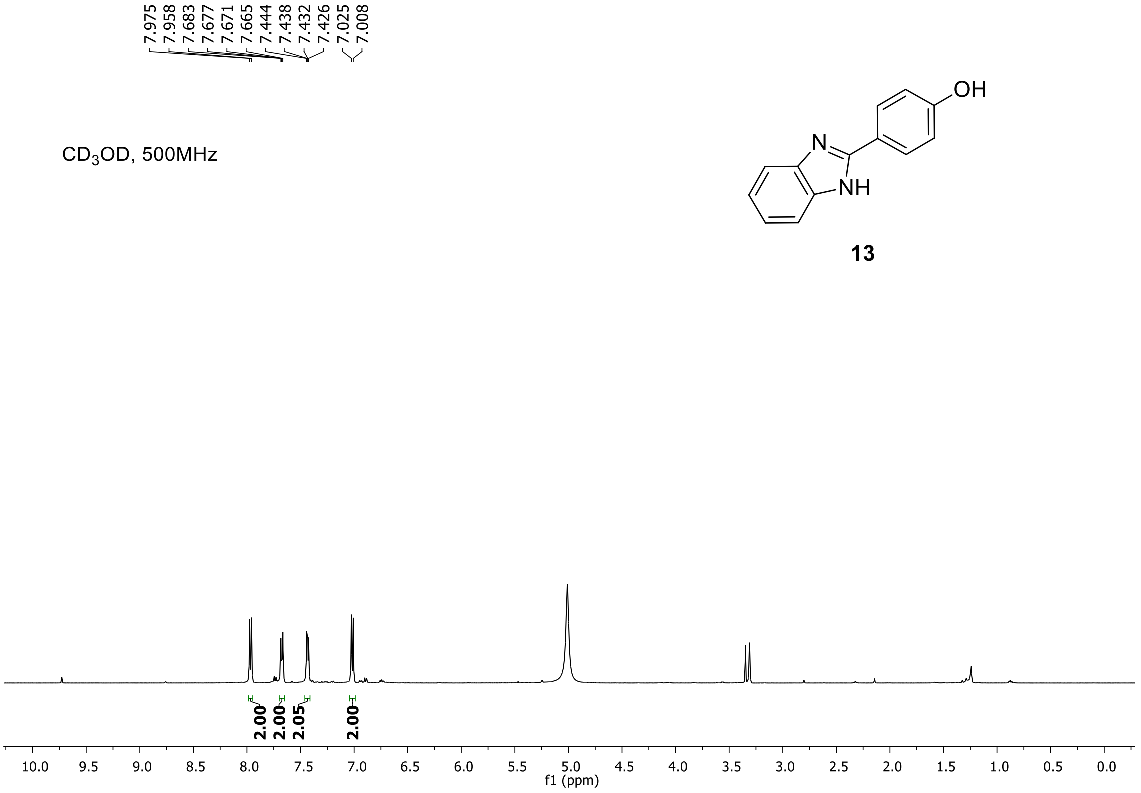
7.975
7.958
7.683
7.677
7.671
7.665
7.444
7.438
7.432
7.426
7.025
7.008
CD3OD, 500MHz
2.00
2.00
2.05
2.00
10.0 9.5 9.0 8.5 8.0 7.5 7.0 6.5 6.0 5.5 5.0 4.5 4.0 3.5 3.0 2.5 2.0 1.5 1.0 0.5 0.0
f1 (ppm)
OH
N
NH

13

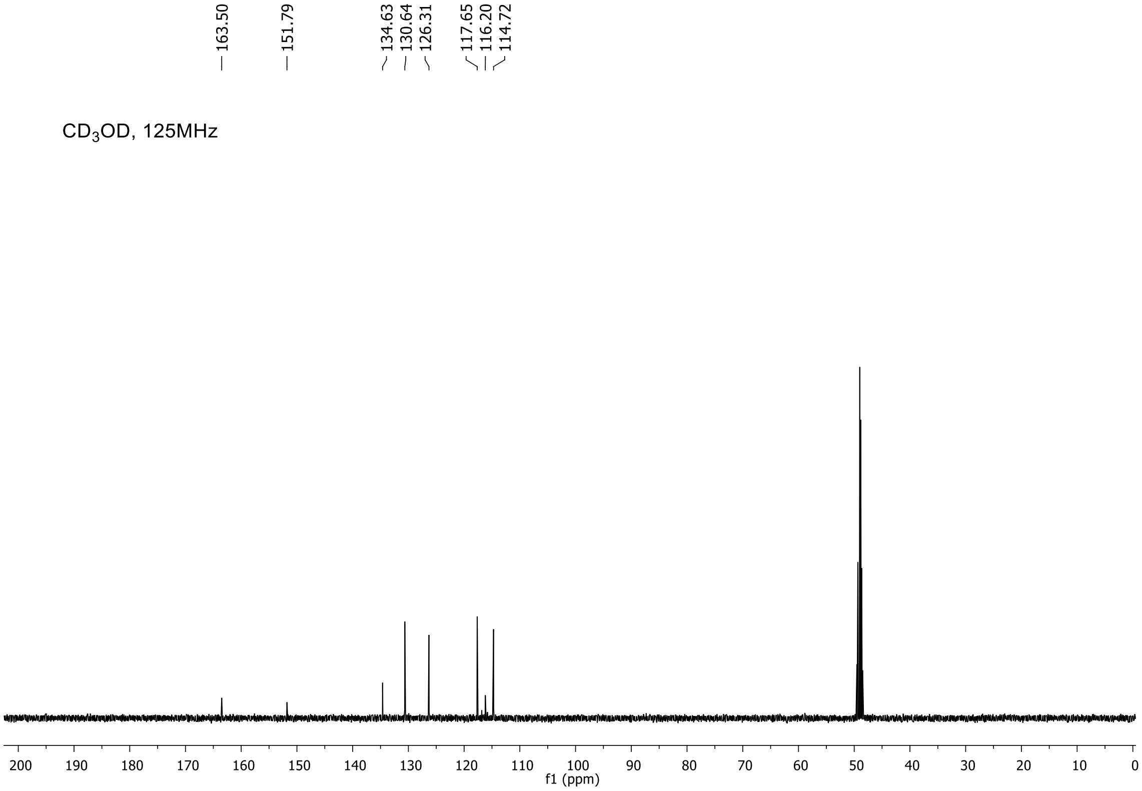
163.50
151.79
134.63
130.64
126.31
117.65
116.20
114.72
CD3OD, 125MHz
200 190 180 170 160 150 140 130 120 110 100 90 80 70 60 50 40 30 20 10 0
f1 (ppm)

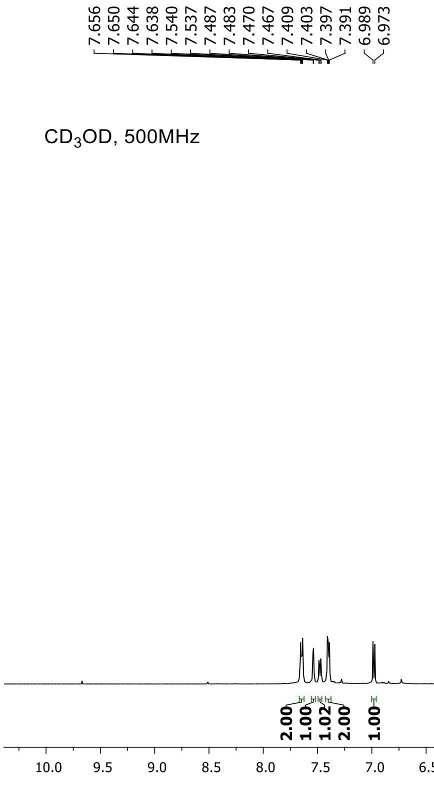
7.656
7.650
7.644
7.638
7.540
7.537
7.487
7.483
7.470
7.467
7.409
7.403
7.397
7.391
6.989
6.973
CD3OD, 500MHz
2.00
1.00
1.02
2.00
1.00
10.0 9.5 9.0 8.5 8.0 7.5 7.0 6.5 6.0 5.5 5.0 4.5 4.0 3.5 3.0 2.5 2.0 1.5 1.0 0.5 0.0
f1 (ppm)

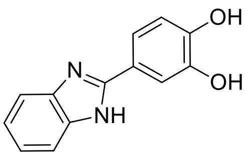
OH
OH
N
NH

14

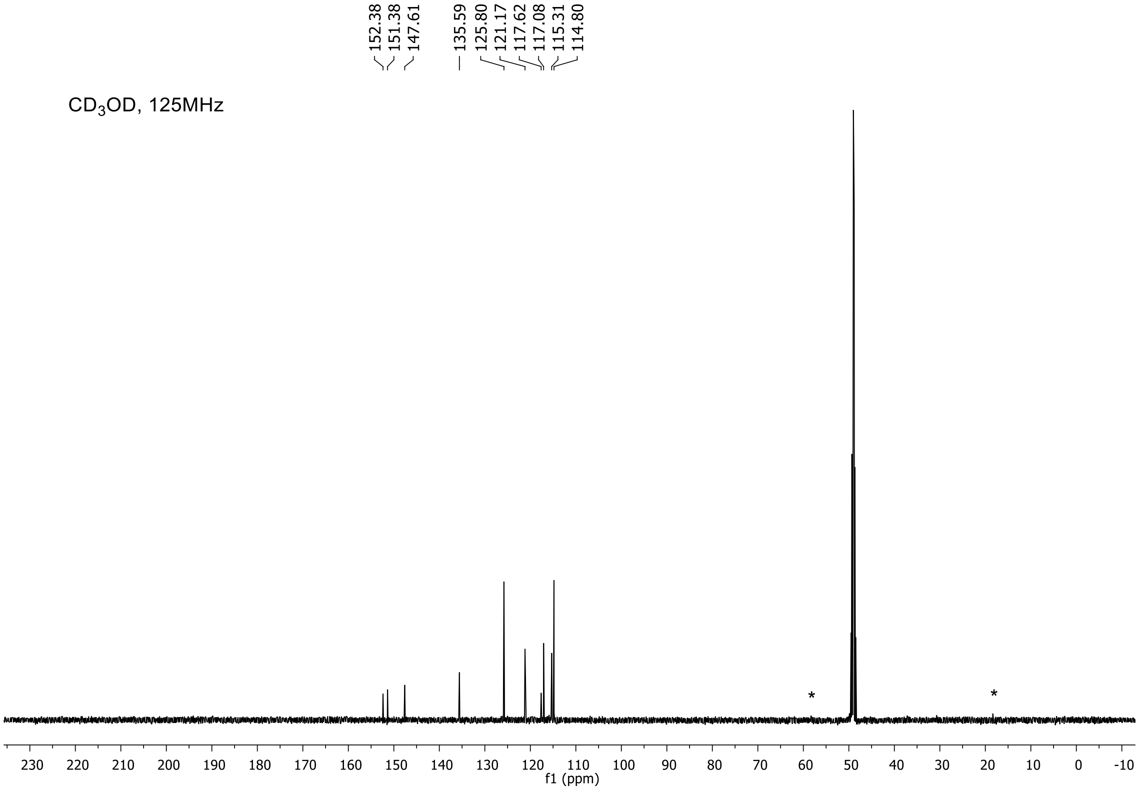
152.38
151.38
147.61
135.59
125.80
121.17
117.62
117.08
115.31
114.80
CD3OD, 125MHz
230 220 210 200 190 180 170 160 150 140 130 120 110 100 90 80 70 60 50 40 30 20 10 0 -10
f1 (ppm)

* = ethanol

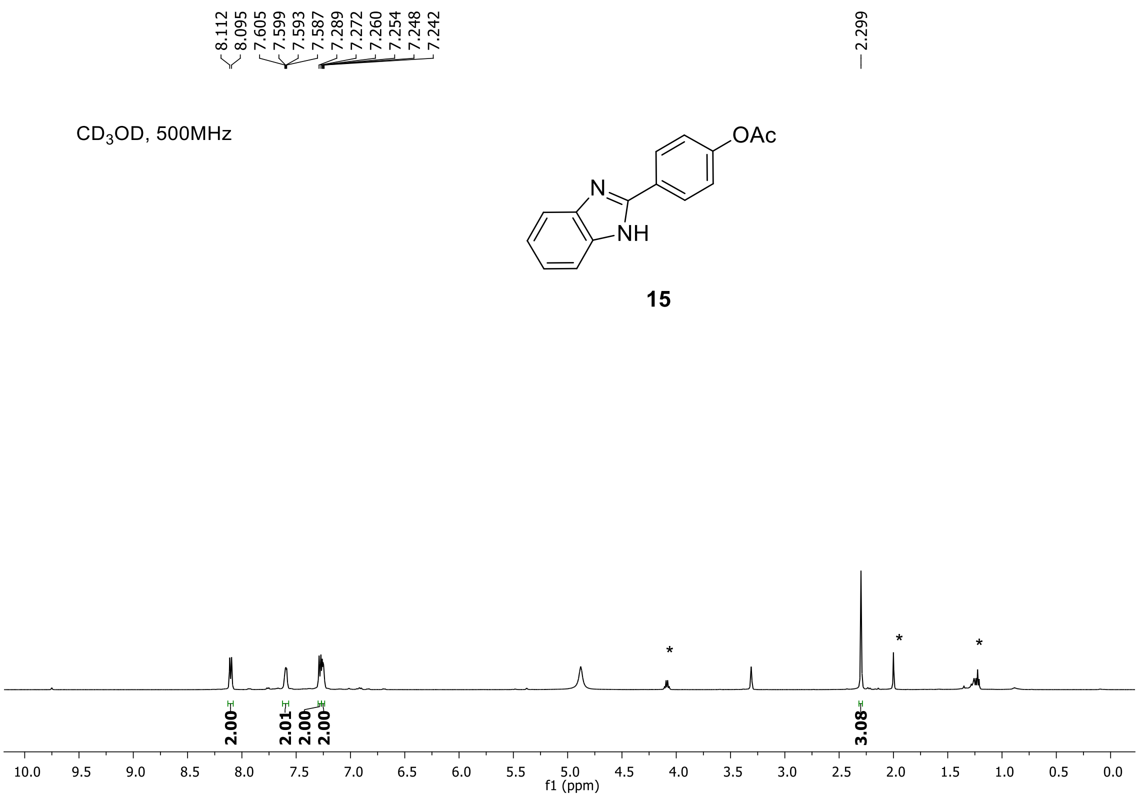

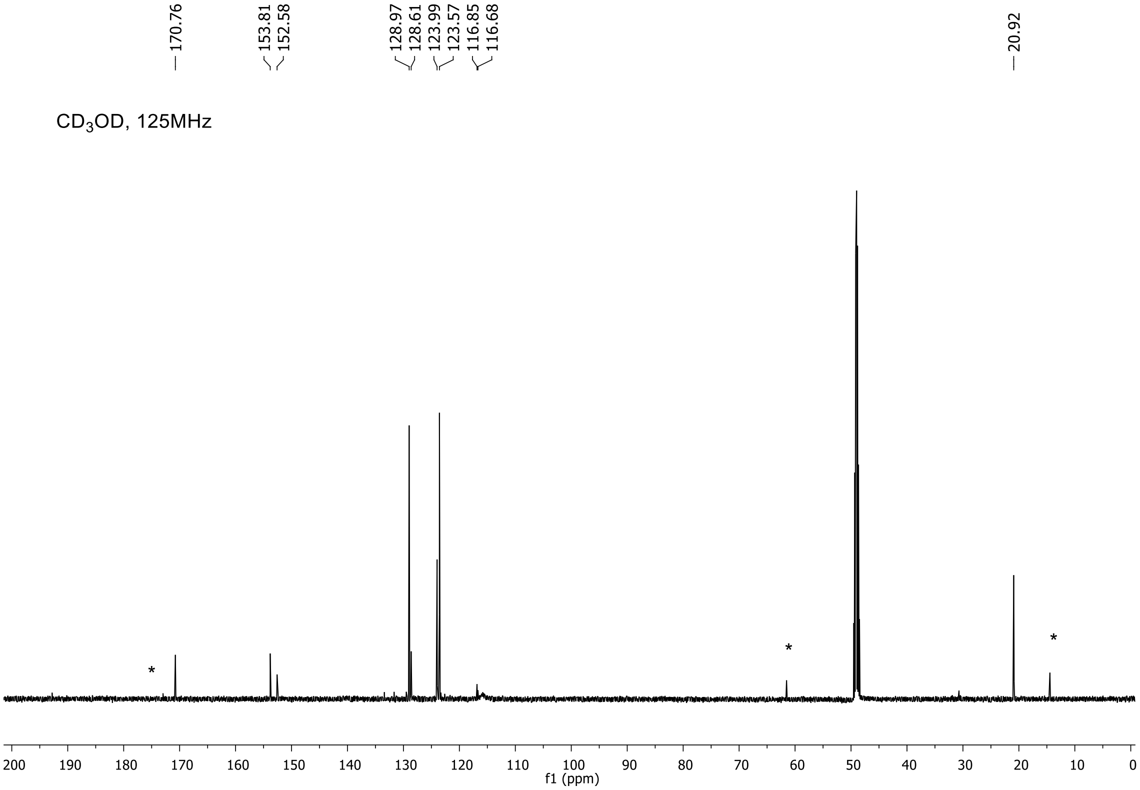

* = ethyl acetate

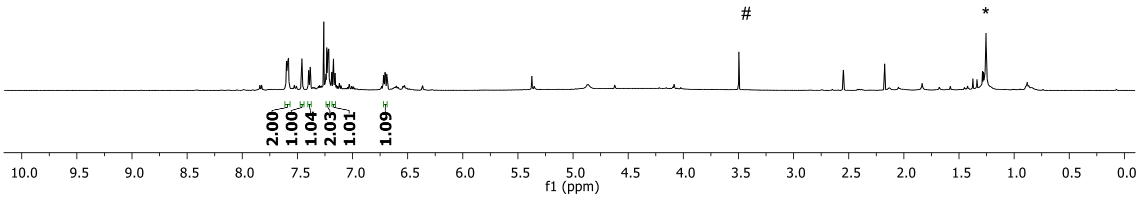

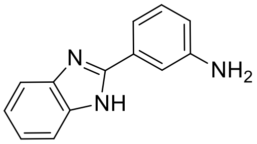

16

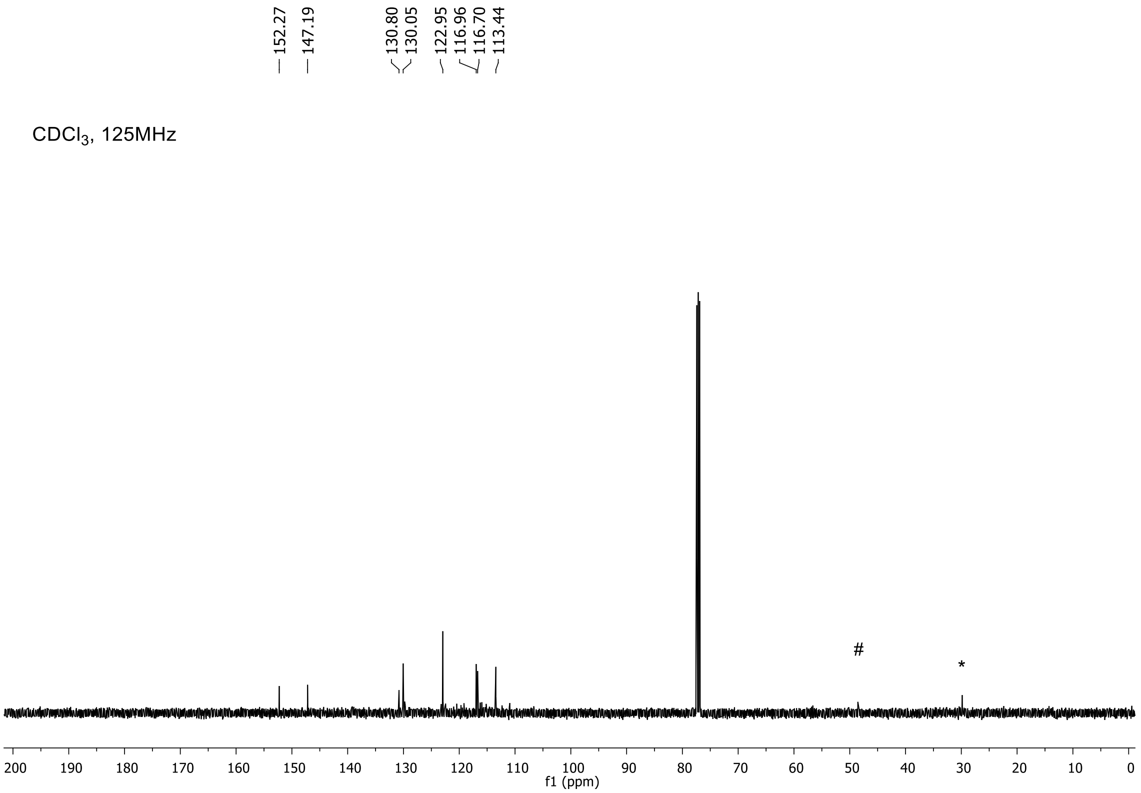

* = grease, # = methanol

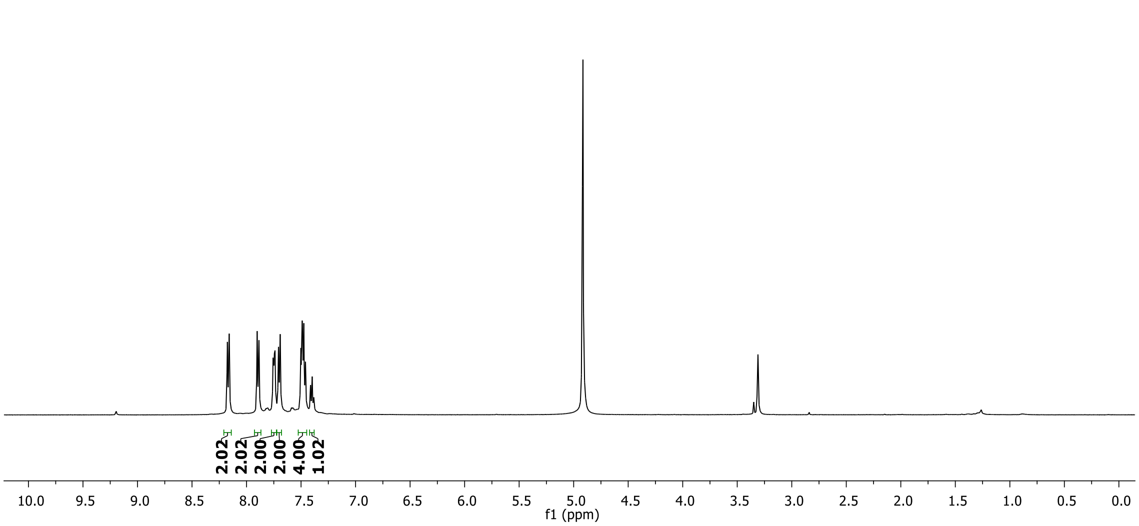
8.176
8.159
7.903
7.886
7.757
7.751
7.745
7.739
7.707
7.691
7.502
7.495
7.490
7.483
7.475
7.459
7.413
7.398
7.384
CD3OD, 500MHz
2.02
2.02
2.00
2.00
4.00
1.02
f1 (ppm)

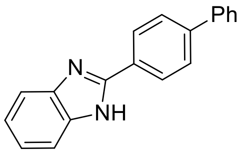
N
NH
Ph

17

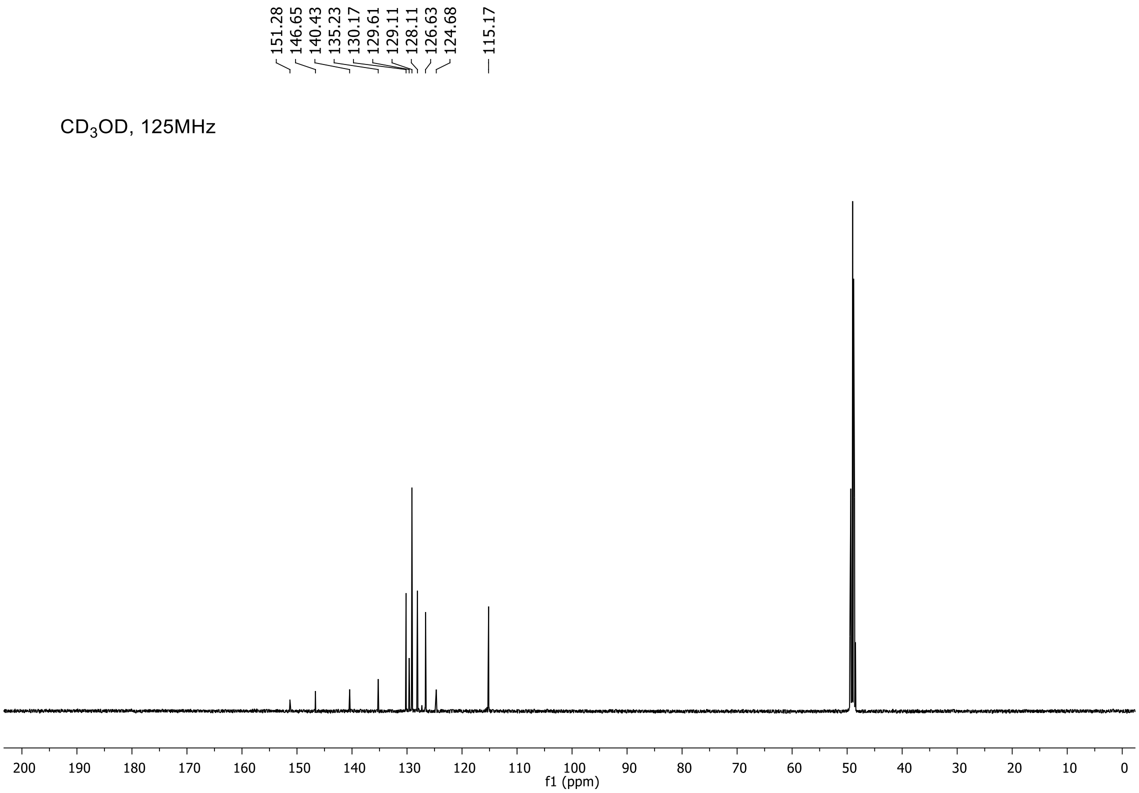
151.28
146.65
140.43
135.23
130.17
129.61
129.11
128.11
126.63
124.68
115.17
CD3OD, 125MHz
f1 (ppm)

CD$_3$OD, 500MHz

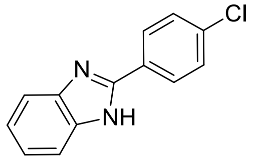

18

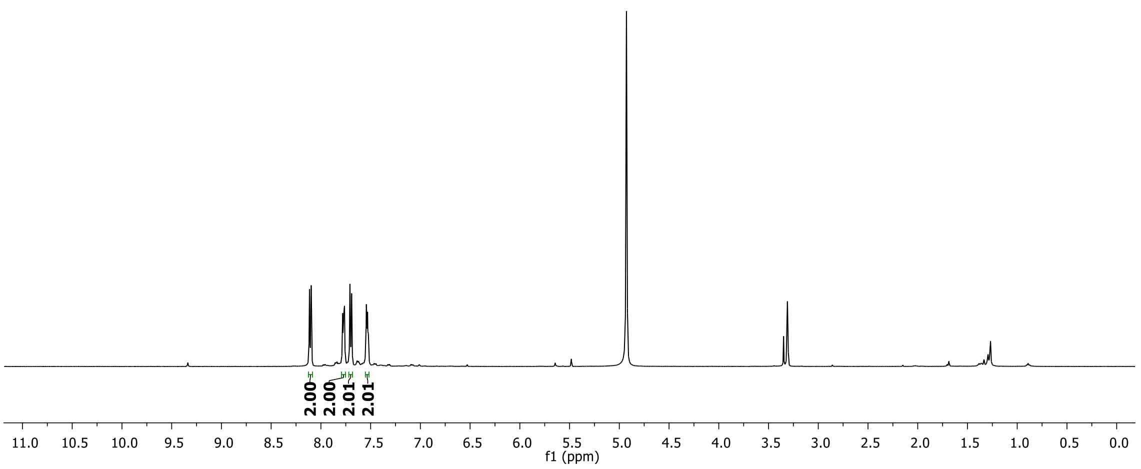

CD$_3$OD, 125MHz

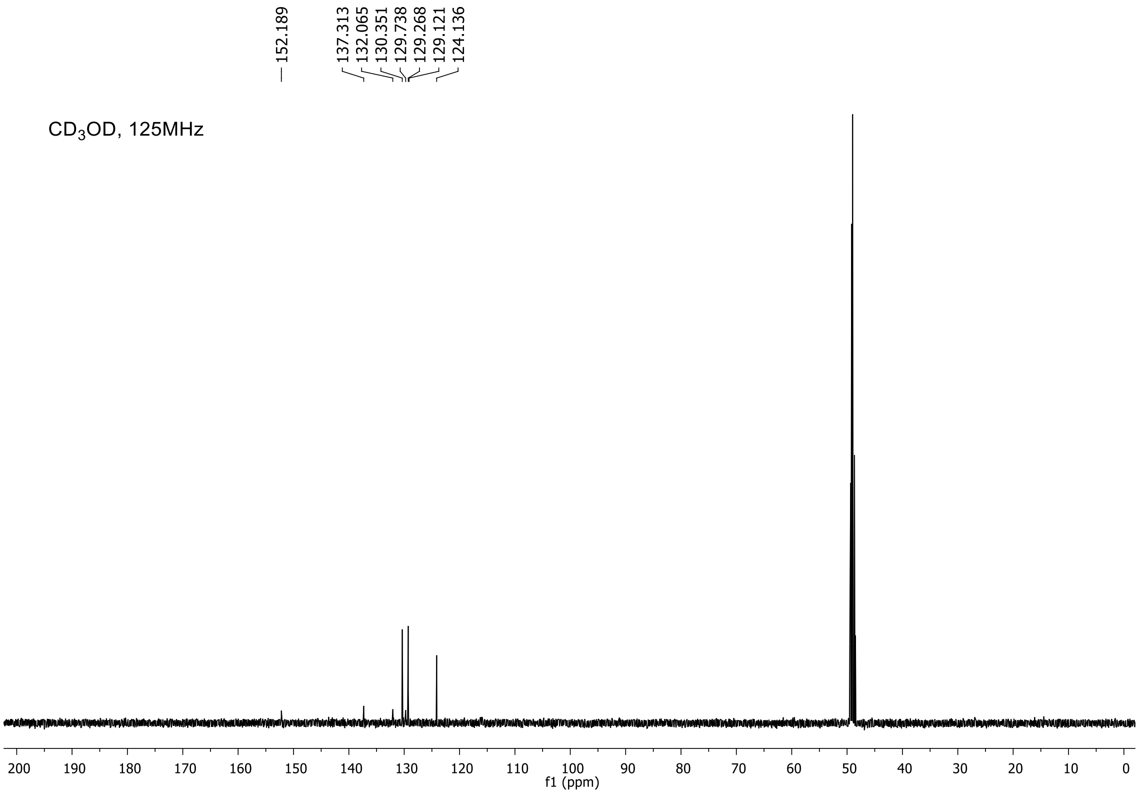

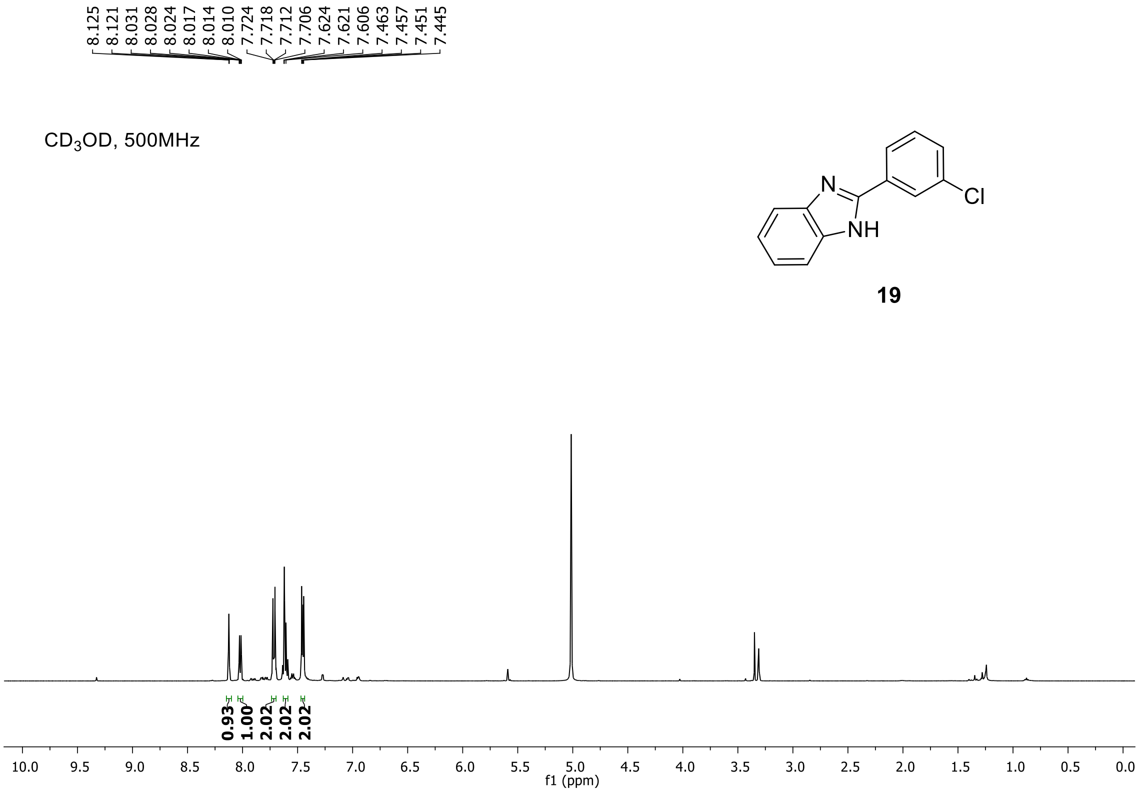
8.125
8.121
8.031
8.028
8.024
8.017
8.014
8.010
7.724
7.718
7.712
7.706
7.624
7.621
7.606
7.463
7.457
7.451
7.445
CD3OD, 500MHz
0.93
1.00
2.02
2.02
2.02
10.0 9.5 9.0 8.5 8.0 7.5 7.0 6.5 6.0 5.5 5.0 4.5 4.0 3.5 3.0 2.5 2.0 1.5 1.0 0.5 0.0
f1 (ppm)
N
NH
Cl

19

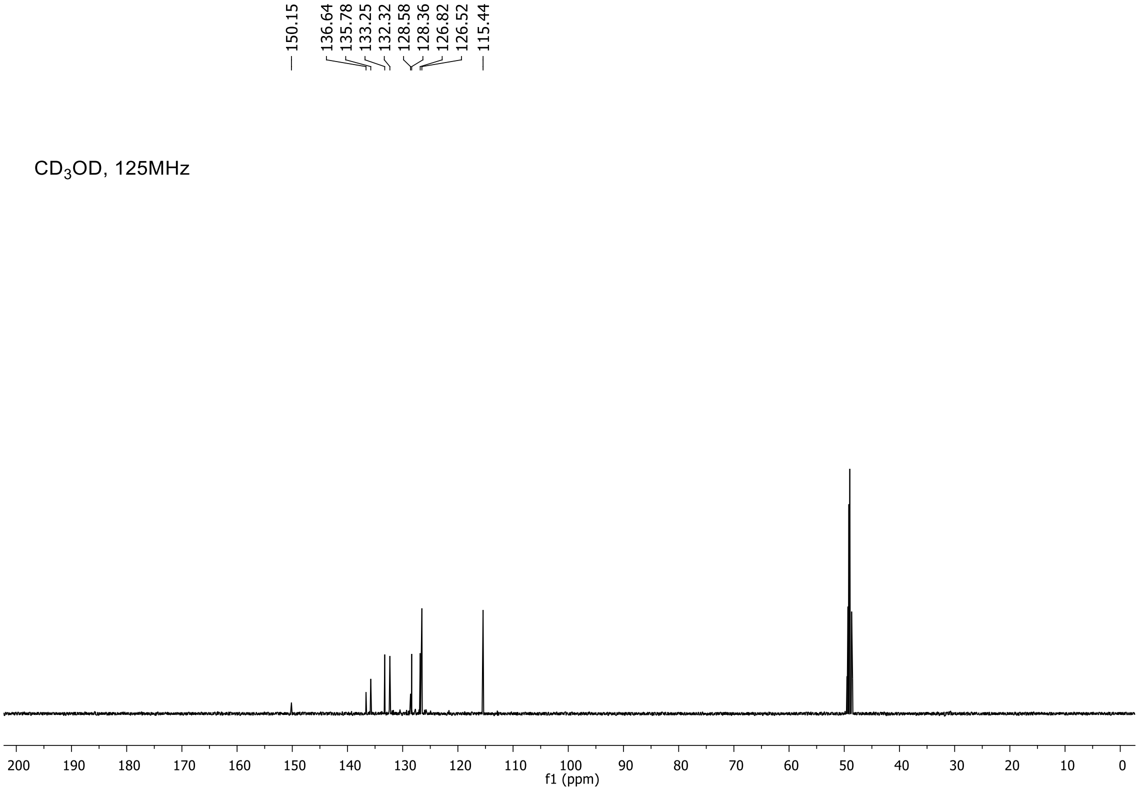
150.15
136.64
135.78
133.25
132.32
128.58
128.36
126.82
126.52
115.44
CD3OD, 125MHz
200 190 180 170 160 150 140 130 120 110 100 90 80 70 60 50 40 30 20 10 0
f1 (ppm)

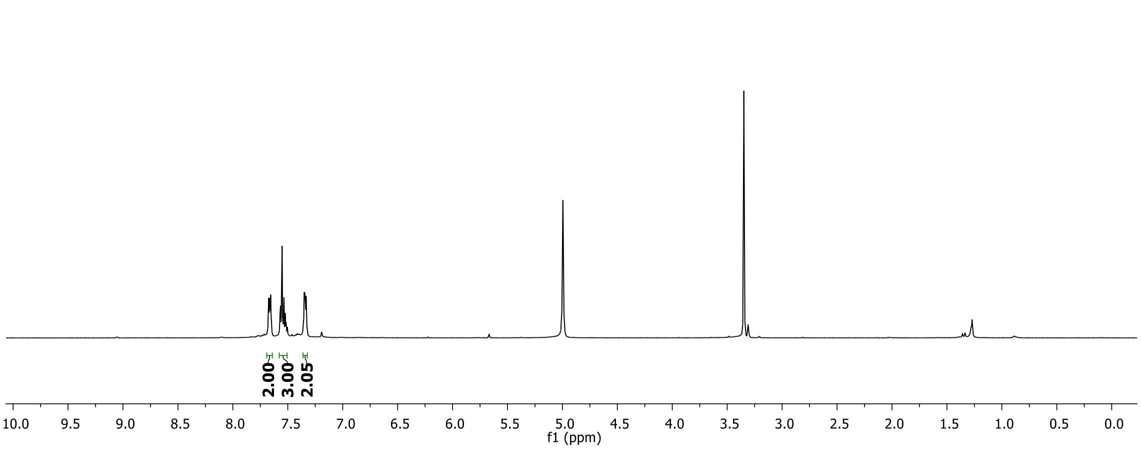
7.674
7.667
7.661
7.655
7.570
7.565
7.551
7.535
7.522
7.515
7.351
7.344
7.339
7.333
CD$_3$OD, 500MHz
2.00
3.00
2.05
f1 (ppm)

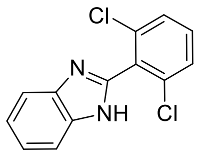
Cl
N
NH
Cl

20

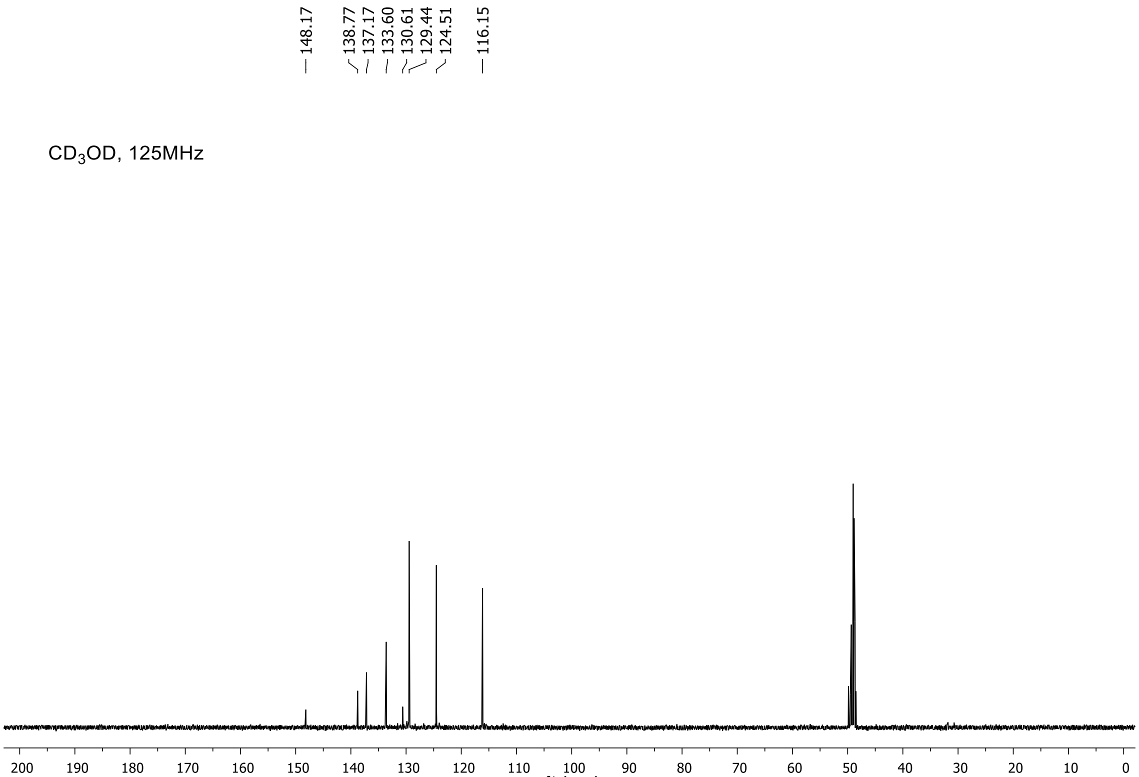
148.17
138.77
137.17
133.60
130.61
129.44
124.51
116.15
CD$_3$OD, 125MHz
f1 (ppm)

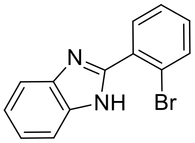
N
NH
Br

21

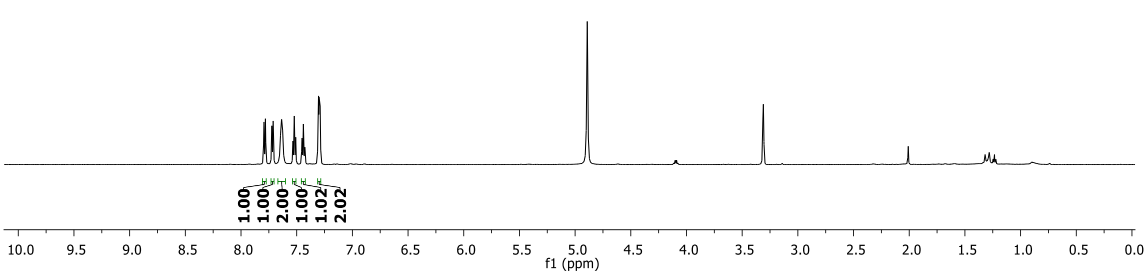
7.794
7.781
7.724
7.711
7.635
7.534
7.522
7.509
7.453
7.450
7.440
7.427
7.304
7.299
7.294
7.289
CD3OD, 600MHz
1.00
1.00
2.00
1.00
1.02
2.02
10.0 9.5 9.0 8.5 8.0 7.5 7.0 6.5 6.0 5.5 5.0 4.5 4.0 3.5 3.0 2.5 2.0 1.5 1.0 0.5 0.0
f1 (ppm)

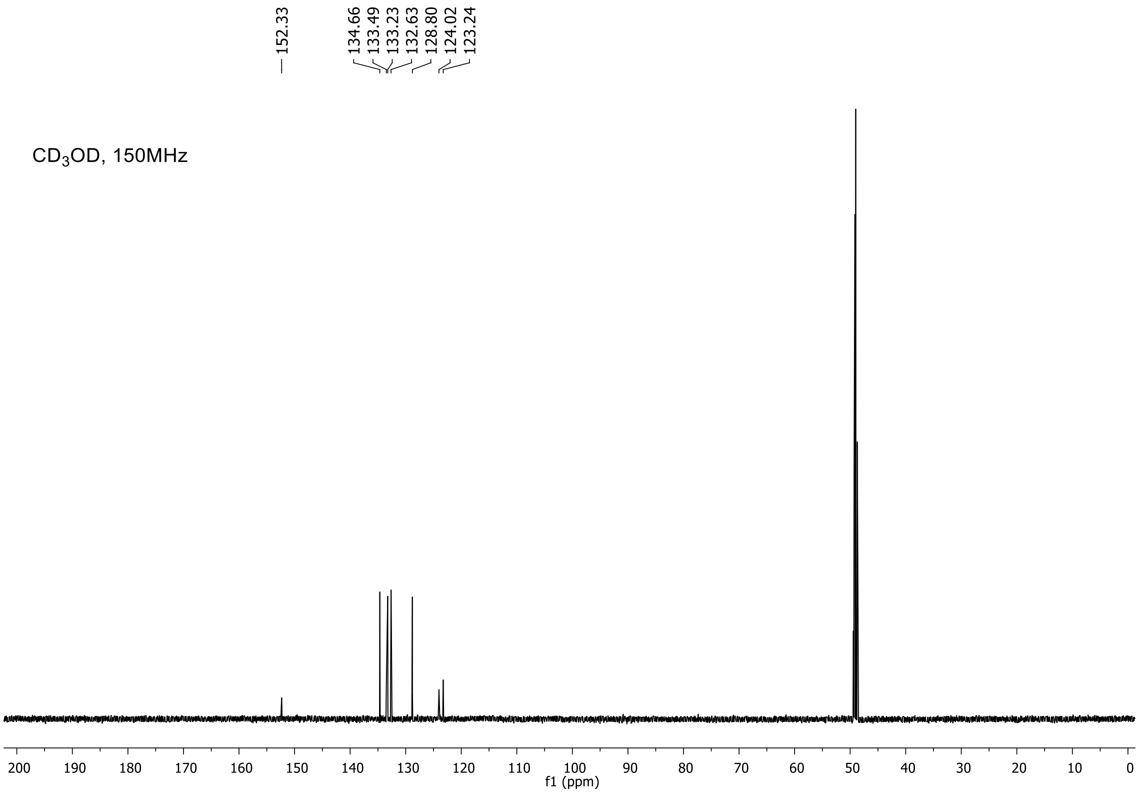
152.33
134.66
133.49
133.23
132.63
128.80
124.02
123.24
CD3OD, 150MHz
200 190 180 170 160 150 140 130 120 110 100 90 80 70 60 50 40 30 20 10 0
f1 (ppm)

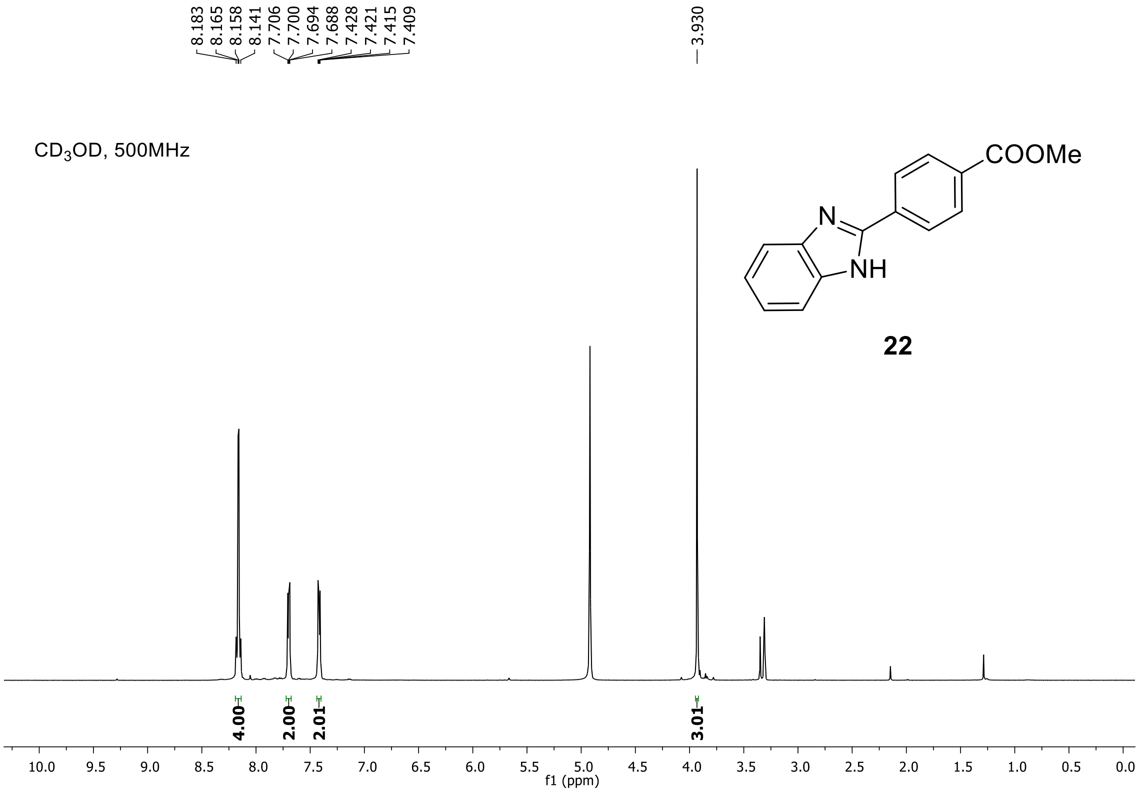
8.183
8.165
8.158
8.141
7.706
7.700
7.694
7.688
7.428
7.421
7.415
7.409
3.930
CD3OD, 500MHz
COOMe
N
NH
4.00
2.00
2.01
3.01
10.0 9.5 9.0 8.5 8.0 7.5 7.0 6.5 6.0 5.5 5.0 4.5 4.0 3.5 3.0 2.5 2.0 1.5 1.0 0.5 0.0
f1 (ppm)

22

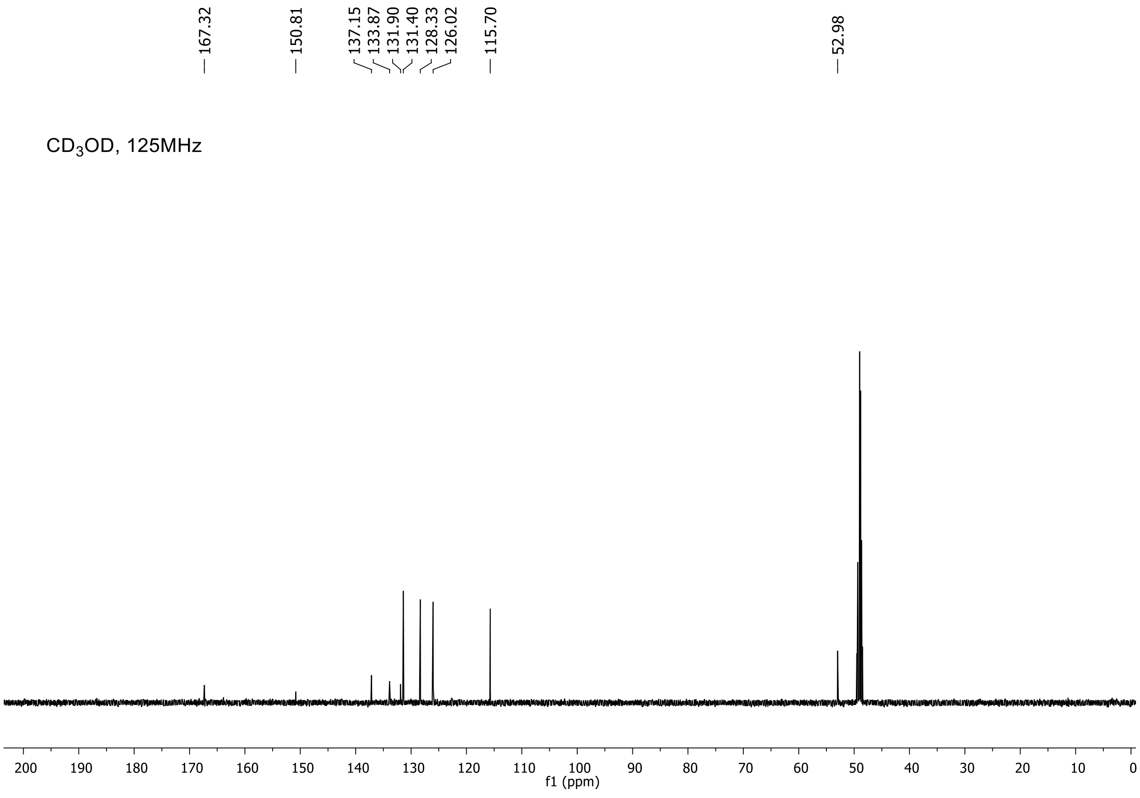
167.32
150.81
137.15
133.87
131.90
131.40
128.33
126.02
115.70
52.98
CD3OD, 125MHz
200 190 180 170 160 150 140 130 120 110 100 90 80 70 60 50 40 30 20 10 0
f1 (ppm)

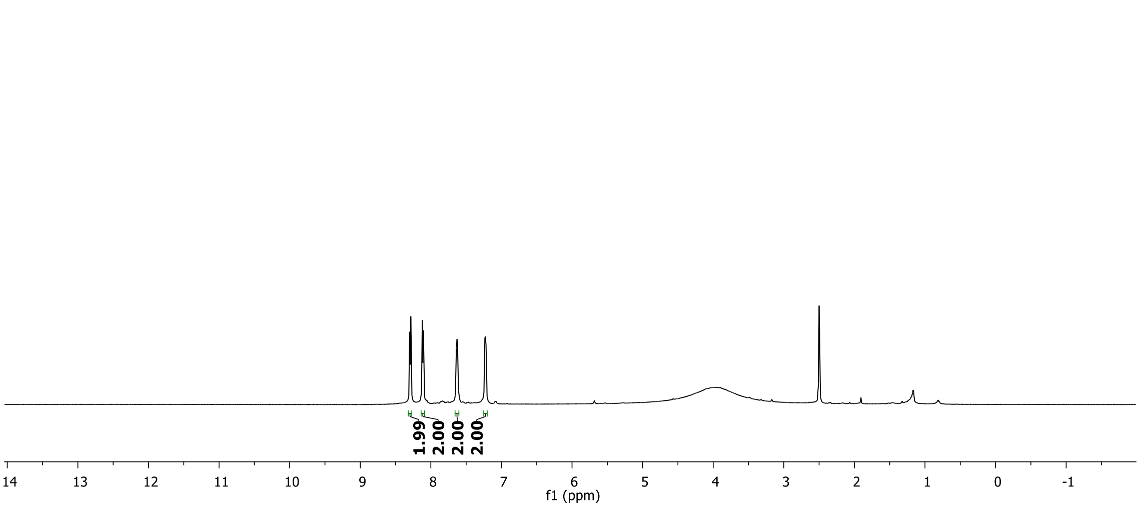
8.299
8.284
8.121
8.105
7.630
7.235
7.229
7.224
2.500
DMSO-d_6, 500MHz
1.99
2.00
2.00
2.00
14 13 12 11 10 9 8 7 6 5 4 3 2 1 0 -1
f1 (ppm)

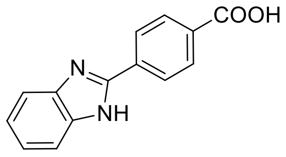
COOH
N
NH

23

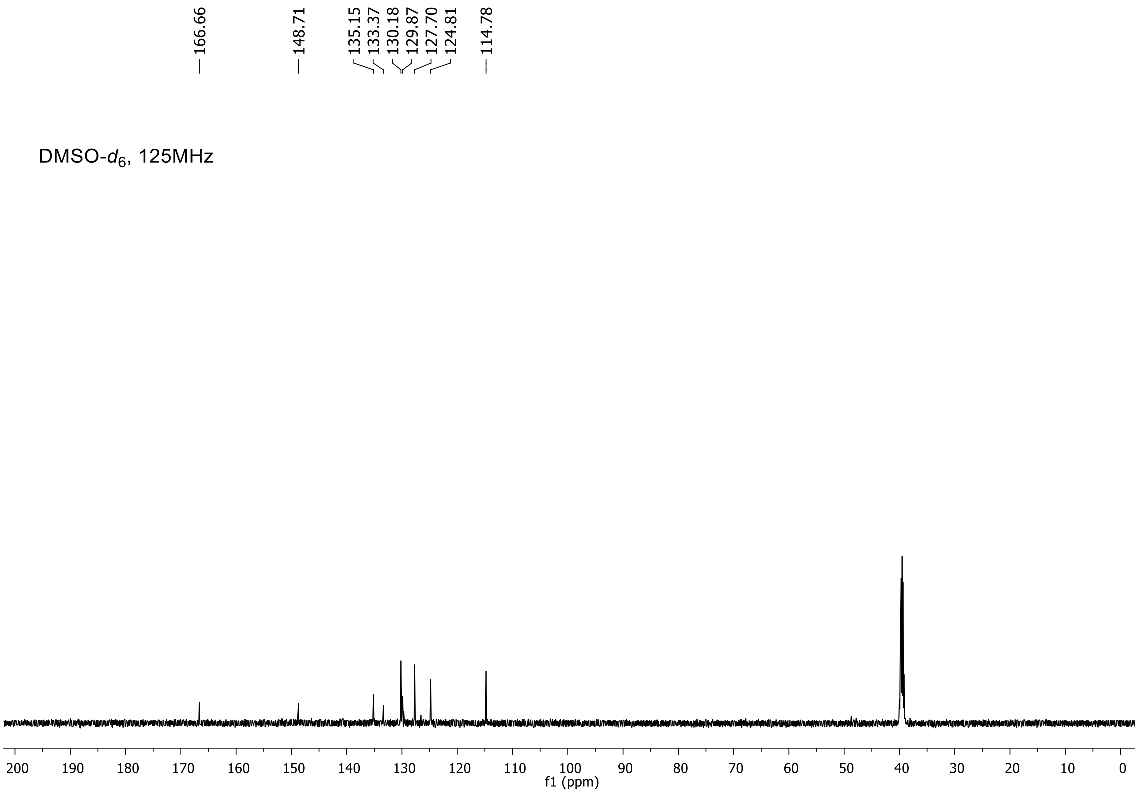
166.66
148.71
135.15
133.37
130.18
129.87
127.70
124.81
114.78
DMSO-d_6, 125MHz
200 190 180 170 160 150 140 130 120 110 100 90 80 70 60 50 40 30 20 10 0
f1 (ppm)

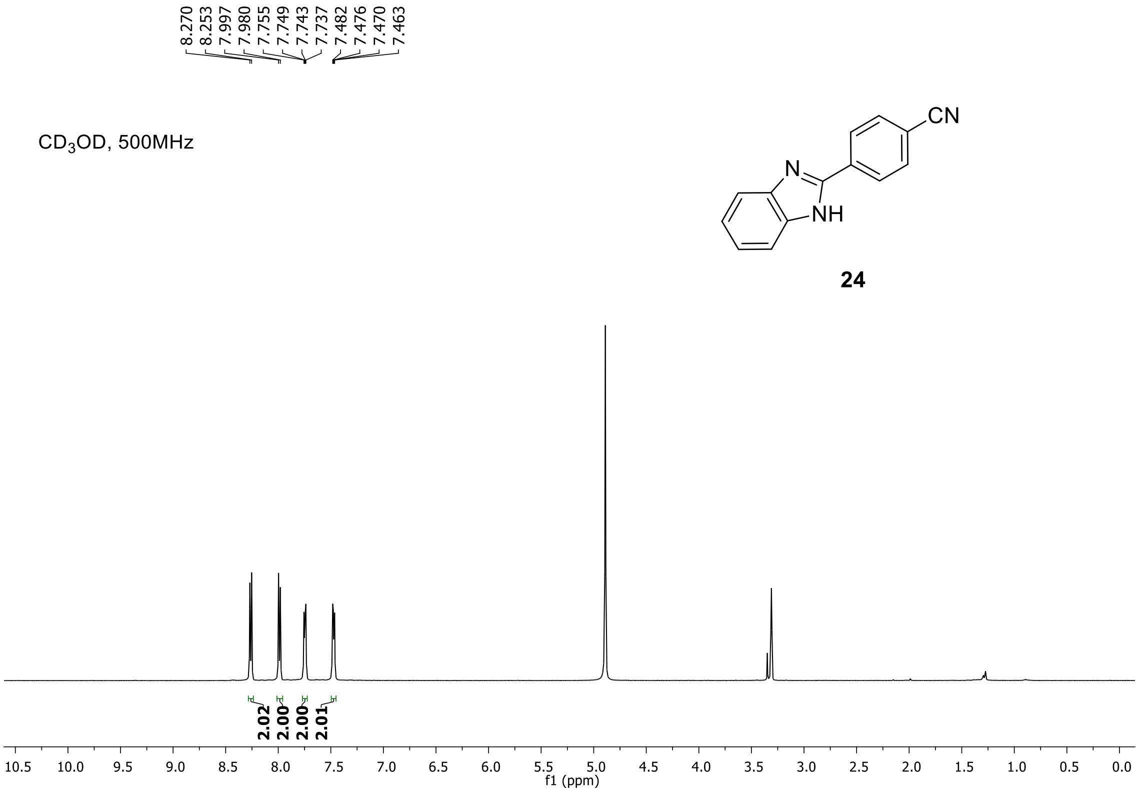

CD_3OD, 500MHz

24

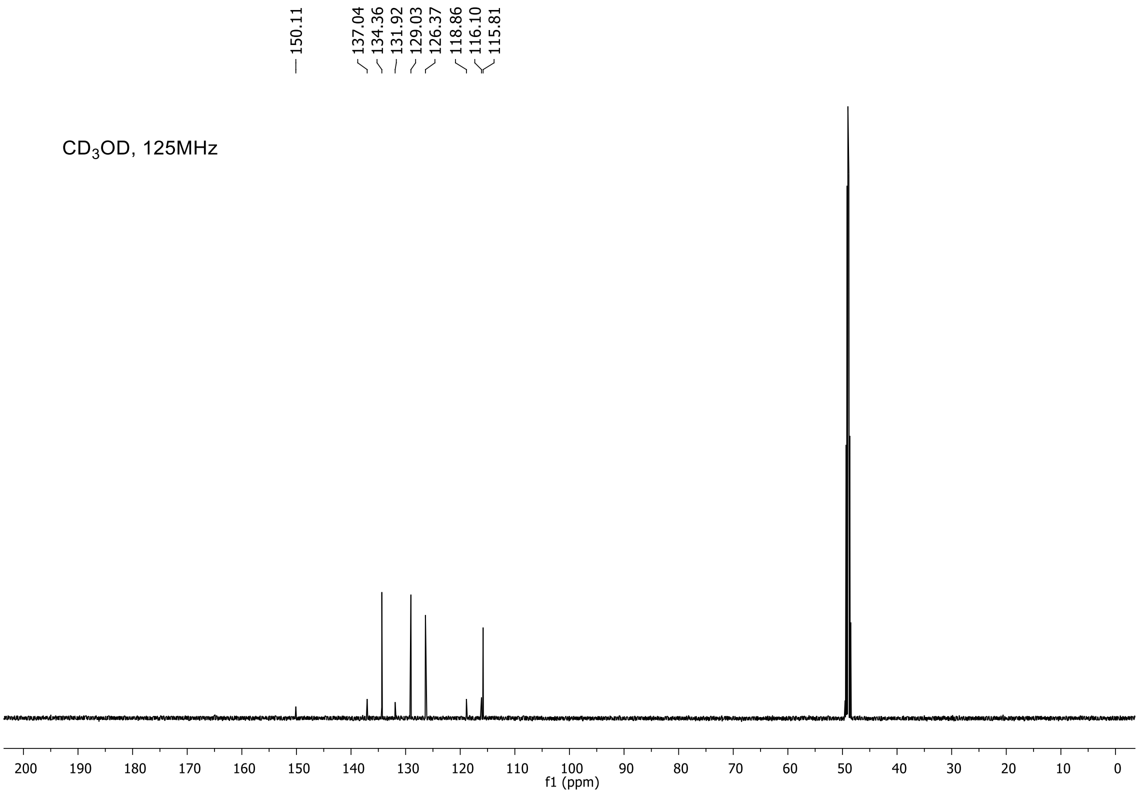

CD_3OD, 125MHz

8.858 8.515 8.499 8.200 8.198 8.184 8.181 7.696 7.690 7.684 7.678 7.594 7.578 7.562 7.313 7.307 7.301 7.295

CDCl$_3$, 500MHz

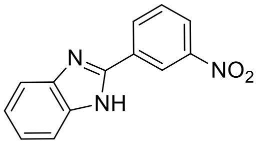

25

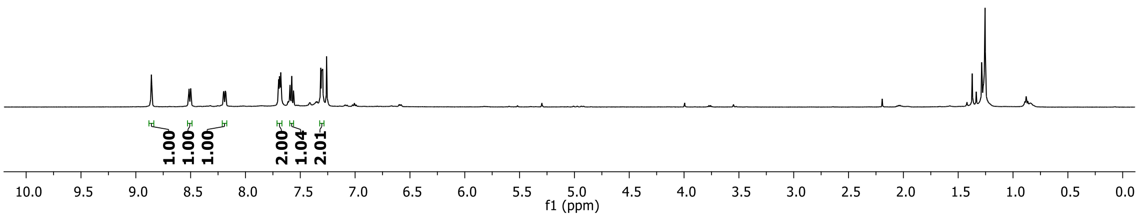

149.37 148.67 132.79 131.63 130.34 124.59 123.91 121.35 115.71

CDCl$_3$, 125MHz

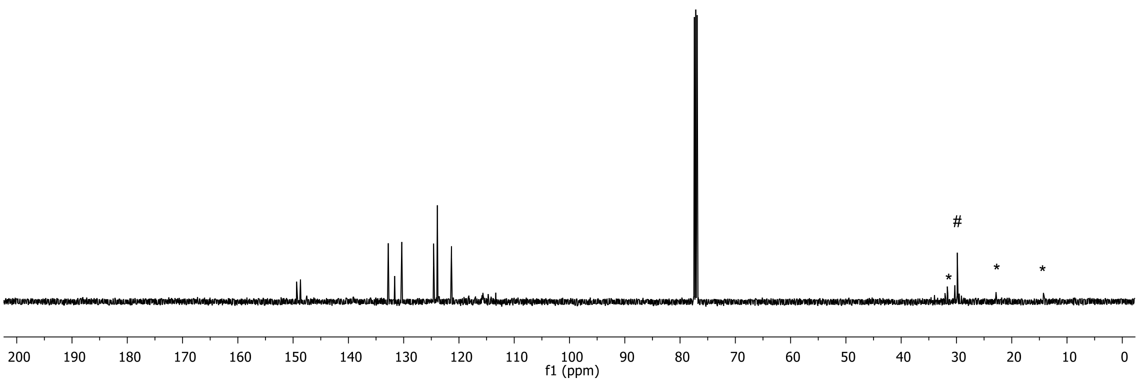

# = grease, * = hexane

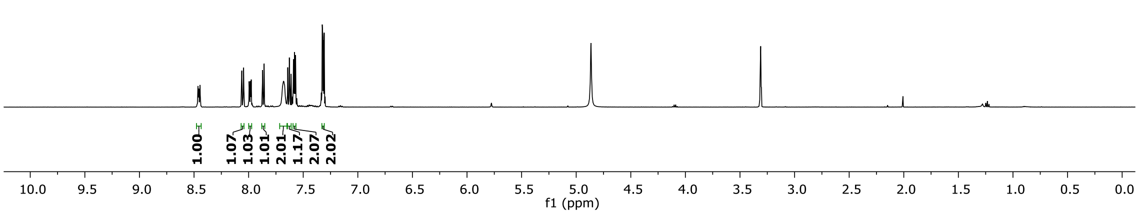
8.464
8.459
8.456
8.445
8.063
8.046
7.995
7.991
7.984
7.981
7.976
7.872
7.870
7.858
7.856
7.680
7.642
7.627
7.625
7.611
7.599
7.590
7.587
7.580
7.573
7.570
7.559
7.334
7.326
7.320
7.314
7.308
7.300
CD3OD, 500MHz
1.00
1.07
1.03
1.01
2.01
1.17
2.07
2.02
10.0 9.5 9.0 8.5 8.0 7.5 7.0 6.5 6.0 5.5 5.0 4.5 4.0 3.5 3.0 2.5 2.0 1.5 1.0 0.5 0.0
f1 (ppm)

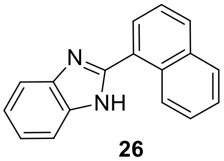
N
NH

26

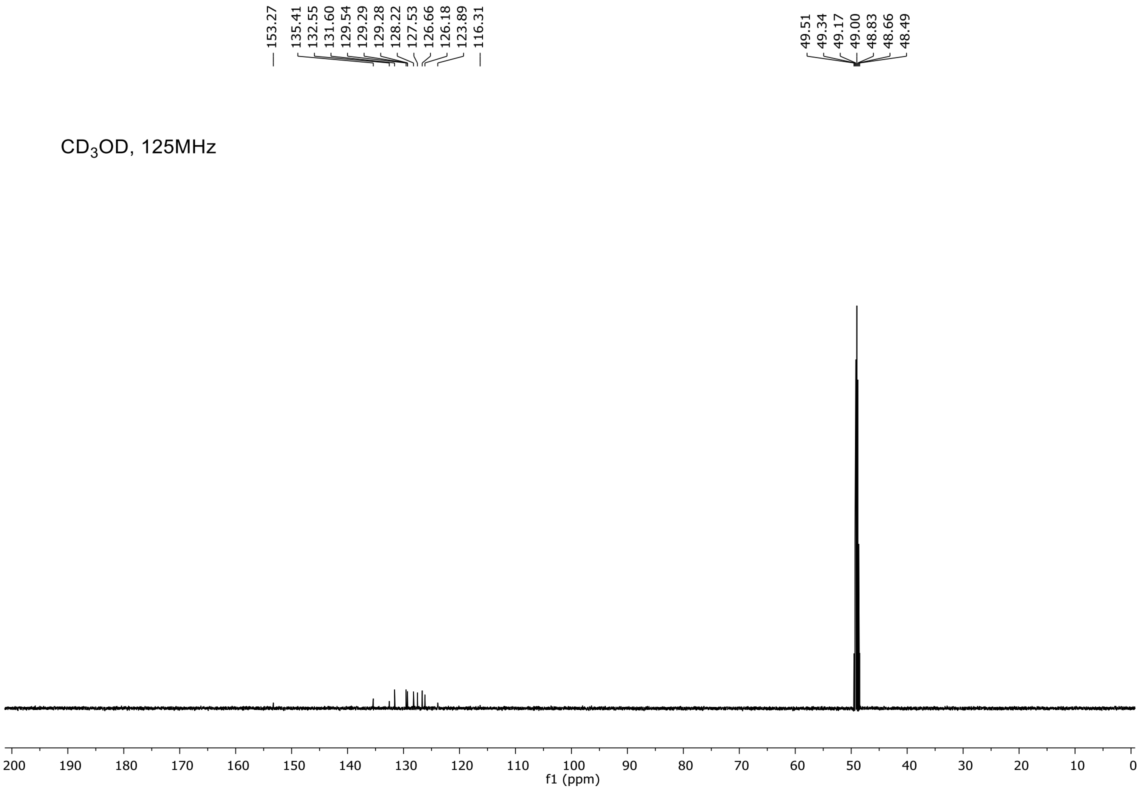
153.27
135.41
132.55
131.60
129.54
129.29
129.28
128.22
127.53
126.66
126.18
123.89
116.31
49.51
49.34
49.17
49.00
48.83
48.66
48.49
CD3OD, 125MHz
200 190 180 170 160 150 140 130 120 110 100 90 80 70 60 50 40 30 20 10 0
f1 (ppm)

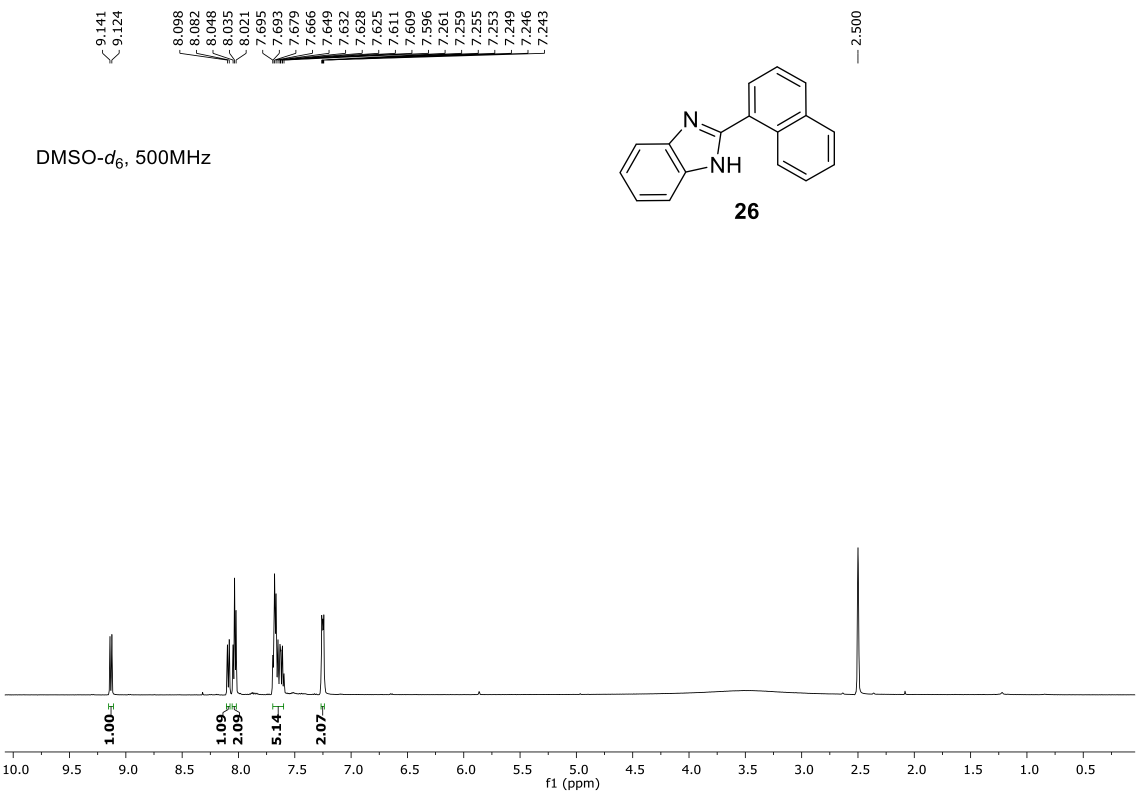
9.141
9.124
8.098
8.082
8.048
8.035
8.021
7.695
7.693
7.679
7.666
7.649
7.632
7.628
7.625
7.611
7.609
7.596
7.261
7.259
7.255
7.253
7.249
7.246
7.243
2.500
DMSO-d_6, 500MHz
N
NH
26
1.00
1.09
2.09
5.14
2.07
f1 (ppm)

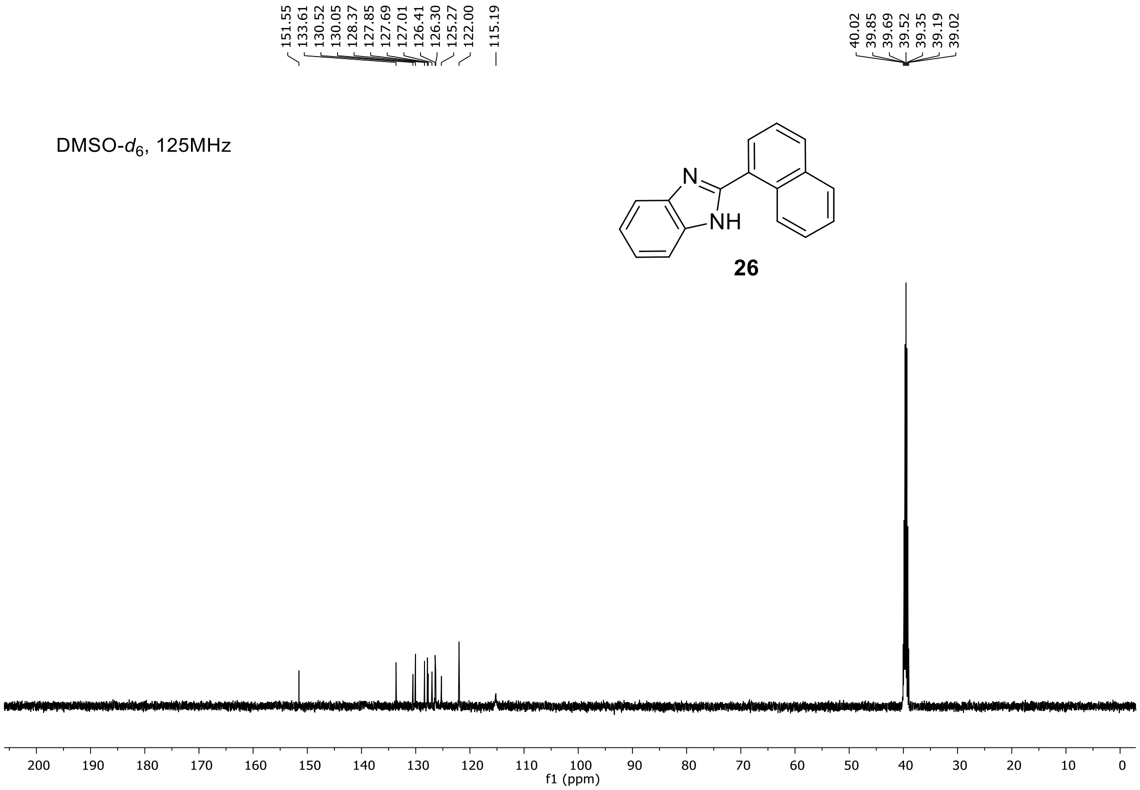
151.55
133.61
130.52
130.05
128.37
127.85
127.69
127.01
126.41
126.30
125.27
122.00
115.19
40.02
39.85
39.69
39.52
39.35
39.19
39.02
DMSO-d_6, 125MHz
N
NH
26
f1 (ppm)

CD$_3$OD, 500MHz

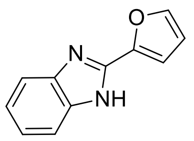

27

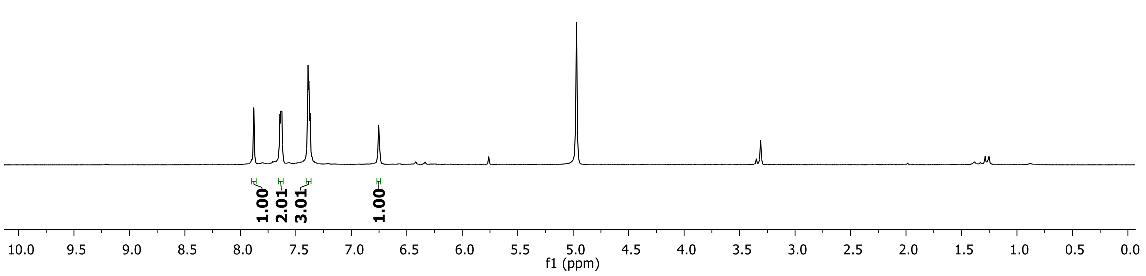

CD$_3$OD, 125MHz

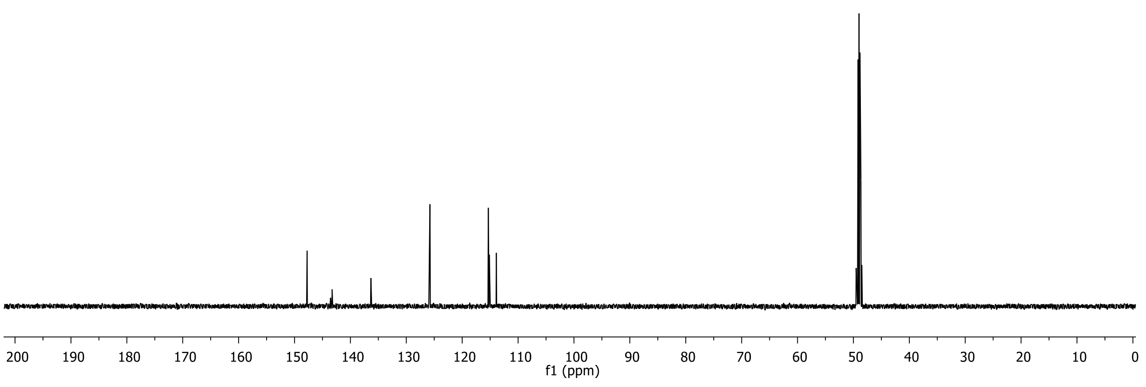

CD$_3$OD, 500MHz

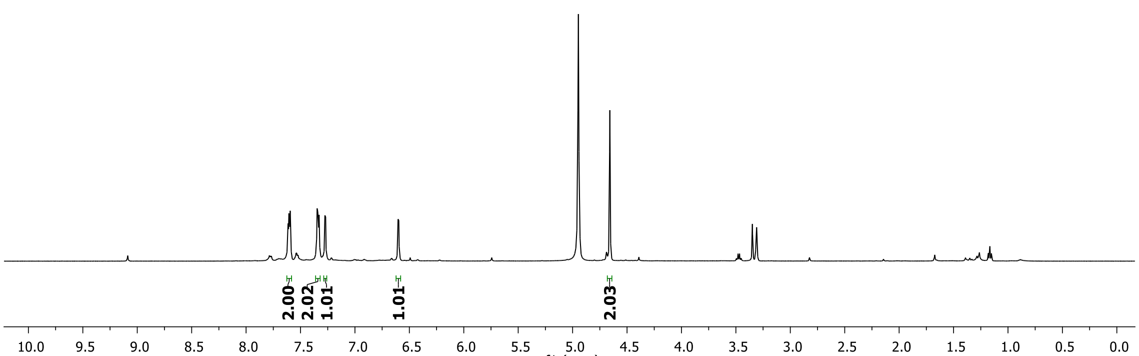

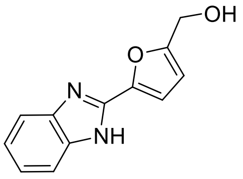

28

CD$_3$OD, 125MHz

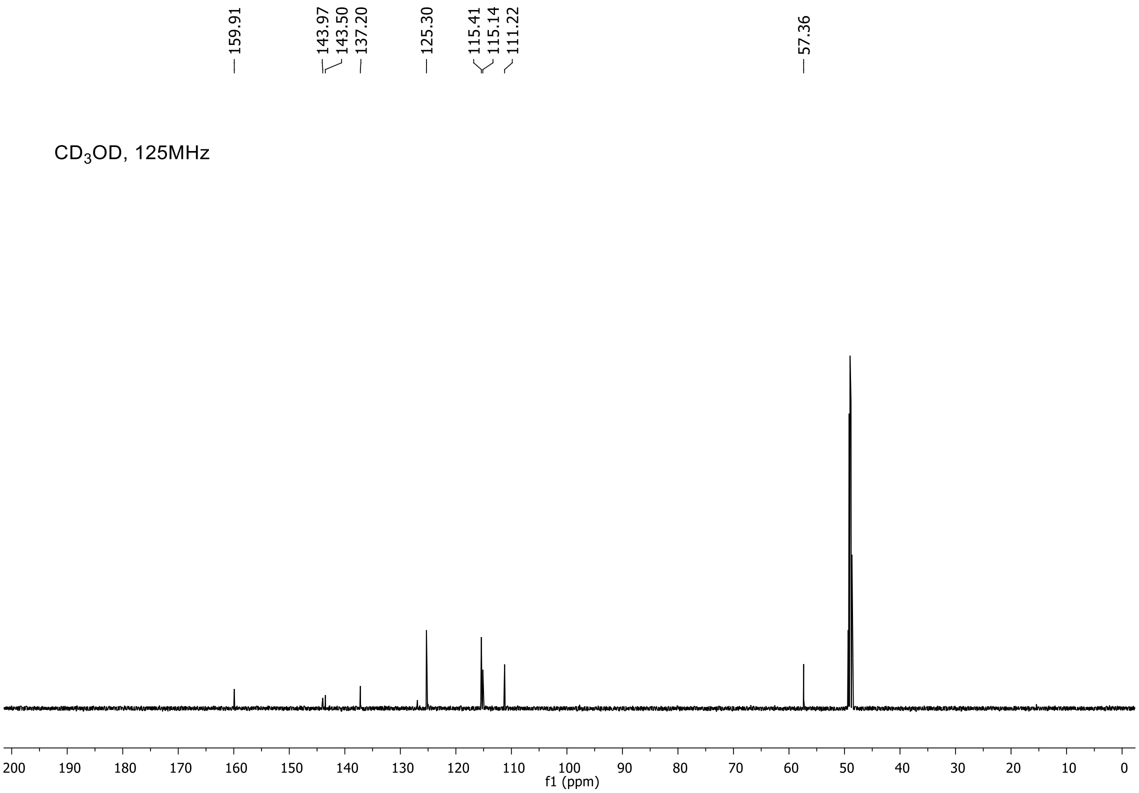

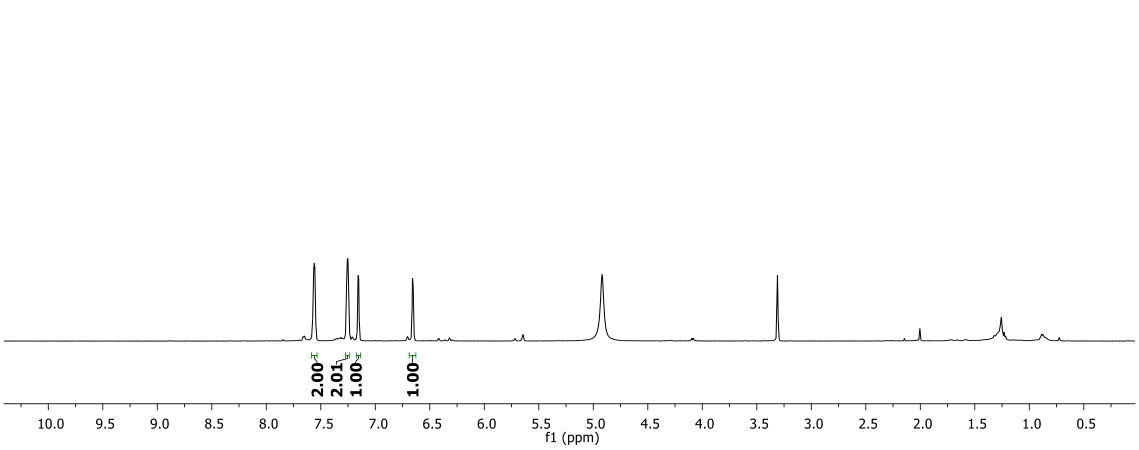
7.568
7.563
7.560
7.555
7.261
7.256
7.251
7.246
7.158
7.152
6.659
6.653
CD3OD, 600MHz
2.00
2.01
1.00
1.00
10.0 9.5 9.0 8.5 8.0 7.5 7.0 6.5 6.0 5.5 5.0 4.5 4.0 3.5 3.0 2.5 2.0 1.5 1.0 0.5
f1 (ppm)

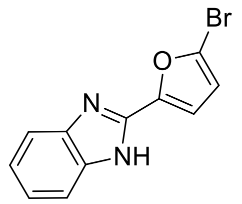
Br
O
N
NH

29

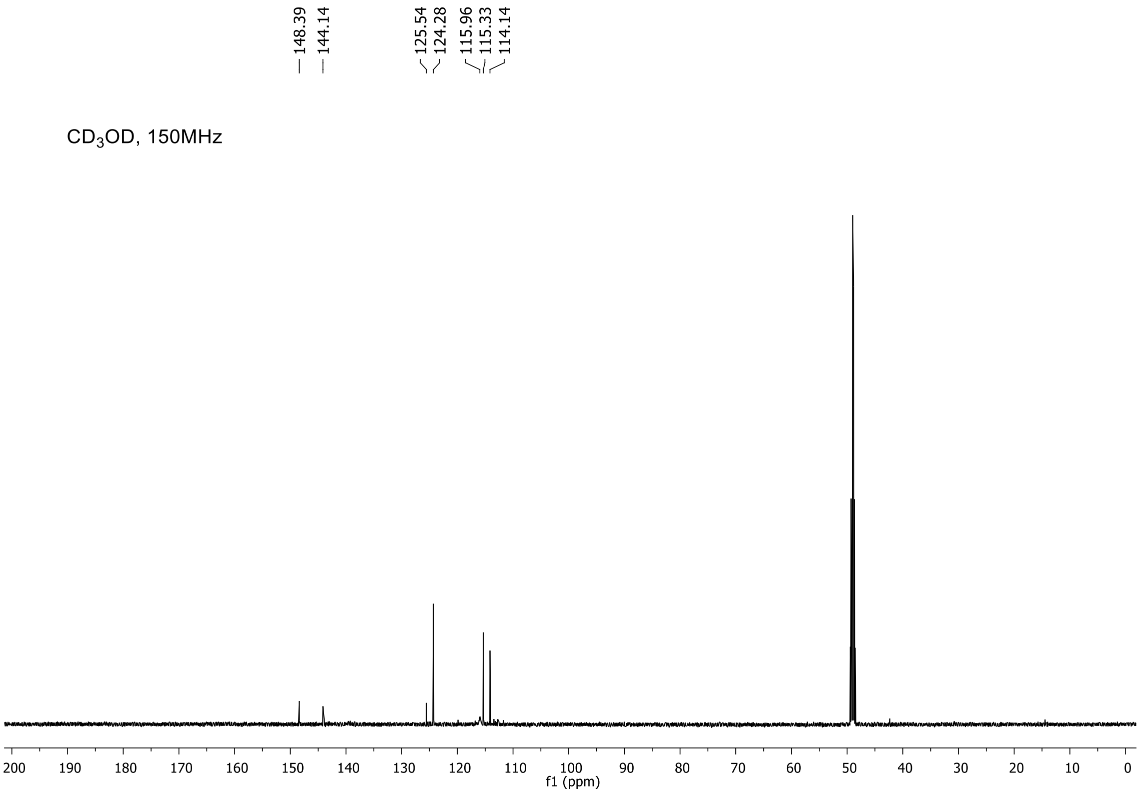
148.39
144.14
125.54
124.28
115.96
115.33
114.14
CD3OD, 150MHz
200 190 180 170 160 150 140 130 120 110 100 90 80 70 60 50 40 30 20 10 0
f1 (ppm)

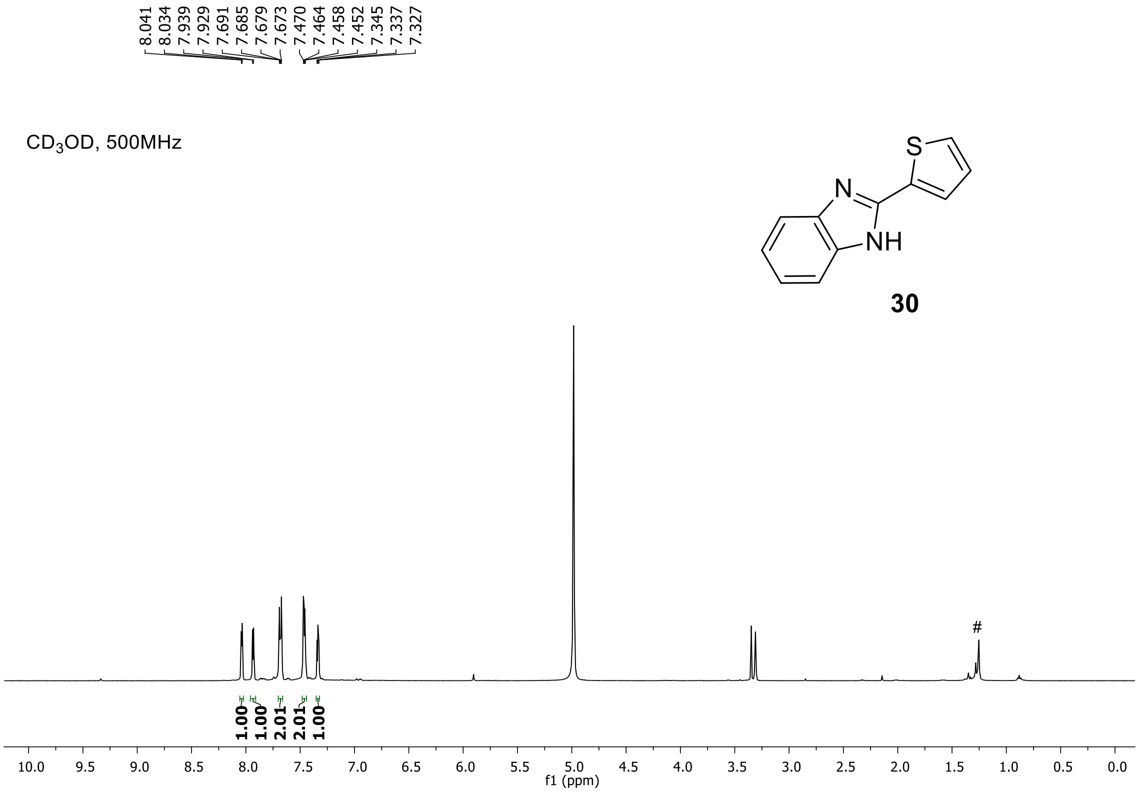

8.041 8.034 7.939 7.929 7.691 7.685 7.679 7.673 7.470 7.464 7.458 7.452 7.345 7.337 7.327
CD3OD, 500MHz
S
N
NH
30
#
1.00 1.00 2.01 2.01 1.00
10.0 9.5 9.0 8.5 8.0 7.5 7.0 6.5 6.0 5.5 5.0 4.5 4.0 3.5 3.0 2.5 2.0 1.5 1.0 0.5 0.0
f1 (ppm)

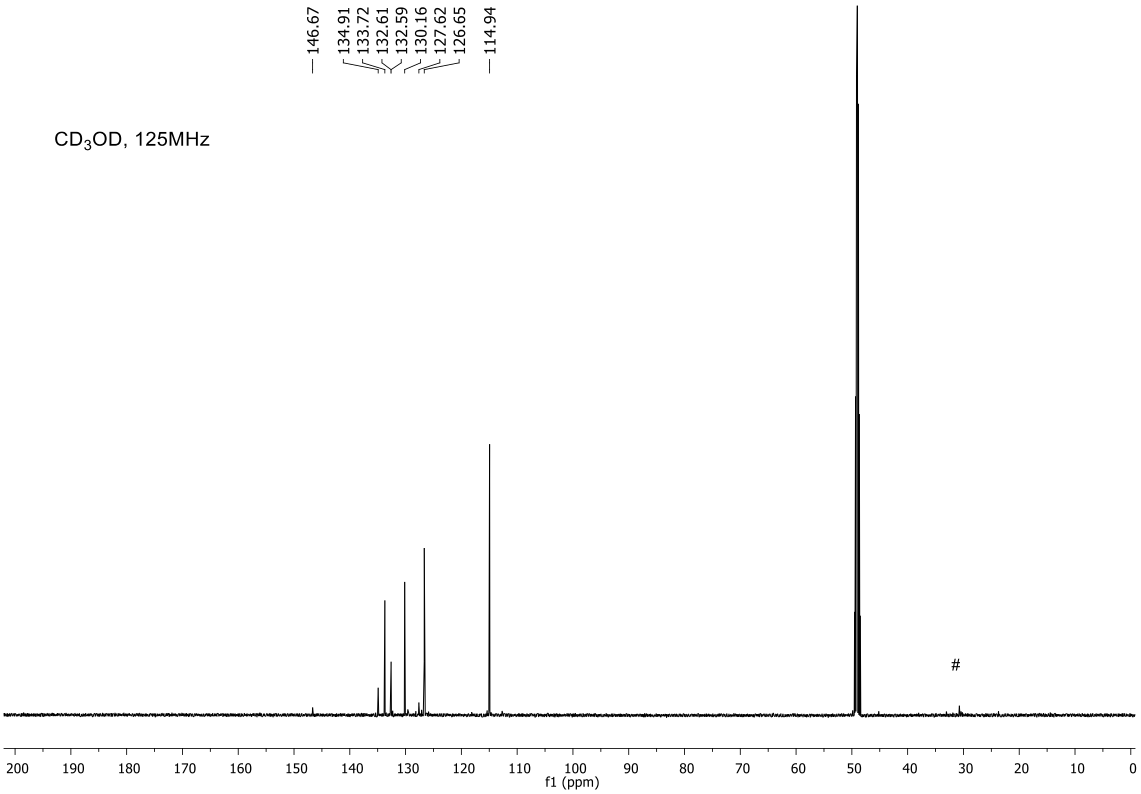

146.67
134.91 133.72 132.61 132.59 130.16 127.62 126.65
114.94
CD3OD, 125MHz
#
200 190 180 170 160 150 140 130 120 110 100 90 80 70 60 50 40 30 20 10 0
f1 (ppm)

# = grease

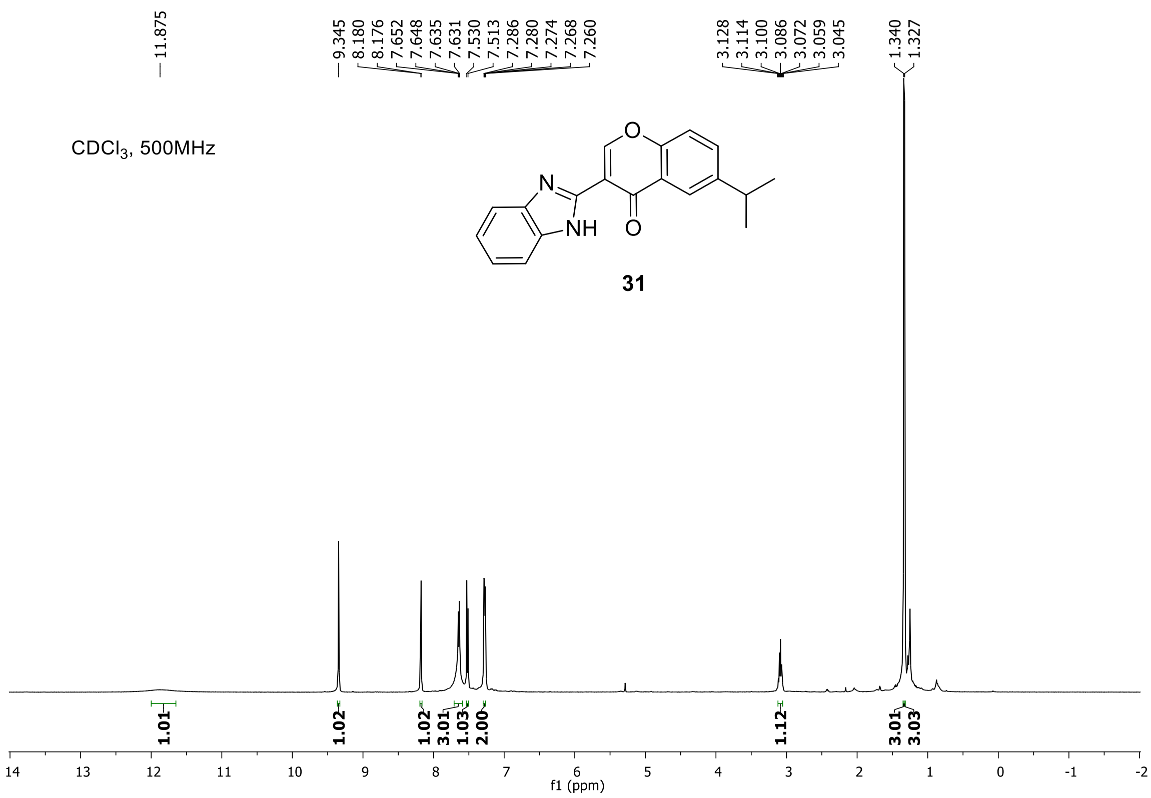

11.875
9.345
8.180
8.176
7.652
7.648
7.635
7.631
7.530
7.513
7.286
7.280
7.274
7.268
7.260
3.128
3.114
3.100
3.086
3.072
3.059
3.045
1.340
1.327
CDCl3, 500MHz
N
NH
O
O
31
1.01
1.02
1.02
3.01
1.03
2.00
1.12
3.01
3.03
f1 (ppm)

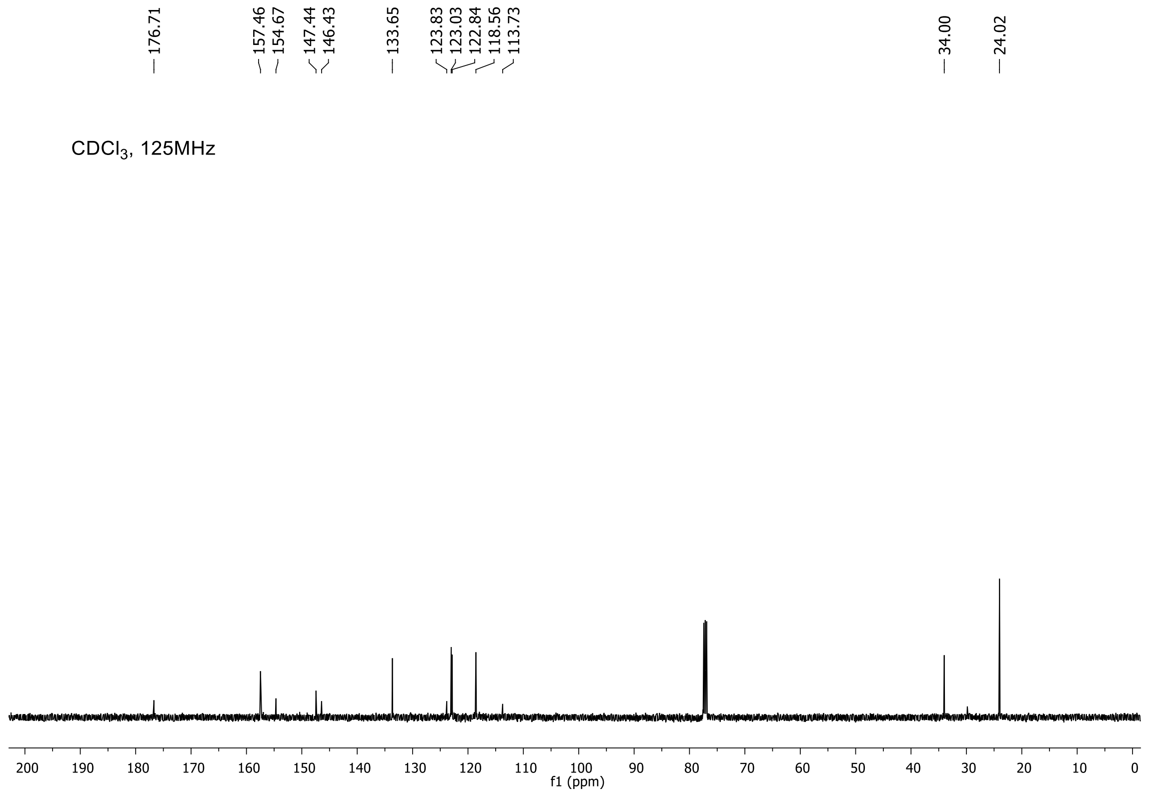

176.71
157.46
154.67
147.44
146.43
133.65
123.83
123.03
122.84
118.56
113.73
34.00
24.02
CDCl3, 125MHz
f1 (ppm)

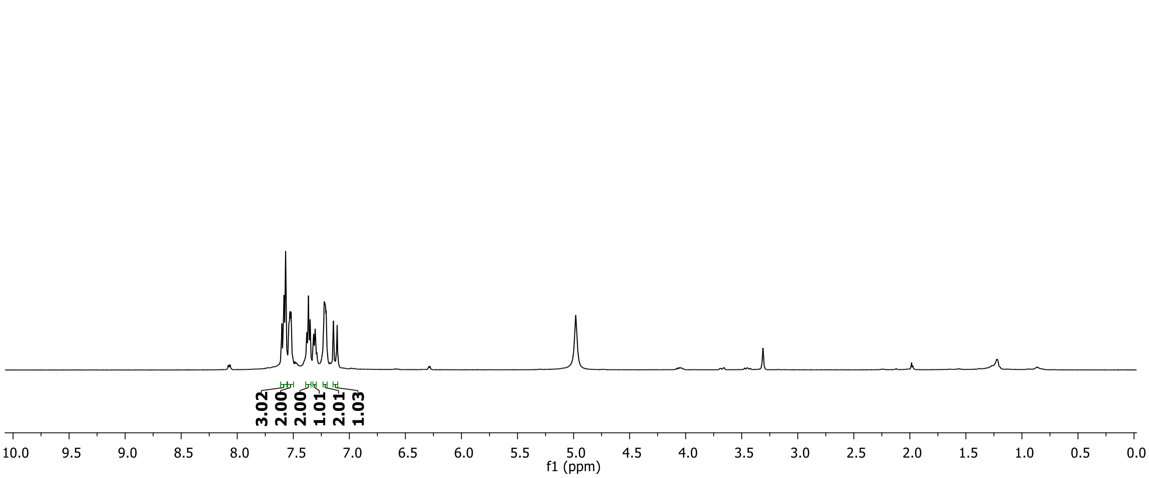

CD$_3$OD, 500MHz

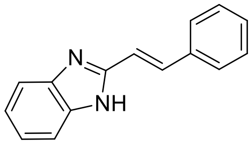

32

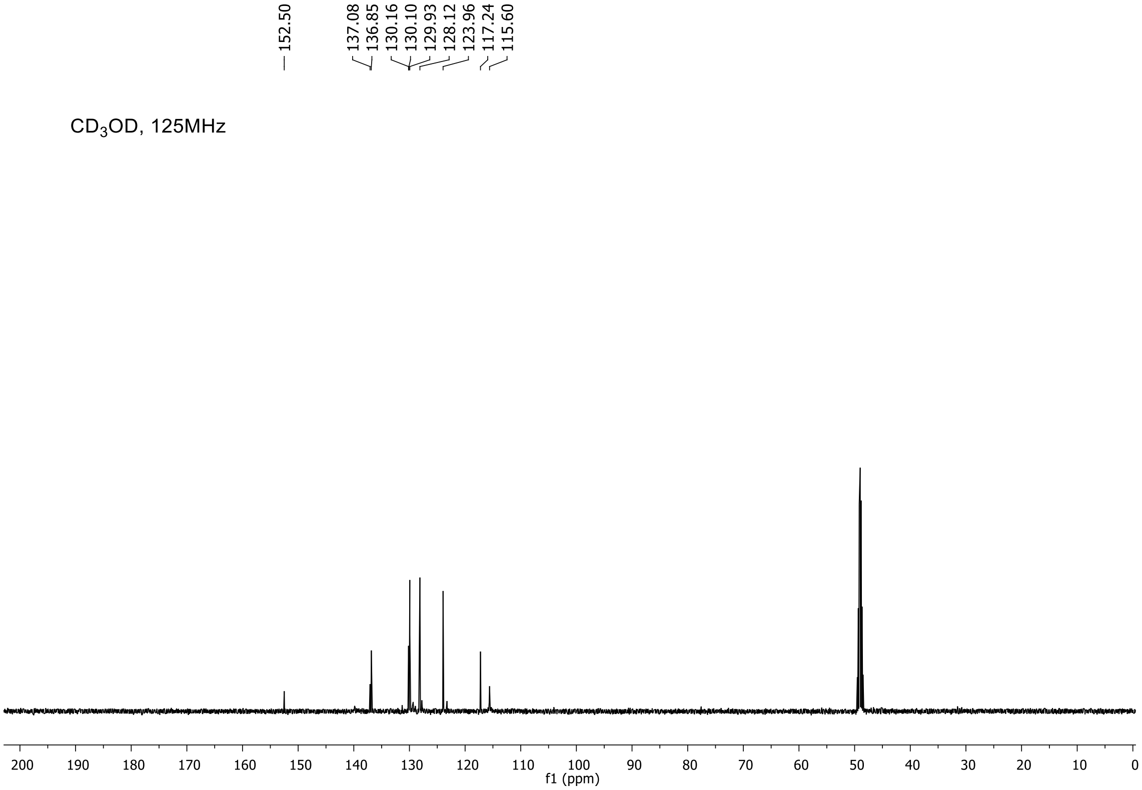

CD$_3$OD, 125MHz

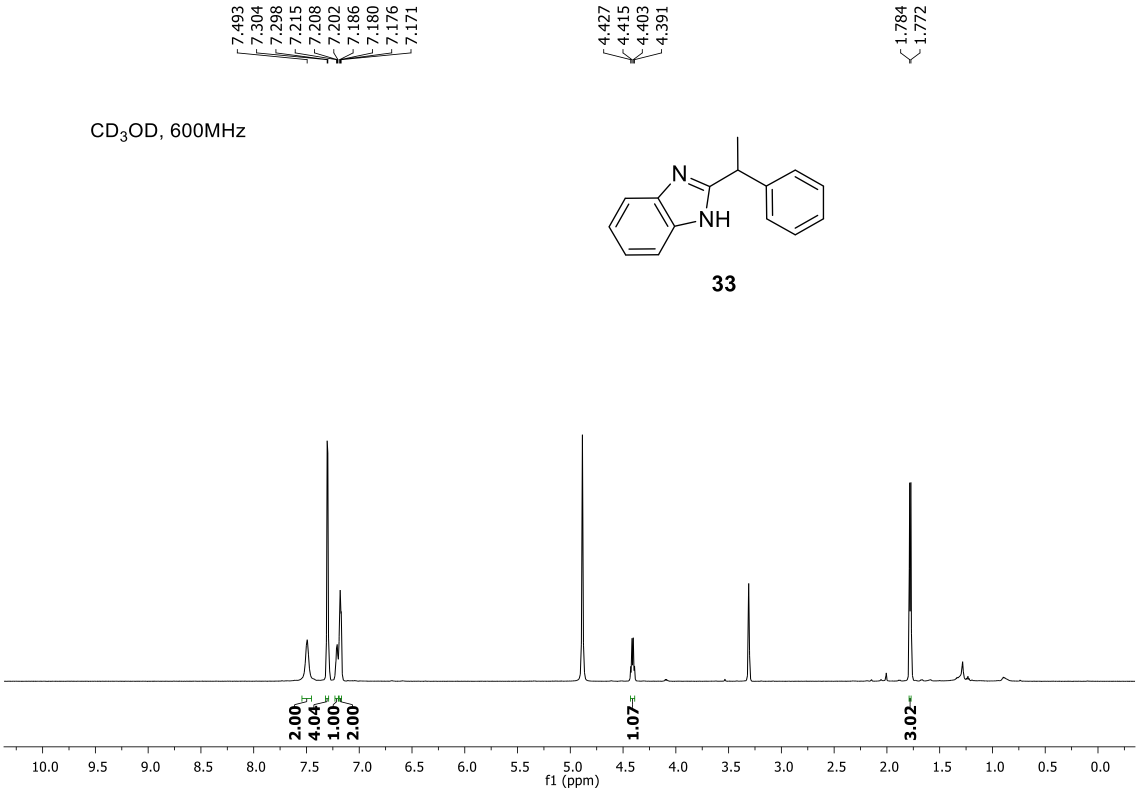
7.493
7.304
7.298
7.215
7.208
7.202
7.186
7.180
7.176
7.171
4.427
4.415
4.403
4.391
1.784
1.772
CD$_3$OD, 600MHz
2.00
4.04
1.00
2.00
1.07
3.02
10.0
9.5
9.0
8.5
8.0
7.5
7.0
6.5
6.0
5.5
5.0
4.5
4.0
3.5
3.0
2.5
2.0
1.5
1.0
0.5
0.0
f1 (ppm)
N
NH

33

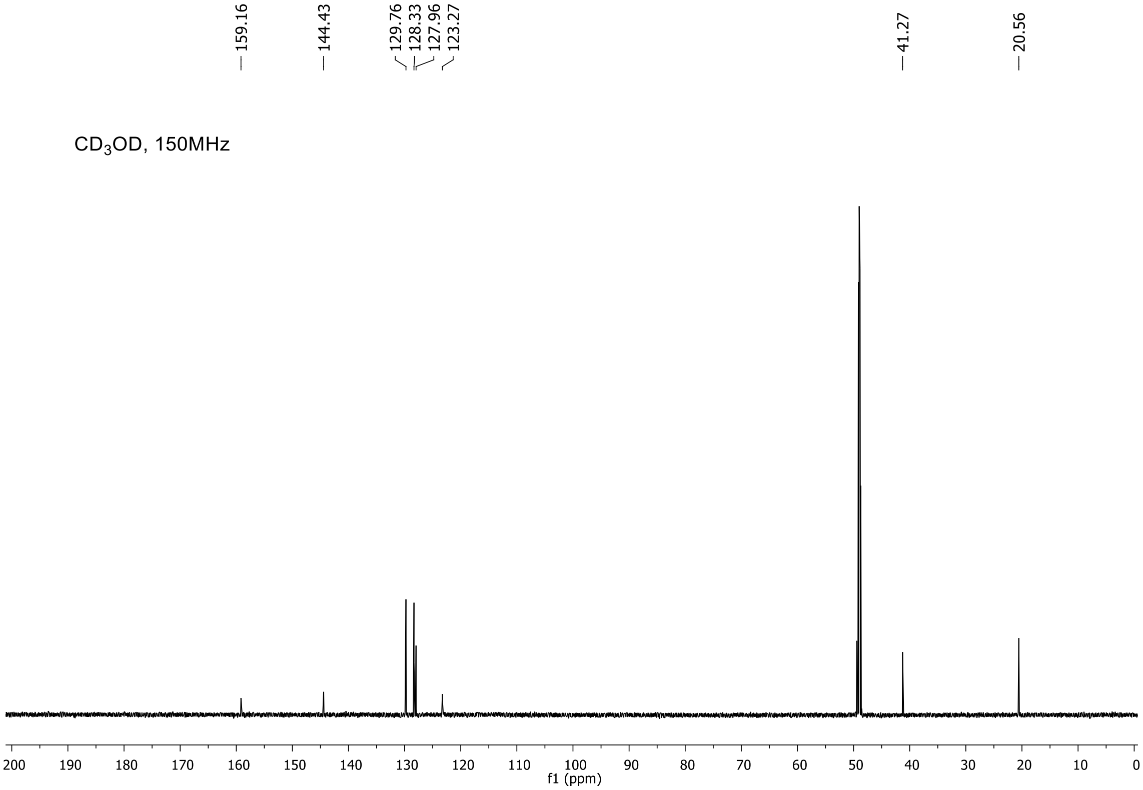
159.16
144.43
129.76
128.33
127.96
123.27
41.27
20.56
CD$_3$OD, 150MHz
200
190
180
170
160
150
140
130
120
110
100
90
80
70
60
50
40
30
20
10
0
f1 (ppm)

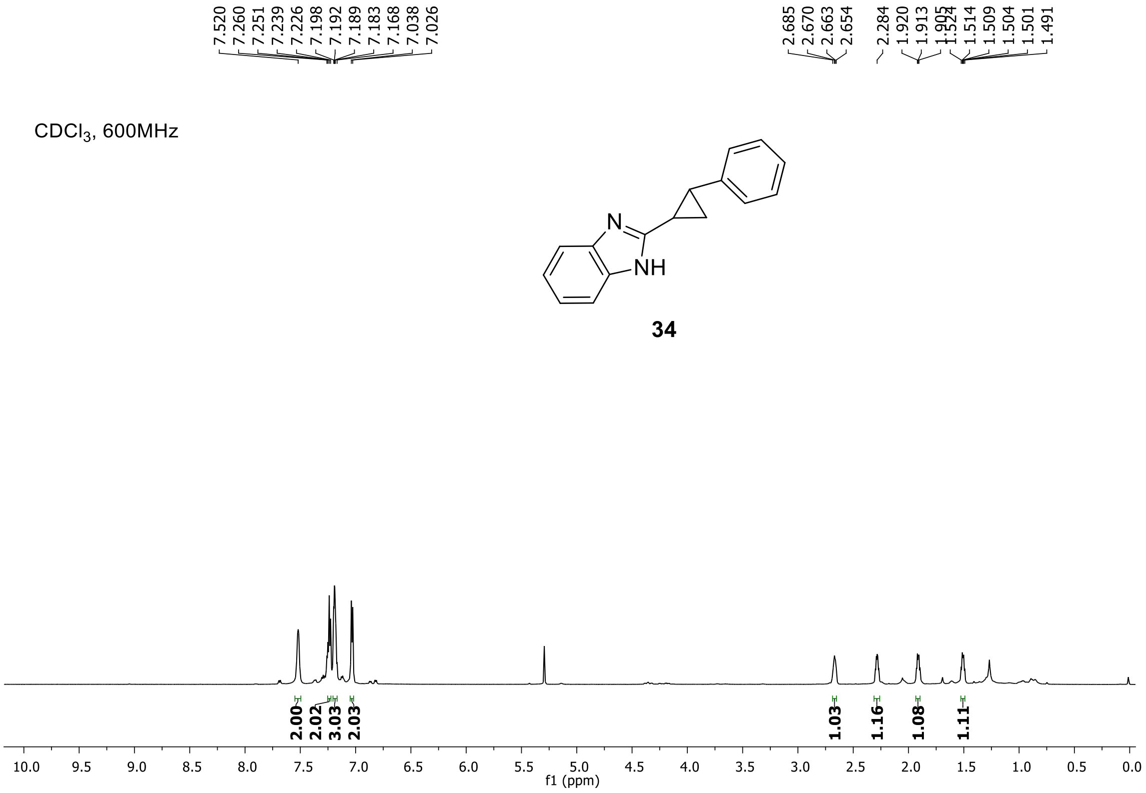
7.520
7.260
7.251
7.239
7.226
7.198
7.192
7.189
7.183
7.168
7.038
7.026
2.685
2.670
2.663
2.654
2.284
1.920
1.913
1.905
1.524
1.514
1.509
1.504
1.501
1.491
CDCl3, 600MHz
N
NH
34
2.00
2.02
3.03
2.03
1.03
1.16
1.08
1.11
10.0 9.5 9.0 8.5 8.0 7.5 7.0 6.5 6.0 5.5 5.0 4.5 4.0 3.5 3.0 2.5 2.0 1.5 1.0 0.5 0.0
f1 (ppm)

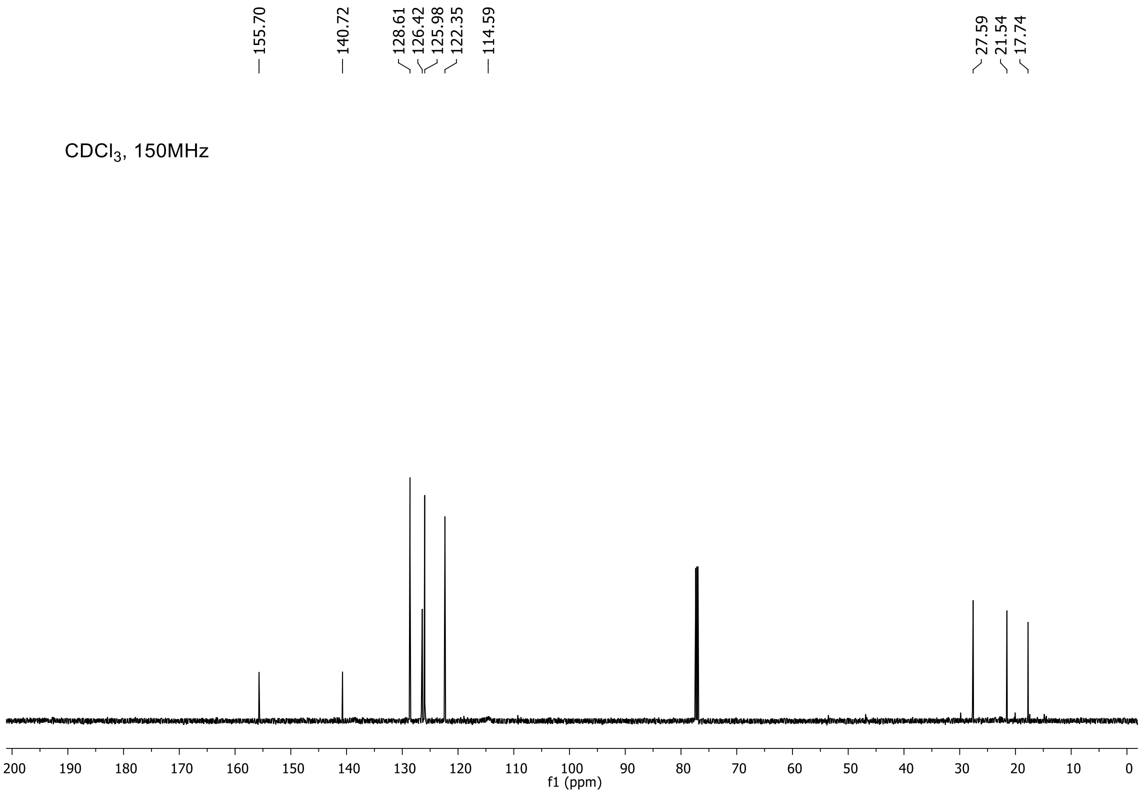
155.70
140.72
128.61
126.42
125.98
122.35
114.59
27.59
21.54
17.74
CDCl3, 150MHz
200 190 180 170 160 150 140 130 120 110 100 90 80 70 60 50 40 30 20 10 0
f1 (ppm)

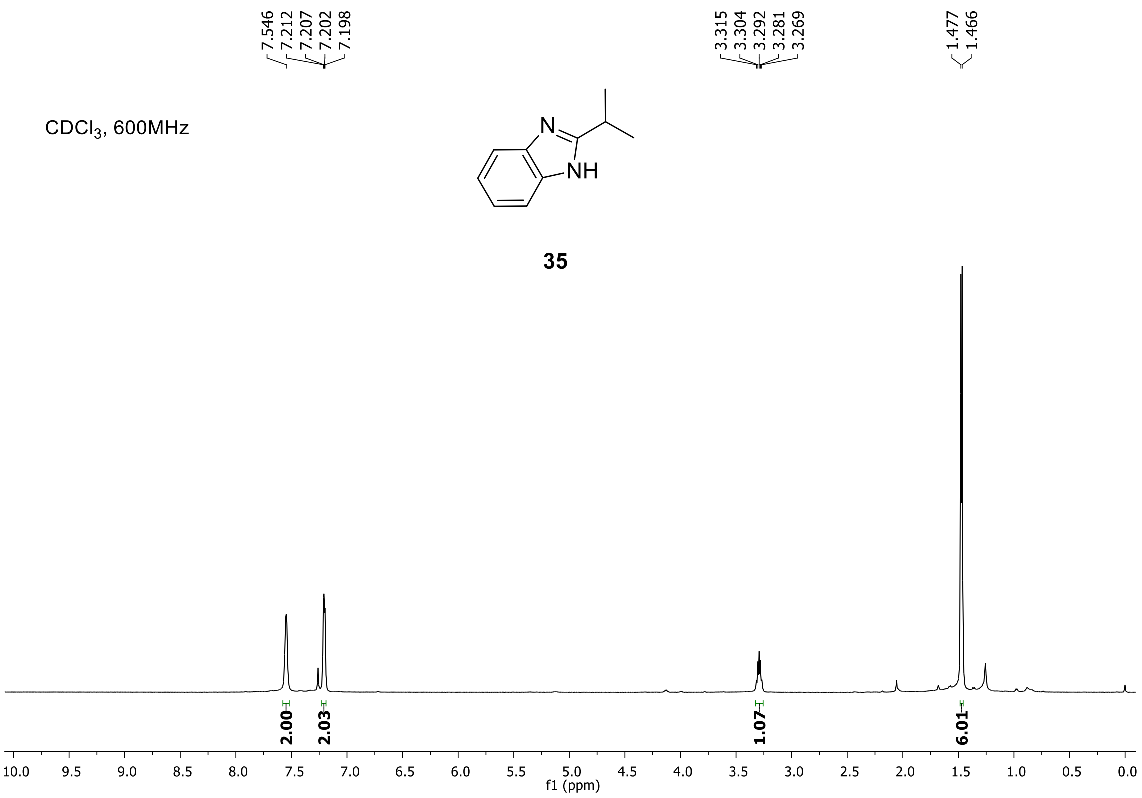
CDCl$_3$, 600MHz
35
7.546
7.212
7.207
7.202
7.198
3.315
3.304
3.292
3.281
3.269
1.477
1.466
2.00
2.03
1.07
6.01
f1 (ppm)

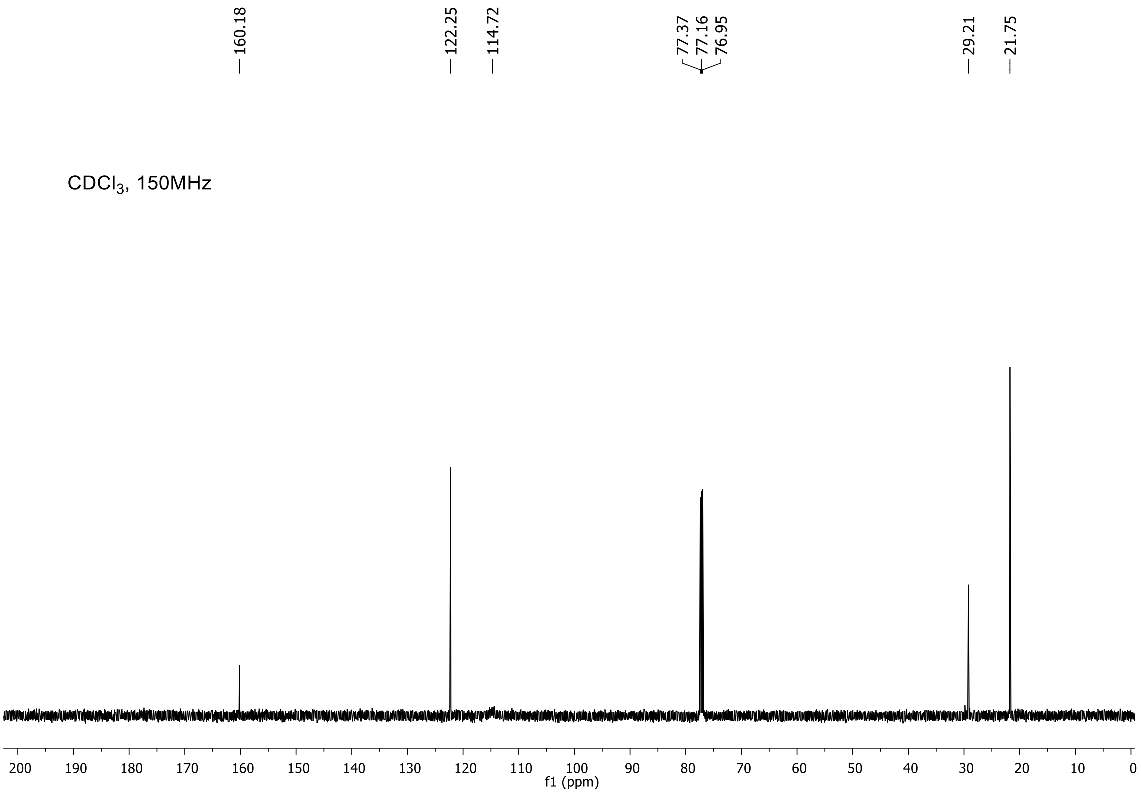
CDCl$_3$, 150MHz
160.18
122.25
114.72
77.37
77.16
76.95
29.21
21.75
f1 (ppm)

CDCl$_3$, 600MHz

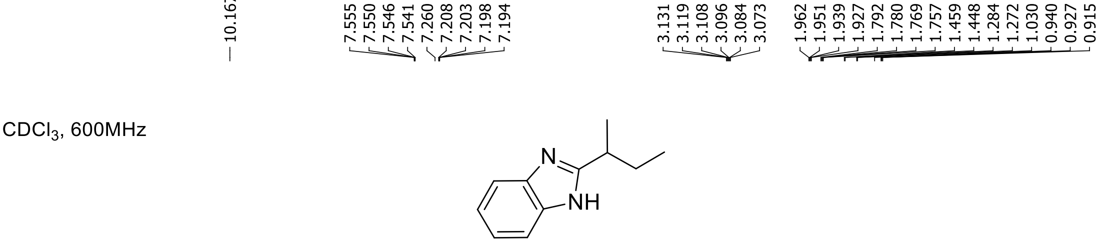

36

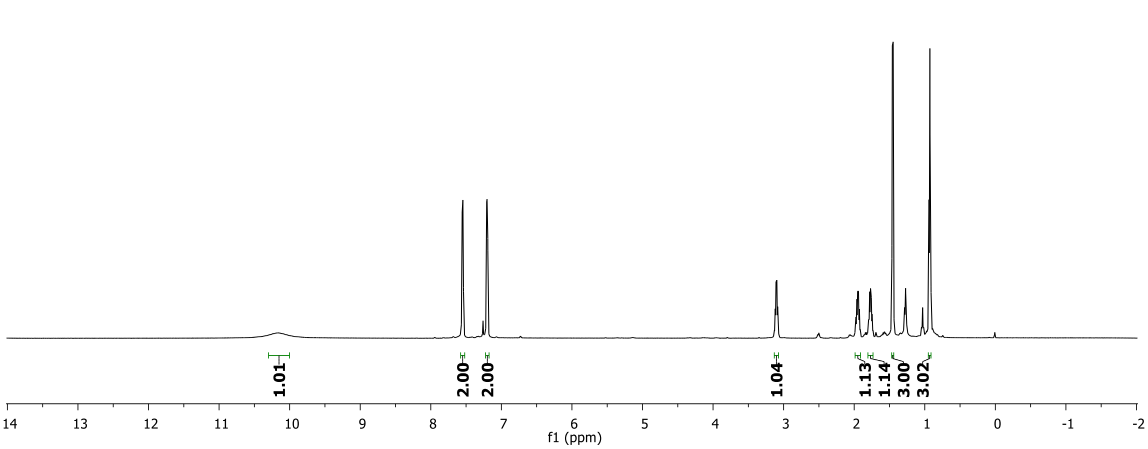

CDCl$_3$, 150MHz

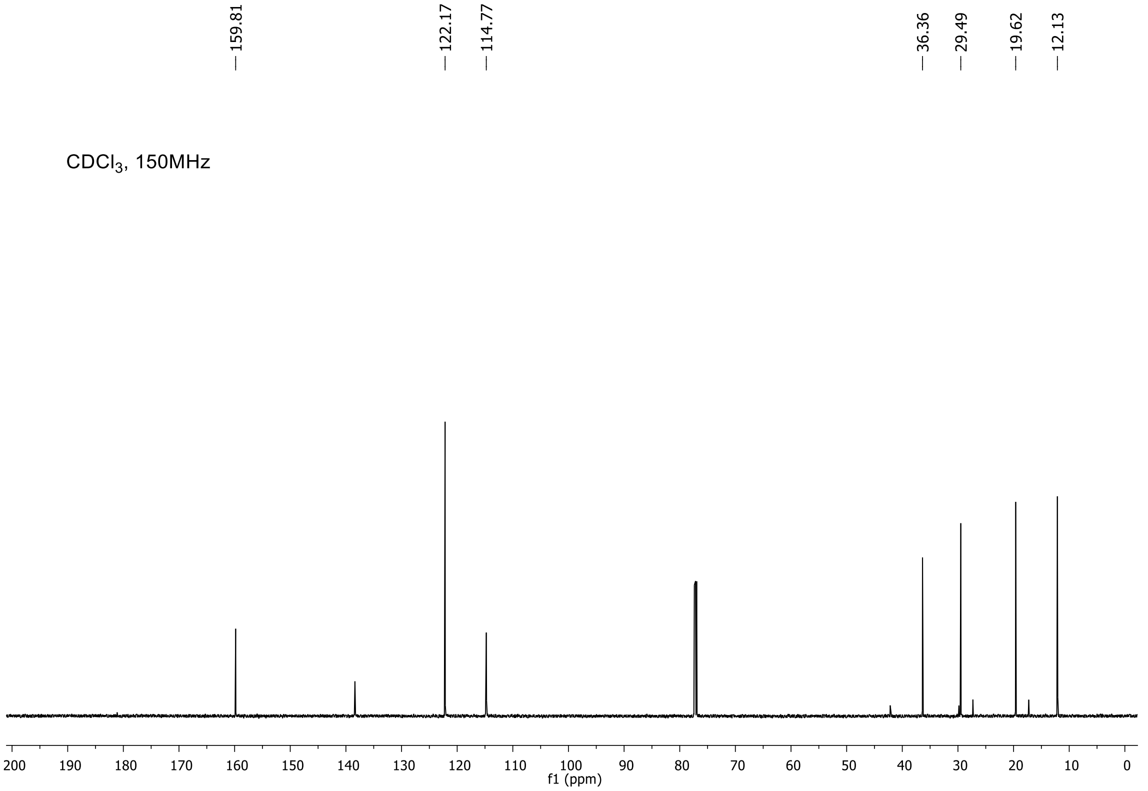

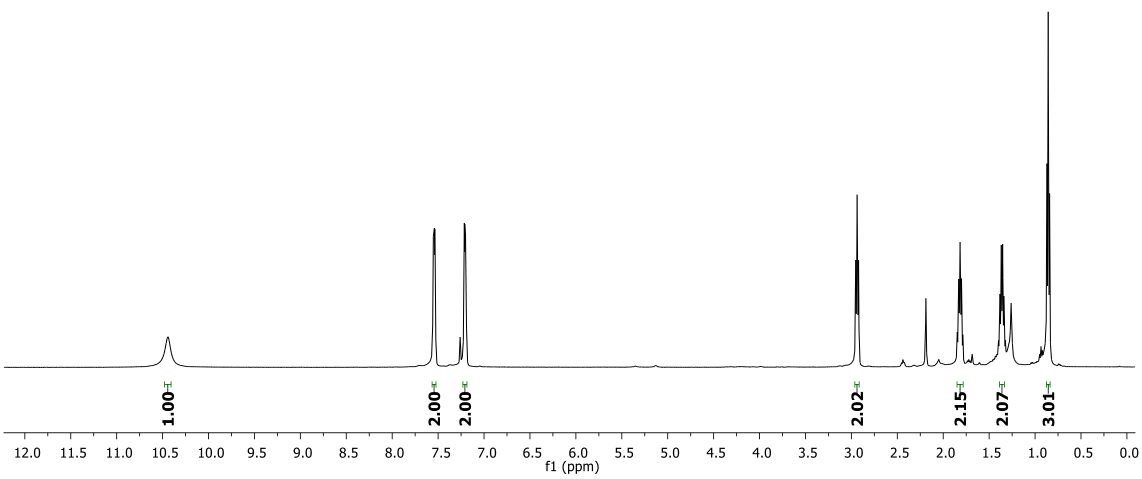

CDCl$_3$, 500MHz

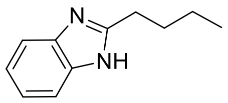

37

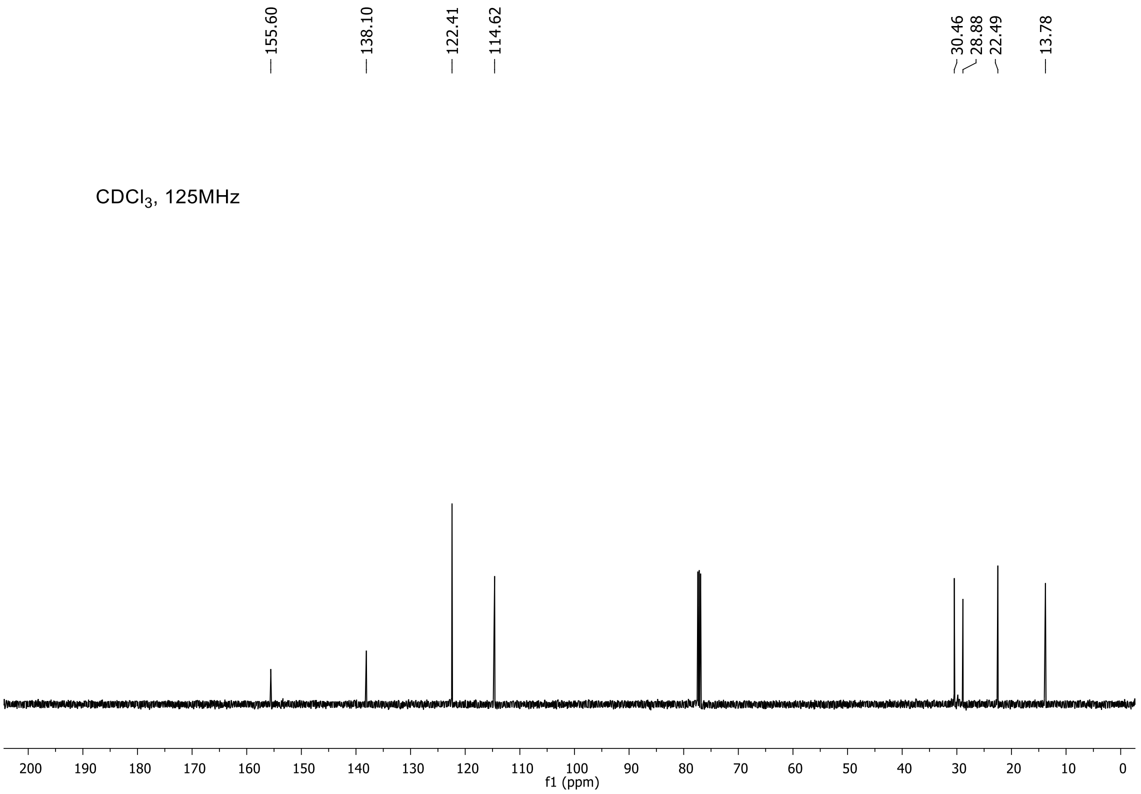

CDCl$_3$, 125MHz

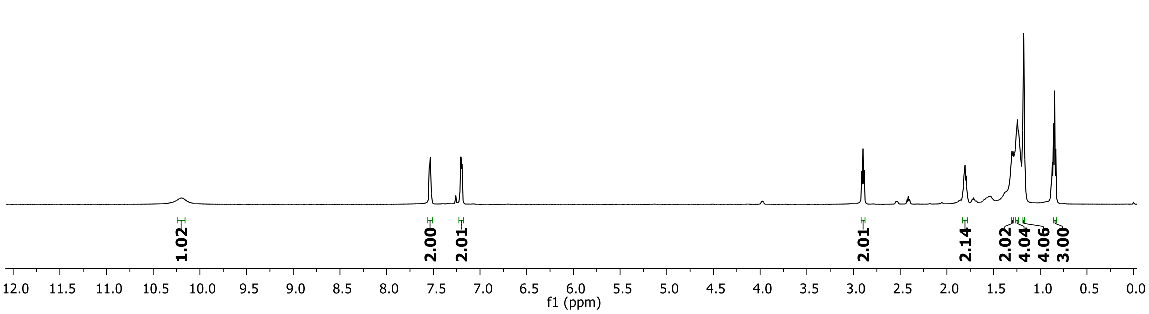

$CDCl_3$, 600MHz

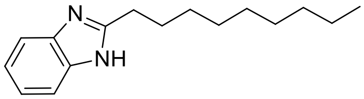

39

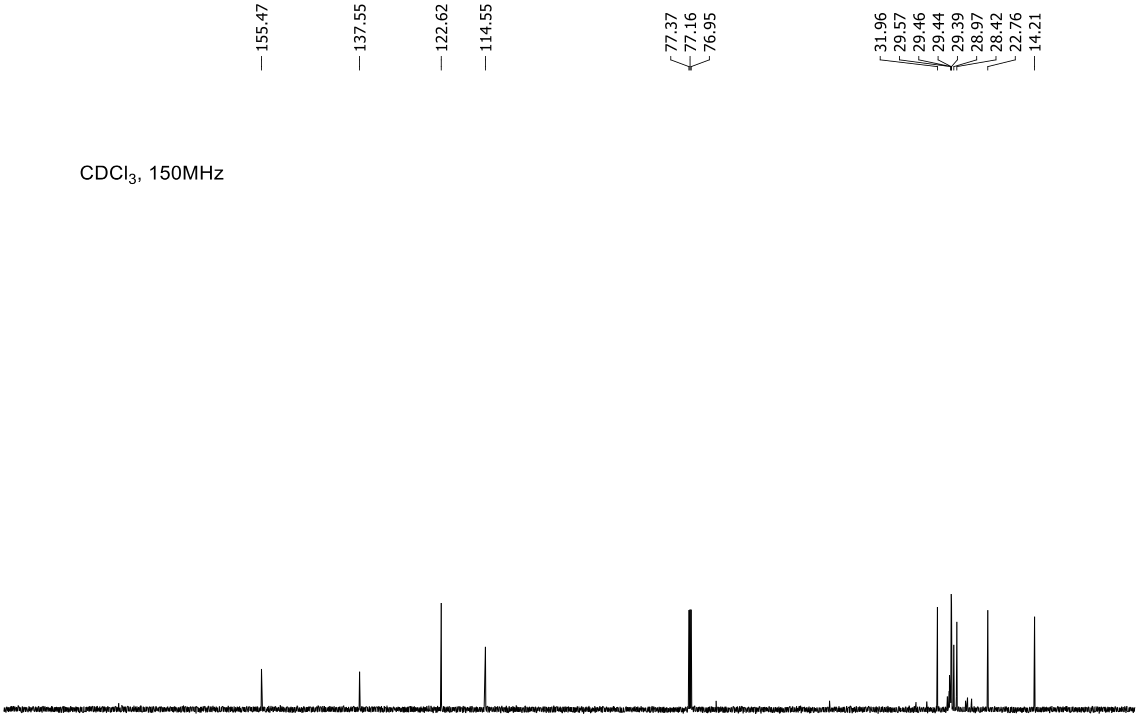

$CDCl_3$, 150MHz

CD$_3$OD, 600MHz

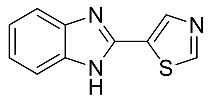

44

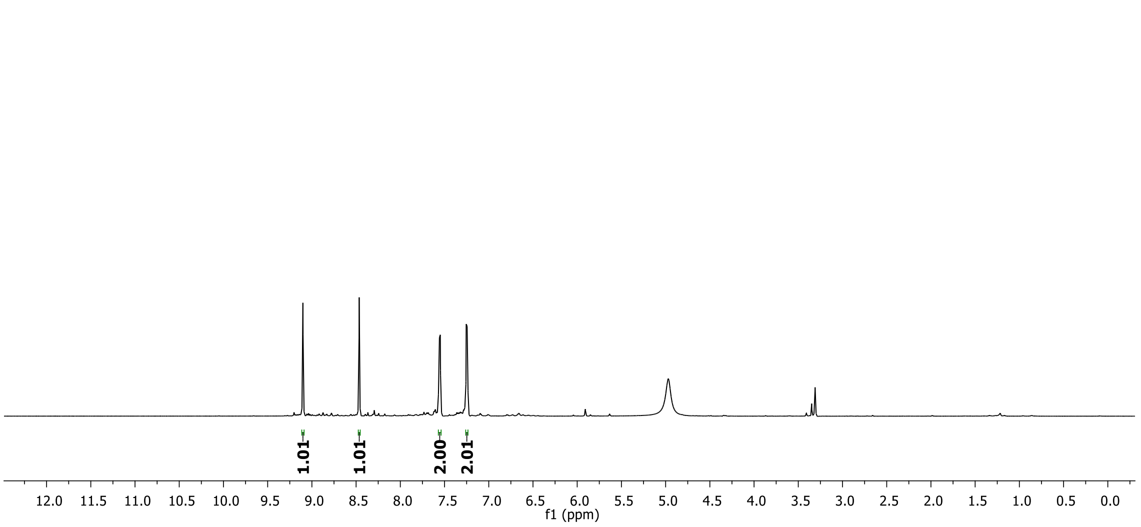

CD$_3$OD, 150MHz

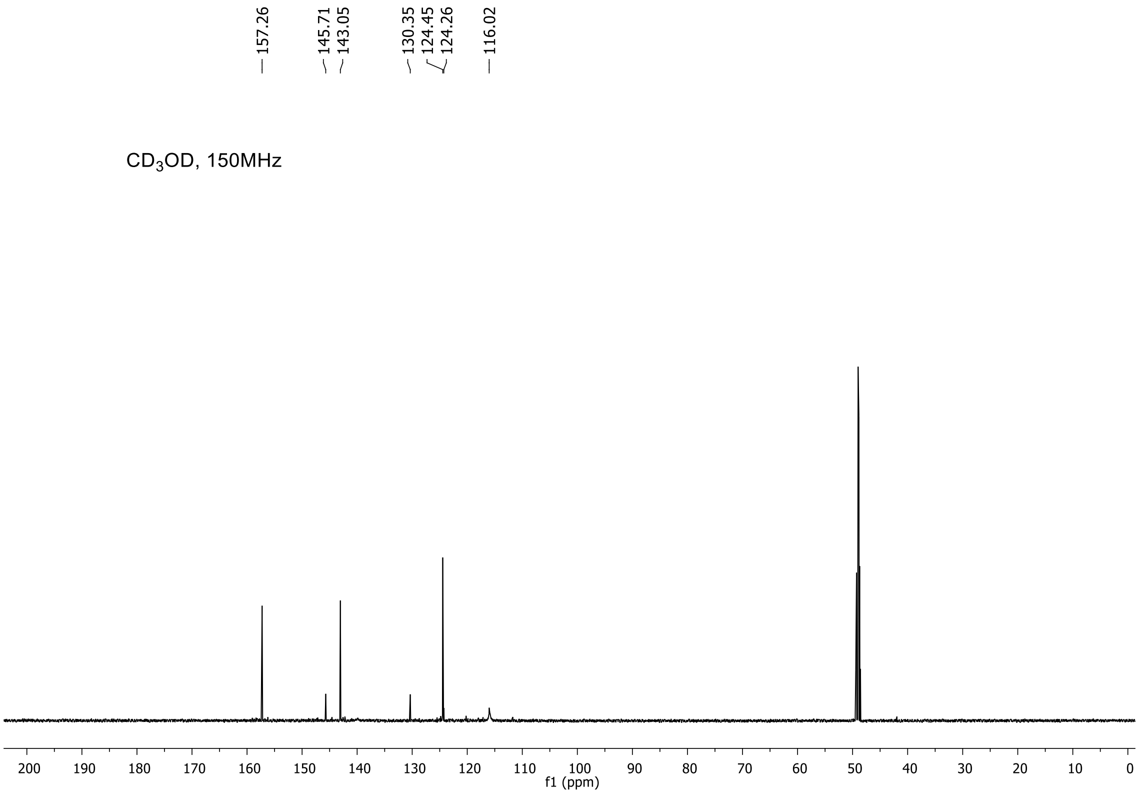

Supplement: Supplementary file 1 [file nanomaterials-10-02405-s001.pdf]
